# Carbon flux through photosynthesis and central carbon metabolism show distinct patterns between algae, C_3_ and C_4_ plants

**DOI:** 10.1038/s41477-021-01042-5

**Published:** 2021-12-23

**Authors:** Haim Treves, Anika Küken, Stéphanie Arrivault, Hirofumi Ishihara, Ines Hoppe, Alexander Erban, Melanie Höhne, Thiago Alexandre Moraes, Joachim Kopka, Jedrzej Szymanski, Zoran Nikoloski, Mark Stitt

**Affiliations:** 1 Max-Planck Institute for Molecular Plant Physiology, Potsdam, Germany; 2 Bioinformatics group, University of Potsdam, Potsdam, Germany; 3 Department of Molecular Genetics, Leibniz Institute of Plant Genetics and Crop Plant Research (IPK), Seeland, OT Gatersleben, Germany; 4 Present Address: School of Plant Sciences and Food Security, Tel Aviv University, Tel Aviv, Israel; 5 Present Address: Crop Science Centre, University of Cambridge, Cambridge, UK

**Keywords:** Plant sciences, Metabolomics, Photosynthesis

## Abstract

Photosynthesis-related pathways are regarded as a promising avenue for crop improvement. Whilst empirical studies have shown that photosynthetic efficiency is higher in microalgae than in C_3_ or C_4_ crops, the underlying reasons remain unclear. Using a tailor-made microfluidics labelling system to supply ^13^CO_2_ at steady state, we investigated in vivo labelling kinetics in intermediates of the Calvin Benson cycle and sugar, starch, organic acid and amino acid synthesis pathways, and in protein and lipids, in *Chlamydomonas reinhardtii*, *Chlorella sorokiniana* and *Chlorella ohadii*, which is the fastest growing green alga on record. We estimated flux patterns in these algae and compared them with published and new data from C_3_ and C_4_ plants. Our analyses identify distinct flux patterns supporting faster growth in photosynthetic cells, with some of the algae exhibiting faster ribulose 1,5-bisphosphate regeneration and increased fluxes through the lower glycolysis and anaplerotic pathways towards the tricarboxylic acid cycle, amino acid synthesis and lipid synthesis than in higher plants.

## Abstract (web summary)

Carbon flux through photosynthesis and central carbon metabolism show distinct patterns between algae, C_3_ and C_4_ plants.

## Main

An increase in agricultural yield of 70% or more is required by 2050 to meet the growing demand of the world population^1,2^. Modern agriculture has witnessed an ~160% increase in global production since the 1950s. This gain was achieved without expanding cropland, by extensive use of fertilizer and plant protection agents as well as improved crop varieties. Future gains face the challenges of shrinking farmland area, an increasingly unpredictable climate and the environmental imperative to use less fertilizer and agrochemicals, and will require new breeding strategies. Most breeding efforts during the Green Revolution addressed pathogen resistance and brought plant architecture and the harvest index close to their theoretical upper limit^3,4^. Indeed, yield is stagnating in most major crops^5–8^.

Photosynthesis is a promising target to increase yield. Photosynthesis is relatively conserved among crops and its efficiency is well below the theoretical maxima^9–11^. The theoretical limit, defined as the fraction of light energy that is captured in conversion of CO_2_ and water to glucose, is ~12% (ref. ^3^). Measured efficiencies reach ~3.5% and ~4.3% for C_3_ and C_4_ plants, respectively, and 5–7% for microalgae, emphasizing the potential of microalgae as a resource for yield improvement^12^. Many factors reduce photosynthetic efficiency, including photochemical limitations and the bifunctionality of ribulose-1,5-bisphosphate carboxylase-oxygenase (RuBisCO). In addition to carboxylation^13^, RuBisCO catalyses a side-reaction with O_2_ that leads to formation of 2-phosphoglycolic acid (2PG), which is recycled via glycine, serine and glycerate to regenerate 3-phosphoglyceric acid (3PGA), with concomitant loss of CO_2_ and ammonium^14^. Several mechanisms have evolved to increase CO_2_ concentration around RuBisCO and decrease the rates of the side-reaction with O_2_. Microalgae pump bicarbonate allowing them to accumulate CO_2_ in microstructures called pyrenoids in which RuBisCO is located^15,16^. C_4_ plants possess a biochemical pump in which bicarbonate is combined with phosphoenolpyruvate (PEP) in externally located mesophyll cells to form four-carbon metabolites that move into the bundle sheath cells and are decarboxylated to generate a high concentration of CO_2_ around RuBisCO^17,18^.

A key question is whether optimizing photosynthetic efficiency will result in higher productivity. Earlier studies with panels of crop genotypes did not find a clear relationship between leaf photosynthetic rate and yield^19^. However, the importance of photosynthesis for yield is underlined by consistent increases in yield in free air elevated [CO_2_] experiments^9^, by the increased yield of transgenic plants that overexpress various carbon (C) assimilation enzymes^11^ and by the finding that enclosed outdoor photobioreactor designs that allow faster photosynthesis often increase algal productivity^20^. Existing efforts to redesign photosynthesis focus on improving light harvesting, light energy conversion and C uptake and conversion, including suppression of photorespiration^4^. Despite its potential impact, little work has addressed photosynthesis-associated metabolism and downstream reactions (often-termed photosynthate investment) as a route to improve yield.

Exploring biodiversity in algae, especially algae from extreme environments, offers means to improve photosynthesis^21,22^. Unique capabilities are more likely to be found in organisms that cope with severe conditions. The green alga *Chlorella ohadii* was recently isolated from a desert biological soil crust, one of the harshest environments on Earth^23^. It exhibits unparalleled resistance to extreme illumination levels (EIL, 3,000 µmol photons m^–2^ s^–1^), high photosynthetic rates^24,25^ and the fastest growth rate ever reported for a photosynthetic organism^26^. *C. ohadii* may provide us with essential information on the photosynthetic machinery and what limits plant performance^26,27^. Physiological and ‘omics studies have uncovered several factors that contribute to its extraordinary resilience under EIL, including a remarkably robust photosystem II, rapid poising of redox status, rapid post-translational redox regulation of protein kinases and reactive oxygen stress (ROS) and heat-shock (HS) responses and thylakoid remodelling^27^.

The key phenotype emerging from changes in gene expression and protein regulation is pathway flux^28^. Due to the complex relationship between protein activity and substrate level, protein and metabolite levels do not provide reliable information about flux. Flux in photosynthesis can be investigated by performing ^13^CO_2_ pulse labelling and analysing the temporal labelling kinetics of metabolic intermediates using chromatography linked to mass spectrometry^28–38^. Here, we monitor the ^13^C-labelling kinetics of >40 intermediates in the Calvin Benson cycle (CBC), photorespiration and central C and nitrogen (N) metabolism, as well as starch, protein and lipids in *C. ohadii* and, for comparison, *C. sorokiniana* and *Chlamydomonas reinhardtii* (two green eukaryotic algae with lower growth rates). The experiments were designed to address two questions: first, are there changes in flux patterns between algal species that could explain the fast growth of *C. ohadii*? Second, do flux patterns in algae differ from those in higher plants? For the latter, we compared labelling kinetics in algae with published and new data from *Arabidopsis thaliana* and *Zea mays* (maize) as model plants for C_3_ and C_4_ photosynthesis, respectively.

## Results

### Seconds-level photosynthetic ^13^C-labelling kinetics in algal cells

**Fig. 1:**
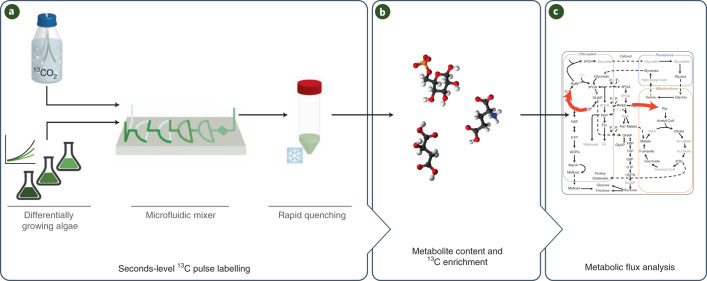
Schematic of the workflow used in this study. **a**, An experimental setup to provide precise short pulses to algal cultures differing in their growth rate on the basis of microfluidic mixer and instantaneous quenching (see Extended Data Fig. 1 for details). **b**, An analytical setup to measure levels and labelling kinetics of intermediates in the CBC and the early steps of the end-product synthesis pathways. **c**, Inspection of labelling temporal kinetics to identify differential flux patterns between algal species.

**Extended Data Fig. 1:**
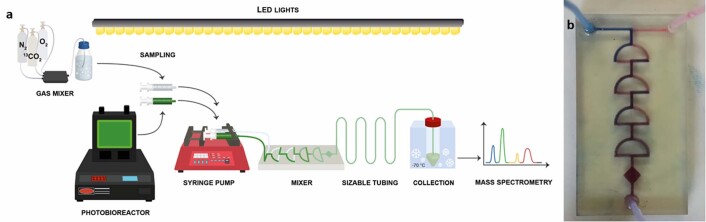
Rapid algal labeling system scheme. (**a**) Fresh medium bubbled with ^13^CO_2_-based synthetic air mixture (see methods) was mixed with fresh algal sample withdrawn from the bioreactor under controlled LED illumination. Solutions were rapidly (<1 s) mixed in a tailor-made transparent chip (**b**), and injected through transparent tubing into 70% methanol solution kept at -70^o^C. Pulse length (5–40 s) was controlled via length of the sizable tubing.

**Extended Data Fig. 2:**
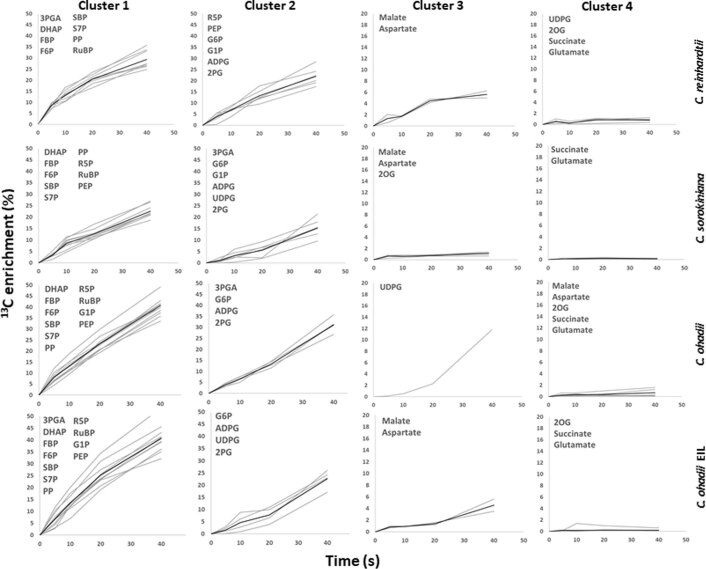
Overview of 13C labeling kinetics by *k*- means clustering for 0- 40 s. Grey lines show mean ^13^C enrichment for individual metabolites quantified by LC- MS/MS (n=3 biologically independent experiments) and black lines show average ^13^C enrichment of all metabolites in the cluster. Note that enrichment of inorganic C in the algal suspension was 50% in this experiment (see Methods).

Since many intermediates in photosynthetic metabolism have turnover times of a few seconds or less, we developed a pipeline built around a microfluidic system that allows precise short pulses and rapid quenching in ambient conditions (Fig. 1, Extended Data Fig. 1 and Supplementary text). To visualize inter- and intra-algal differences we performed *k*-means clustering (*k* = 4, GAP statistics; Supplementary Fig. 1a) on the enrichment kinetics of the 20 analysed intermediates (Extended Data Fig. 2 and Supplementary Tables 1 and 2a). Cluster 1 (mainly CBC intermediates) labelled most rapidly, cluster 2 (intermediates in pathways directly connected to the CBC, like photorespiration and starch and sucrose biosynthesis) and cluster 3 labelled more slowly and there was little labelling of cluster 4 (Extended Data Fig. 2). CBC intermediates showed a near-linear increase in enrichment in all algae and treatments, demonstrating that the microfluidic mixer allows essentially lag-free introduction of ^13^C-labelled inorganic carbon (Ci: CO_2_, HCO_3_^–^, CO_3_^2–^, H_2_CO_3_). Enrichment in cluster 1 rose faster in *C. ohadii* than in *C. sorokiniana* (paired *t-*test for enrichment slopes, *P* = 0.000091) or *C. reinhardtii* (*P* = 0.0032) and faster in *C. ohadii* under EIL than under low light (LL, 100 µmol photons m^–2^ s^–1^) (*P* = 0.0299). Differing enrichment kinetics might reflect different fluxes and/or pool sizes. Most CBC intermediates and PEP had larger pool sizes in the *Chlorella* species than in *C. reinhardtii*, (Supplementary Table 3) showing that the rapid labelling kinetics in *Chlorella* result from higher flux. This is also in qualitative agreement with the rates of oxygen evolution in these cultures (Supplementary Fig. 1).

**Fig. 2:**
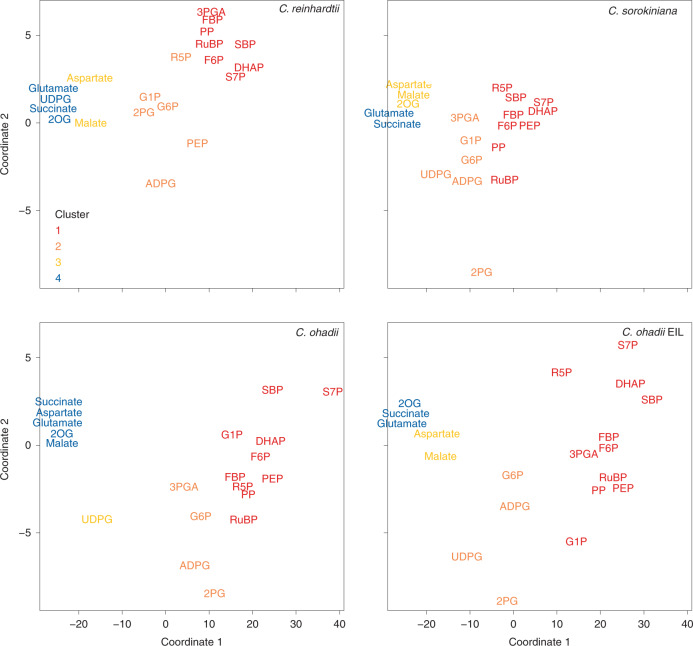
MDS of ^13^C enrichment (%) in the time kinetic pulses of 5–40 s. Metabolites analysed by LC–MS/MS of all algae and treatments were analysed together and then projected on the same coordinates and separated into different panels for clarity of visualization. Colour-coded *k*-means clustering of the data was performed separately for each alga and treatment (Supplementary Table 2a and Extended Data Fig. 2): red, orange, yellow and blue, denote intermediates in clusters 1, 2, 3 and 4, respectively, which showed an increasingly slow rise in enrichment.

The clusters were visualized by multidimensional scaling (MDS; the spread of the different clusters across the corresponding two-dimensional space reflects different parameters of the enrichment–time curves; for example, slope and levels) (Fig. 2). Metabolites in clusters 1 and 2 showed wider spread in *C. ohadii* than in *C. sorokiniana* and *C. reinhardtii*; for example, sedoheptulose-1,7-bisphosphate (SBP), sedoheptulose-7-phosphate (S7P) and (in EIL) dihydroxyacetone phosphate (DHAP) separated further from other CBC intermediates in *C. ohadii* than in the other two algae. Glucose-1-phosphate (G1P) showed similar labelling to CBC intermediates in *C. ohadii* but clustered with further intermediates of starch and sucrose synthesis in the other two algae. PEP grouped with CBC intermediates (cluster 1) in both *Chlorella* species but not in *C. reinhardtii*.

Comparison of labelling kinetics between algae and plants was made possible thanks to the experimental design that guaranteed immediate and equivalent enrichment of all Ci species (Methods). Enrichment of several CBC intermediates rose faster in algae than in *Arabidopsis* (3PGA, DHAP, fructose-1,6-bisphosphate (FBP), SBP, ribulose 1,5-bisphosphate (RuBP); Supplementary Tables 1 and 4; data from ref. ^31^ or measured here) and faster in *C. ohadii* than in maize (DHAP, FBP, SBP, ribose-5-phosphate (R5P); Supplementary Tables 1 and 4; extracted from ref. ^32^).

To facilitate interspecies comparison, we regressed the enrichment of each intermediate against enrichment of DHAP and computed the correlation coefficient and slope (Supplementary Fig. 3, summarized in Supplementary Table 4). This reveals the extent to which each intermediate is in isotopic equilibrium with DHAP. We chose DHAP rather than 3PGA, which is the first CO_2_ fixation product and immediately upstream from DHAP, because in algal samples 3PGA consistently exhibited lower apparent enrichment than did DHAP (Supplementary Table 1); this may be due to subcompartmentation and/or errors in 3PGA isotopomer quantification due to slight peak spreading. The correlation coefficients of DHAP to other CBC intermediates in algae were very high (>0.95) for all CBC intermediates tested (Supplementary Fig. 2; see Supplementary Table 4 for slopes of the regressions). Under LL, slopes for fructose-6-phosphate (F6P), SBP, S7P, R5P and RuBP were higher in *C. ohadii* (0.97, 0.97, 1.19, 0.93 and 0.97, respectively) than in *C. sorokiniana* (0.77, 0.82, 1.03, 0.70 and 0.84) or in *C. reinhardtii* (0.72, 0.94, 0.94, 0.81 and 0.70). In *C. ohadii* under EIL, all slopes were essentially identical to DHAP (0.96, 1.11, 1.29, 0.97, 1.03 for F6P, SBP, S7P, R5P and RuBP, respectively) pointing to especially rapid CBC flux in these conditions.

In *Arabidopsis* and maize, the slopes on DHAP for CBC metabolites were similar (FBP in maize but not *Arabidopsis*, SBP, S7P, pentose-phosphates (PP: xylulose-5-phosphate (Xu5P) and ribulose-5-phosphate (Ru5P)), RuBP) or slightly lower (R5P and F6P) than in algae (Supplementary Table 4; calculated from refs. ^31,32^ and Extended Data Fig. 2). This points to similar labelling patterns of CBC metabolites in algae and plant leaves. The higher enrichment of S7P compared to other CBC intermediates found in *C. ohadii* (Fig. 2 and Extended Data Fig. 2) has also been seen in leaves of *Arabidopsis*, maize (Supplementary Tables 1 and 4) and, recently, *Camelina sativa*^39^. The slower rise of F6P and PP might be due to compartmentation, either between the chloroplast and cytosol of photosynthetic cells and/or between photosynthetic and non-photosynthetic cells (below and Supplementary text).

Enrichment in intermediates of starch and sucrose synthesis (mainly cluster 2) rose slightly more slowly than for CBC intermediates (Extended Data Fig. 2). Labelling of starch and sucrose precursor intermediates was fastest in *C. ohadii* in EIL, followed by *C. ohadii* in LL, and *C. sorokiniana* and was slowest in C*. reinhardtii*. Enrichment in glucose-6-phosphate (G6P) rose more slowly than F6P, probably reflecting slow labelling of cytoplasmic pools as described in higher plants (Supplementary text).

Microalgae possess a CO_2_-concentrating mechanism (CCM) which decreases the side-reaction of RuBisCO with O_2_ that leads to formation of 2PG (Main text); 2PG was nevertheless rapidly labelled in algae (Extended Data Fig. 2; cluster 2). The slope of the regression on DHAP was higher in the *Chlorella* species (0.78–0.90) than in *C. reinhardtii* (0.48), resembled *Arabidopsis* (0.76) and was higher than maize (0.40) (Supplementary Table 4). Levels of 2PG were significantly lower in *C. ohadii* and *C reinhardtii* than in *C. sorokiniana* in LL and significantly higher in *C. ohadii* in EIL than in LL (Supplementary Fig. 3). Levels of 2PG per unit dry weight (DW) in algae under LL were 10–25 times lower than in *Arabidopsis* and 2.5–5 times lower than in maize (Supplementary Fig. 4; estimated from this study for *Arabidopsis* and from ref. ^32^ for maize, assuming 90–92% water content^40^). The 2PG levels in *C. ohadii* under EIL resembled those in maize.

Enrichment in PEP was identical to that of early CBC intermediates in *C. ohadii* (slope of the regression on DHAP = 1.042 and 1.06 under LL and EIL, respectively; Fig. 2 and Supplementary Fig. 3). This underlies the close location of PEP and RuBP in the MDS analysis of the *C. ohadii* dataset (Fig. 2). The slope of the regression of PEP on DHAP was slightly lower in *C. sorokiniana* (0.91) and much lower in *C. reinhardtii* (0.79), *Arabidopsis* (0.76) and, especially, the C_4_ species maize (0.25) (Supplementary Fig. 3 and Supplementary Table 4). Slower labelling of PEP in *Arabidopsis* might, in principle, be due to a large pool of unlabelled or slowly labelling PEP in non-photosynthetic cells. It is, however, unlikely that such cells contain a large pool of PEP but not of other glycolytic intermediates (Supplementary text). Furthermore, many other features of our study point to rapid flux from the CBC to PEP in algae (below). In maize, flux to PEP is dominated by pyruvate returning from the bundle sheath cells, where decarboxylation of malate and aspartate occurs. Crucially, the C1–3 positions of malate, aspartate and metabolites deriving from them, like pyruvate, are initially unlabelled. Flow of ^13^C from the CBC into PEP is therefore diluted by a massive influx of unlabelled C from the carbon concentrating shuttle^32^ (Supplementary text).

Enrichment in malate rose much more slowly than in CBC intermediates (Fig. 2 and Extended Data Fig. 2). Enrichment rose faster in *C. reinhardtii* under LL and *C. ohadii* under EIL than in *C. ohadii* and *C. sorokiniana* under LL (slope of regression on DHAP = 0.186, 0.123, 0.030 and 0.048, respectively). Aspartate followed a similar pattern (0.143, 0.078, 0.036 and 0.044, respectively). The faster rise in enrichment in *C. reinhardtii* might be attributed to 5–25 smaller pool sizes than in the *Chlorella* species (Supplementary Table 3). Comparison with the slopes in *Arabidopsis* (malate 0.002–0.02, aspartate 0.02) and maize (malate 0.23, aspartate 0.76) revealed that labelling of malate in algae was faster than in C_3_ photosynthesis but slower than in C_4_ photosynthesis^21^ (Supplementary Table 4). Similar conclusions were reached by comparing ^13^C enrichment at 40 s (Supplementary Table 1).

The label in malate and aspartate at 0–40 s in algae probably reflects flux from rapidly labelled PEP. The dominant labelled isotopomer for malate and aspartate in all algae and conditions was *m* + 1 (mass), as expected because PEP carboxylase (PEPC) adds H^13^CO_3_^−^ to PEP. However, the *m* + 2 isotopomer of aspartate and malate appeared by 40 s, in parallel with *m* + 1 becoming the dominant isotopomer of PEP (Supplementary Fig. 5). PEP may be rapidly converted to downstream metabolites that were not detected by reverse phase liquid chromatography linked to tandem mass spectrometry (LC–MS/MS) or that accumulated such small amounts of ^13^C that they could not be reliably investigated. These observations prompted us to investigate labelling kinetics over longer time scales.

### Long-term labelling of metabolites in different algae

For longer labelling (minutes to hours), we used direct bubbling of running cultures. The main aim was to investigate flux into major products. We first analysed sugars, organic and amino acids using gas chromatography linked to mass spectrometry (GC–MS)^41^.

**Fig. 3:**
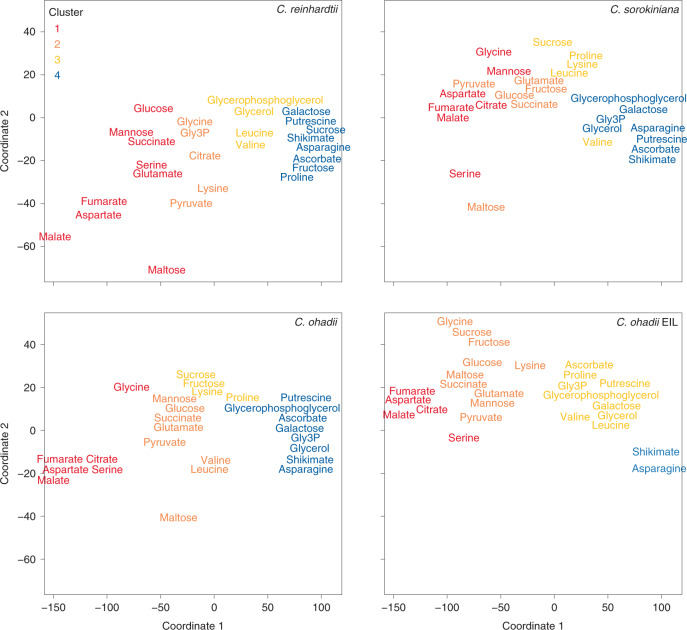
MDS of ^13^C enrichment (%) in the time kinetic pulses of 15–300 min. Metabolites analysed by GC–MS of all algae and treatments were analysed together and then projected on the same coordinates and separated into different panels for clarity of visualization. Colour-coded *k*-means clustering of the data was performed separately for each alga and treatment (Supplementary Table 2b and Extended Data Fig. 3): red, orange, yellow and blue, denote intermediates in clusters 1, 2, 3 and 4, respectively, which showed an increasingly slow rise in enrichment.

**Extended Data Fig. 3:**
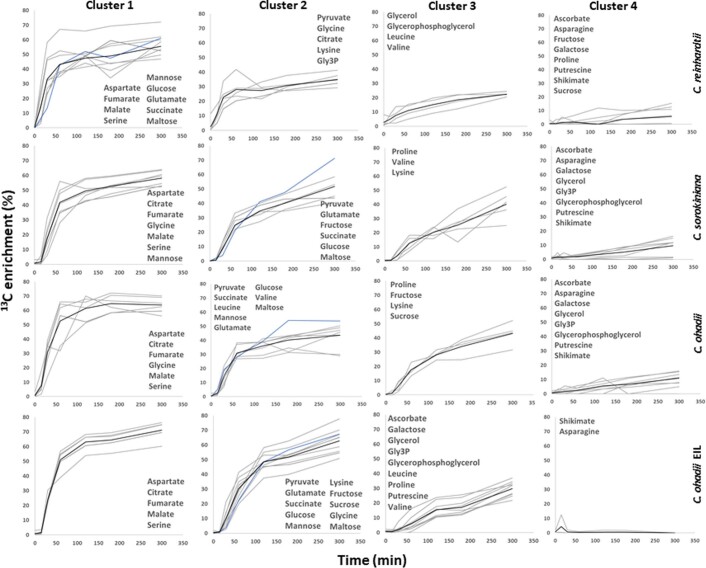
Overview of 13C labeling kinetics by *k*- means clustering for 0- 300 min. Grey lines show mean ^13^C enrichment for individual metabolites quantified by GC- MS (n=3 biologically independent experiments) and black lines show average ^13^C enrichment of all metabolites in the cluster. Maltose is highlighted in blue.

We again performed *k*-means clustering based on Euclidian distance (*k* = 4, GAP statistics; Supplementary Fig. 1b). Whereas near-linear labelling kinetics between 0 and 40 s facilitated direct comparison of ^13^C flow (above; Fig. 2, Extended Data Fig. 2 and Supplementary Table 1), labelling kinetics were more complex at 15–300 min (Extended Data Fig. 3 and Supplementary Table 2b). They are summarized in the MDS representation (Fig. 3). Cluster 1 was characterized by a quite rapid rise in enrichment, which in most cases plateaued by 60 min or earlier. Clusters 2, 3 and 4 showed progressively slower and less complete labelling (Extended Data Fig. 3). Enrichment in cluster 1 plateaued at 40–60% in *C. reinhardtii*, 50–60% in *C. sorokiniana*, 50–70% in *C. ohadii* under LL and 65–75% in *C. ohadii* under EIL (Extended Data Fig. 3). Higher enrichment in *C. ohadii* compared with the other algae indicates that a larger fraction of its total metabolite pool is involved in photosynthetic metabolism, especially in EIL. This is probably linked to the higher photosynthesis rate but may also be affected by compartmentation (Supplementary Fig. 2). In the MDS representation, cluster 4 intermediates in *C. ohadii* at EIL shifted towards the position taken by cluster 3 intermediates in all algae in LL (Fig. 3), indicative of higher fluxes. In *C. reinhardtii*, some metabolites, including glucose, mannose, succinate and glutamate, shifted to the position occupied by cluster 1 metabolites in the *Chlorella* species in LL (Fig. 3). This may partly reflect smaller pool sizes in *C. reinhardtii* (Supplementary Table 3).

Cluster 1 included malate, aspartate, the photorespiratory intermediates glycine (with the exception of *C. reinhardtii* and *C. ohadii* under EIL) and serine and several tricarboxylic acid (TCA) cycle intermediates (Fig. 3 and Extended Data Fig. 3). In line with seconds-level ^13^C enrichment patterns, the rise in enrichment of malate during the first 60 min resembled that in maize and was considerably faster than in *Arabidopsis* (Supplementary Table 5). Enrichment in aspartate rose more slowly than maize but faster than *Arabidopsis* (except for *C. sorokiniana*). Enrichment of citrate, fumarate and succinate rose early (clusters 1 and 2) in all algae (Fig. 3 and Extended Data Fig. 3). Succinate and fumarate rose faster than in *Arabidopsis* or maize (Supplementary Table 5; citrate data are not available for *Arabidopsis* or maize). These TCA intermediates were mostly in clusters 1 and 2 and were localized quite close to malate and aspartate in the MDS analysis. Intermediate 2-oxoglutarate (2OG) was not reliably detected in our analyses but probably had a similar pattern because enrichment rose rapidly in glutamate in all algae (clusters 1 or 2; Fig. 3, Extended Data Fig. 3 and Supplementary Table 5). These results point to rapid flux via PEPC towards an anabolism-directed TCA cycle, rather than towards malate decarboxylation to deliver CO_2_ to RuBisCO, as is the case in C_4_ photosynthesis.

Enrichment of glycerol 3-phosphate (Gly3P) rose faster in *C. reinhardtii* (cluster 2) than in the *Chlorella* species under LL (cluster 4) possibly due to the smaller pool in *C. reinhardtii*. Gly3P was among the most abundant metabolites in these algae (Supplementary Table 3; similar was previously reported for *C. reinhardtii*^42^) with one to two orders higher levels than previously reported in *Arabidopsis* (Table 1, extracted from ref. ^43^).

**Table 1:** Comparative levels of C pools and selected metabolites in the algal cultures and *Arabidopsis*

	Sucrose (µmol g^−1^ DW)	Galactose (µmol g^−1^ DW)	Starch (µmol C6 eq. g^−1^ DW)	Protein content (mg g^−1^ DW)	Lipid content (mg FA g^−1^ DW)	Gly3P (µmol g^−1^ DW)
*C. reinhardtii*	0.05 ± 0.03^e^	2.8 ± 6.70^a^	114 ± 23^c^	248 ± 18^a^	189 ± 16^b^	8.1 ± 2.0^b^
*C. sorokiniana*	3.4 ± 0.2^d^	1.1 ± 0.10^b^	432 ± 19^b^	135 ± 6^c^	211 ± 15^b^	32.3 ± 9.4^a^
*C. ohadii*	5.1 ± 0.8^b^	3.0 ± 1.30^a^	889 ± 69^a^	160 ± 27^b^	198 ± 26^b^	49.1 ± 2.4^a^
*C. ohadii* EIL	4.4 ± 0.2^c^	3.6 ± 1.10^a^	446 ± 49^b^	184 ± 8^b^	476 ± 19^a^	34.3 ± 7.4^a^
*Arabidopsis*	11.1 ± 1.1^e^	0.6 ± 0.18^b^	380 ± 20^b^	178 ± 5^d^	38 ± 0.5 (leaf)^c^ 350 ± 9 (seed)	0.22 ± 0.03^b^
*P* value	4.17 × 10^−50^	2.95 × 10^−15^	7.96 × 10^−57^	2.38 × 10^−20^	2.06 × 10^−14^	3.08 × 10^−8^

For *Arabidopsis*, protein content and synthesis rate was extracted from ref. ^58^. Lipid content (fatty acids) was extracted from ref. ^50^ for leaves and ref. ^97^ for seeds. One-way ANOVA (sum-of-squares type II) for the factor species, followed by Tukey’s HSD post-test analyses were performed independently for each trait. *P* values indicate the probability of the null-hypothesis from the ANOVA test, whereas superscript letters a–e denote statistically significant different groups of species per each trait (at a significance level of *P* < 0.05).

Enrichment in sucrose rose much faster in the *Chlorella* species (cluster 3) than in *C. reinhardtii* (cluster 4) under LL and rose even faster in *C. ohadii* under EIL (cluster 2; Extended Data Fig. 3 and Supplementary Table 5). The sucrose pool was two orders of magnitude smaller in *C. reinhardtii* than in the *Chlorella* species (Supplementary Table 3). Accordingly, movement of ^13^C into sucrose was negligible in *C. reinhardtii* (Extended Data Fig. 3, Table 2 and Supplementary Table 5). With the exception of *C. ohadii* under EIL, enrichment in sucrose rose more slowly in algae than in *Arabidopsis* or maize (Supplementary Table 5).

Galactose was the predominant reducing sugar in terms of pool size in all three algae (Supplementary Table 3). Labelling of galactose (Fig. 3, Extended Data Fig. 3 and Supplementary Table 5) was slow (cluster 4) in all algae, except for *C. ohadii* under EIL which exhibited both faster labelling (cluster 3) and a larger pool (Supplementary Table 3) than under LL. As no data for galactose levels are available from refs. ^31,32^, it was measured in new samples; galactose was 1.4–6-fold lower in *Arabidopsis* than in algae (Table 1 and Supplementary Table 3).

Enrichment in glucose and fructose rose faster in algae than in higher plants, also relative to sucrose (Supplementary Table 5). In all algae, the labelling kinetics and isotopomer pattern of glucose resembled that of maltose (Extended Data Fig. 3, Supplementary Fig. 6 and Supplementary Table 2b). Maltose is a product of starch degradation. The rise in enrichment in glucose and maltose slowed down or plateaued from about 60–120 min on (Extended Data Fig. 3), with 30–60% of the maltose pool still being the unlabelled isotopomer (Supplementary Fig. 6). These results pointed to a large flux from starch through maltose to glucose. We therefore explored labelling of starch.

### Rates of starch synthesis and degradation

**Extended Data Fig. 4:**
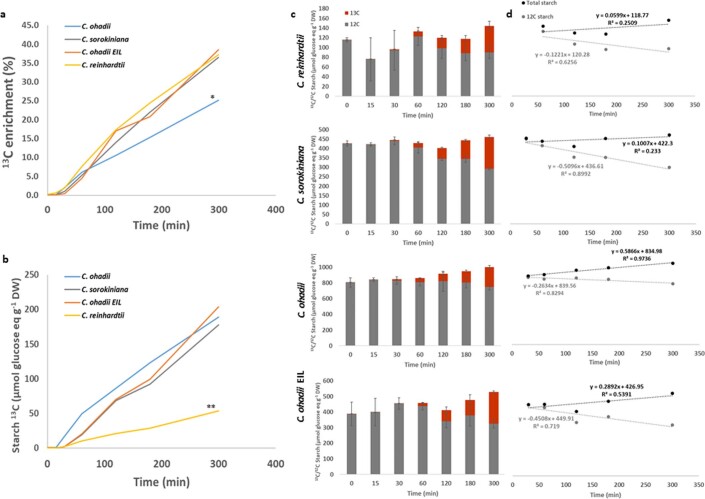
Kinetics of 13C labeling in starch. ^13^C enrichment (**a**) and total starch content (**b**) in digested starch analysed by LC–MS/MS from all three algae in LL and *C. ohadii* in EIL; Data are mean ± SD (n=3 biologically independent experiments). For inter-algal comparisons, slopes for each alga were compared to *C. ohadii* at LL (paired two-tailed t-test; *, P = 6xE^-3^; **, P =4xE^-4^).

Starch is often a major C reserve in chlorophyte algae^44,45^. In plants, foliar starch is often a major C reserve, which is accumulated in the light and degraded in the dark to provide sugars for growth and metabolism at night^40,46,47^. ^13^C enrichment in starch rose more slowly in *C. ohadii* than in *C. reinhardtii* and *C. sorokiniana* under LL or *C. ohadii* under EIL (Extended Data Fig. 4a). Starch content per unit DW was highest in *C. ohadii* under LL and about twofold, eightfold and twofold lower in *C. sorokiniana* and *C. reinhardtii* under LL and *C. ohadii* under EIL (*P* = 7.37 × 10^−7^, 1.14 × 10^−8^ and 1.41 × 10^−7^, respectively; Extended Data Fig. 4c). ^13^C accumulated in starch at similar rates in *C. sorokiniana* under LL, *C. ohadii* under LL and C. *ohadii* under EIL and three times more slowly in *C. reinhardtii* under LL (Extended Data Fig. 4b and Table 2; *P* = 4.07 × 10^−5^).

In all algae, ^13^C accumulation was accompanied by a significant decrease in the amount of ^12^C in starch (Extended Data Fig. 4c,d). The labelling kinetics of maltose and glucose (above) are consistent with maltose and glucose deriving in part from degradation of newly synthesized starch and partly from degradation of preformed and hence unlabelled starch. The latter may provide a ^12^C source that prevents full enrichment of other metabolites (Supplementary text).

### Rates of protein synthesis

**Extended Data Fig. 5:**
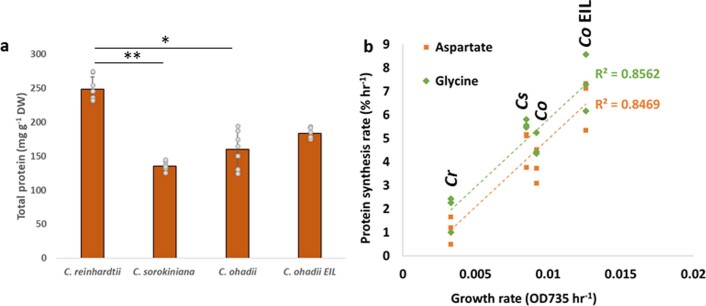
Labeling of protein during pulses of ^13^CO_2_. (**a**) Total protein levels per unit DW. Protein pools were extracted from remaining pellets of the MTBE metabolite extractions for time points 15–300 min, determined by the Bradford assay, and divided by DW per sample. Data are mean ± SD of all time points per alga (n=3 biologically independent experiments). For inter-algal comparisons, all algae in LL were pairwise compared (paired two-tailed t- test;*, P= 2.2xE^-5^; **, P =6.1xE^-7^). (**b**) Protein synthesis rates (PSR, % h^-1^) calculated for glycine and aspartate (see Table S6) and plotted against growth rates (OD735 h^-1^) of the four algae and conditions. PSR were calculated from the labeling rates of protein-bound glycine or aspartate analysed by GC–MS, divided by time interval (120-300 min) and the mean enrichment (%) of their free form (see [58]). *Cr* = *C. reinhardtii, Cs = C. sorokiniana, Co = C. ohadii*.

We next examined ^13^C accumulation in protein, which in a growing single-celled alga can be considered an end-product of photosynthesis. Protein content per unit DW was 46% and 36% lower in *C. sorokiniana* and *C. ohadii* than in *C. reinhardtii* (*P* = 2.18 × 10^−5^ and 6.15 × 10^−7^, respectively; Extended Data Fig. 5a). Protein synthesis rates (PSR, percentage of protein synthesized per h; see Methods for how this was measured and calculated) in *C. sorokiniana* and *C. ohadii* were 2.0- to 5.1-fold higher than in *C. reinhardtii* (4.7–5.6% h^−1^ and 3.8–4.7% h^−1^, compared to 1.1–1.9% h^−1^). PSR correlated positively with growth rate across the three algae in LL (*R*^2^ > 0.86, Extended Data Fig. 5b). PSR in *C. ohadii* under EIL was 1.5–1.7-fold higher than under LL (Supplementary Table 6), slightly above the 1.4-fold higher growth rate. PSRs in the *Chlorella* species were 2.3- to 3.9-fold higher than in *Arabidopsis* (data not available for maize).

### Qualitative estimation of lipid synthesis

**Extended Data Fig. 6:**
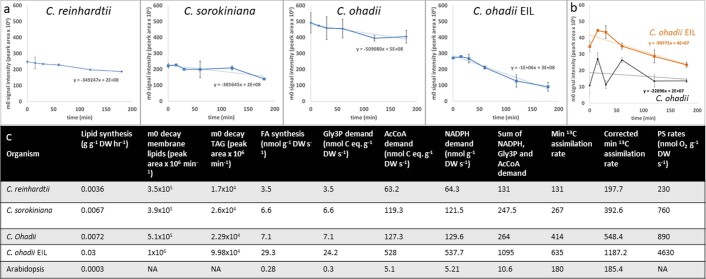
Lipid synthesis. (**a**) m0 decay curves for membrane lipids in all algae and conditions. The m0 peak areas for all labelled PE, MGDG and DGDG species were summed for each of the algae and conditions for each ^13^CO_2_ pulse time point. Data are mean ± 95% confidence intervals (n=3 biologically independent replicates). (**b**) m0 decay curves for TAG in *C. ohadii* and *C. ohadii* EIL. The m0 peak areas for all labelled TAG were summed for each of the algae and conditions for each ^13^CO_2_ pulse time point and are presented here for *C. ohadii* under LL and EIL to illustrate the large difference between the light treatments. Data are mean ± 95% confidence intervals (n=3 biologically independent replicates). Data for panels (a) and (**c**) were generated from the experiments used for GC–MS, starch and protein analyses in Figures 3, 5, Extended Data Fig. 3, and Tables 1,2 and Supplementary Table 3–6. Estimated rates of lipid synthesis and associated fluxes. For algae, the total lipid synthesis rate was estimated from the DW increase over time (Supplementary Figure 7) and the lipid fraction of DW (Table 1). The calculation assumes that lipid content is approximately constant during growth of cultures up to the time of sampling, and that there is negligible turnover of lipids. Qualitative support for the estimated rates of total lipid synthesis rates is provided by the rate of decay of the m0 isotopomers of membrane lipids (derived slopes from panel a) and TAG (derived slopes from panel b and not shown). For Arabidopsis, total lipid synthesis was estimated based on relative growth rate of the rosette [49] and leaf lipid fraction per DW [50]. FA synthesis rates in algae and Arabidopsis are estimated from total lipid synthesis rates using the simplifying assumption that all FA are C18:0 species. FA synthesis rate in Arabidopsis was also estimated to provide qualitative support using the following assumptions: lipid accumulation rate corresponds to 6% of total C fixation (Table 2) in ^14^C-labeling experiments in Arabidopsis leaves [51], and (based on C18:0 FA species) 0.6 nmol FA g^-1^ DW s^-1^, which is about 2-fold higher than estimated by the first method, but still much lower than the estimated flux to FA in algae. Demand for Gly3P and Acetyl- CoA are calculated based on 3:1 and 1:9 ratio of Gly3P and Acetyl-CoA to FA, respectively. NADPH demand in lipid synthesis is calculated assuming net consumption of 1 NADPH per Gly3P synthesized by reduction of DHAP plus net consumption of 2 NADPH per Acetyl-CoA added. The minimum (Min) ^13^C assimilation rate is taken from Table 2 (includes ^13^C assimilation between 0 and 40 sec into water- soluble intermediates detected by LC–MS/MS) was corrected (Corrected min) by adding on the estimated flux of C to FA. These corrected estimates are about 20, 50, and 40% lower than the rate of O_2_ evolution (taken from Table 2). in *C. reinhardtii*, *C. sorokiniana*, *C. ohadii* in LL but about 75% lower in *C. ohadii* in EIL. Much of the remaining discrepancy can be explained by NADPH consumption in lipid synthesis, except for *C. ohadii* in EIL.

Lipids represent a major C sink in algae^48^, which have a much higher lipid content than plant leaves (Table 1). We took two approaches to estimate flux to lipids. The first was a bulk index for lipid synthesis per DW on the basis of biomass fraction of lipid (Table 1) and growth rate (Supplementary Fig. 7). This indicated higher rates of lipid synthesis in the *Chlorella* species than in *C. reinhardtii* under LL and threefold to ninefold higher rates in *C. ohadii* under EIL than under LL (Extended Data Fig. 6). In *Arabidopsis* leaves, a similar approach (supported by lipid ^14^C-labelling experiments; refs. ^49–51^) gave estimates eightfold to 100-fold lower than algae (Extended Data Fig. 6). To provide qualitative support, the lipid fraction of ^13^CO_2_-labelled samples was analysed by LC–MS. This revealed substantial labelling of membrane lipids and triacylglycerol (TAG) in *C. reinhardtii*, *C. sorokiniana* and *C. ohadii* under LL and even faster labelling in *C. ohadii* under EIL (Extended Data Fig. 6).

### Comparison of estimated fluxes to end-products

Tables 1 and 2 summarize the estimated contents and fluxes to sucrose, glucose, galactose, Gly3P, starch and protein for the three algae in LL and *C. ohadii* in EIL, as well as values for *Arabidopsis* estimated from published and new data. Compared to *Arabidopsis*, algae contain less sucrose, similar contents of starch and protein and higher galactose and Gly3P.

**Table 2:** Comparative rates and estimates of photosynthesis, synthesis rates of sucrose and starch in the algal cultures and *Arabidopsis*

		(nmol C eq. g^−1^ DW s^−1^)										
	PS rates (nmol O_2_ g^−1^ DW s^−1^)	Minimum ^13^C assimilation rate	Sucrose synthesis rate	Minimum flux to galactose	Minimum flux to glucose	Minimum flux to Gly3P	Protein synthesis rate	Minimum flux over PEPC	Starch synthesis rate	Starch degradation rate	Net starch accumulation	Estimated % of ^12^C supply from starch
*C. reinhardtii*	230 ± 61^c^	131 ± 17^d^	0 ± 0^c^	1.9 ± 0.2^c^	0.3 ± 0.1^c^	4.0 ± 2.0^a^	45 ± 18^b,c^	7 ± 2^c^	19 ± 3^c^	12.2 ± 1.0 ^c,d^	6.8	9.31
*C. sorokiniana*	760 ± 90^b^	267 ± 71^c^	17 ± 0.9^b^	0.7 ± 0.1^d^	0.6 ± 0.1^c^	5.8 ± 2.8^a^	72 ± 2^b^	70 ± 12^a^	61 ± 3^b^	51.0 ± 10.0^a^	10	19.10
*C. ohadii*	890 ± 79^b^	414 ± 35^b^	35 ± 5^b^	2.8 ± 0.2^b^	1.9 ± 0.2^b^	4.2 ± 3.6^a^	73 ± 7^b^	48 ± 15^a,b^	85 ± 8^a^	26.0 ± 7.0^b,c^	59	6.28
*C. ohadii* EIL	4,630 ± 180^a^	635 ± 14^a^	61 ± 3^a^	6.1 ± 0.5^a^	4.4 ± 0.6^a^	3.1 ± 0.9^a^	186 ± 31^a^	29 ± 14^b,c^	74 ± 3^a^	45.0 ± 12.0^a,b^	29	7.09
*Arabidopsis*	NA	180 ± 5 ^c,d^	28 ± 3*^,b^	NA	2 ± 0.1^b^	NA	19 ± 1^c^	16 ± 4^c^	54 ± 4^b^	0 ± 0^d^	54	NA
*P* value	1.51 × 10^−4^	7.4 × 10^−6^	4.07 × 10^−8^	4.37 × 10^−6^	3.2 × 10^−6^	0.632	2.33 × 10^−8^	2.13 × 10^−4^	7.99 × 10^−4^	5.81 × 10^−9^		

For algae, PS (photosynthesis) rates are taken from Supplementary Fig. 4. The minimum C fixation rate is estimated from summing ^13^C found in all metabolites at 5, 10, 20 or 40 s (estimated for each metabolite as ^13^C enrichment multiplied by metabolite pool size and the number of C atoms in the molecule (Extended Data Fig. 2 and Supplementary Tables 1 and 3) and extracted from the highest ^13^C incorporation rate obtained among these time points). Minimum flux at PEP is estimated in a similar manner considering only ^13^C found in malate and aspartate. In both cases, the estimate considers that ^13^CO_2_ enrichment in the medium was only 50%. Sucrose synthesis rates are estimated from the slope of multiplying enrichment (Supplementary Table 5) and pool size (Supplementary Table 3) over time at 30–120 min following ^13^C-labelling and considering a ratio of 12:1 for number of C atoms in sucrose. Minimum fluxes to glucose and galactose were estimated in a similar manner and considering a ratio of 6:1 for number of C atoms. The relatively low enrichment in galactose and its linear slope at 0–300 min (Extended Data Fig. 3 and Supplementary Table 5) imply that any underestimation is minor. Starch synthesis and degradation rates are estimated from starch content multiplied by the slopes presented in Supplementary Fig. 14d for ^13^C- and ^12^C-starch, respectively, and considering a ratio of 6:1 for number of C atoms in glucose. Protein synthesis rate is calculated from the rate of protein synthesis (% h^**−**1^; Supplementary Table 6) multiplied by protein content (mg g^−1^ DW; Extended Data Fig. 5a) assuming that C accounts for 43% of the weight in protein. Estimated percentage of ^12^C supply from starch was calculated as ratio between starch degradation and ^13^C assimilation rates and net starch accumulation as the difference between starch synthesis and degradation rates.

For *Arabidopsis*, photosynthesis and starch synthesis rates (for ZT (Zeitgeber time) 0–12 h) were extracted from refs. ^59,57^ for 105 µmol photons m^−2^ s^−1^, respectively. Starch and sucrose levels are taken from ref. ^57^ and ref. ^31^, respectively. Minimum flux at PEP is estimated as above on ^13^C found in malate and aspartate at 0–60 s from ref. ^31^. *Sucrose synthesis rates were estimated as above for 3–60 min following ^13^C-labelling from ref. ^31^ and are an underestimate as they exclude ^13^C sucrose exported from the leaves. On the basis of ^14^C-labelling studies of individual leaves in *Arabidopsis* and ^14^C export to the rest of the plant, sucrose synthesis rate is likely to be ~20% higher^62,63^. Flux to glucose was estimated as above from ref. ^31^. Starch degradation rate was calculated for ZT 0.25–2 as above from ref. ^57^ and net starch accumulation as the difference between starch synthesis and degradation rates. Protein synthesis rate was extracted and calculated as above from ref. ^58^.

One-way analysis of variance (ANOVA) (sum-of-squares type II) for the factor species, followed by Tukey’s HSD post-test analyses were performed independently for each trait. *P* values indicate the probability of the null-hypothesis from the ANOVA test, whereas superscript letters denote statistically significant different groups of species per each trait (at a significance level of *P* < 0.05). NA, not available.

Flux to sucrose was undetectable in *C. reinhardtii*, substantial in *C. sorokiniana* and highest in *C. ohadii* where it was in the same range as *Arabidopsis* (Table 2). The estimated flux in *Arabidopsis* may be an underestimate due to ^13^C sucrose export and utilization for growth. Except in *C. reinhardtii*, flux to glucose was lower than flux to sucrose. Flux to galactose exceeded flux to glucose in all three algae but was lower than flux to sucrose in the *Chlorella* species (Table 2). In these continuous illumination conditions, the rate of starch degradation approaches that of starch synthesis in algae (Table 2), resulting in slower net starch accumulation than in *Arabidopsis* (except for *C. ohadii* in LL). Importantly, both flux to starch and combined flux to sugars were lower than flux to protein in algae, whereas both were higher than flux to protein in *Arabidopsis*.

The initial rate of accumulation of ^13^C in malate and aspartate was used to estimate a minimum flux at PEPC (this is an underestimate due to rapid movement of ^13^C through into other organic acids and amino acids, as well as lipids; below). In algae, minimum estimated flux at PEPC was of the same order as estimated flux to sucrose or starch (Table 2), whereas in *Arabidopsis* it was much lower than flux to sucrose or starch. *C. ohadii* in EIL appears to be an exception but our estimate may be a large underestimate because a considerable amount of ^13^C has probably moved beyond malate and aspartate. For example, minimum estimates of flux to organic and amino acids quantified by gas chromatography linked to mass spectrometry (GC–MS) (including pyruvate, citrate, glutamate, proline and glycine) were 9.8 ± 0.8 and 32.5 ± 3 nmol C eq. g^−1^ DW s^−1^ in *C. ohadii* under LL and EIL, respectively (see Methods for calculation). These rates alone (to which fluxes to lipids and protein should be added) point to flux at PEPC being higher than flux to sucrose or starch in *C. ohadii* in EIL.

**Fig. 4:**
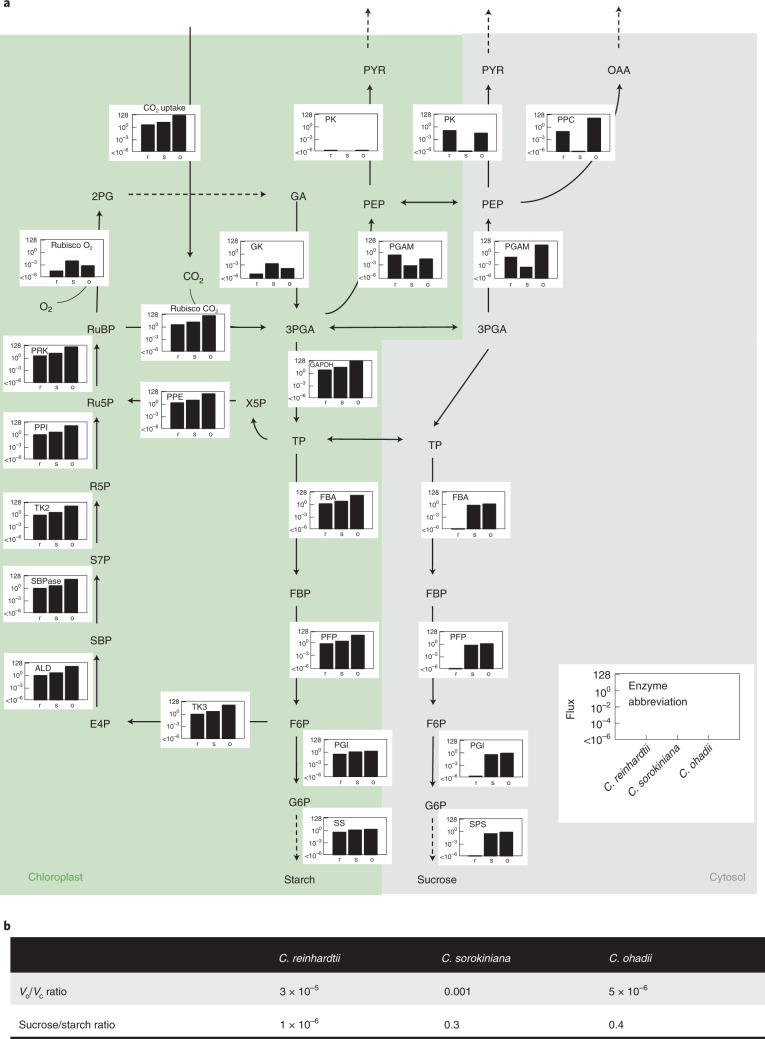
Flux estimation from INST-MFA. **a**, Map of net fluxes estimated by ^13^C INST-MFA. Bar plots next to each reaction show the absolute fluxes in mmol g^−1^ DW h^−1^ estimated under LL for *C. reinhardtii* (r), *C. sorokiniana* (s) and *C. ohadii* (o). Note that fluxes are shown on a log scale. Enzyme abbreviations follow reaction names detailed in Supplementary Table 9. Dashed arrows indicate pathways included in the model, where the full path is not shown. **b**, Comparison of estimated flux ratios. Rubisco oxygenation (*V*_o_) to carboxylation (*V*_c_) and sucrose to starch ratio. Pyr, pyruvate; OAA, oxaloacetic acid; GA, glyceric acid; E4P, erythrose 4-phosphate; X5P, xylulose 5-phosphate; TP, triose phosphate; PRK, phosphoribulokinase; PPI, pentose phosphate isomerase; TK2, transketolase 2; SBPase, sedoheptulose-bisphosphatase; ALD, aldolase; TK3, transketolase 3; SS, starch synthase; PGI, phosphoglucoisomerase; PFP, fructose-6-phosphate 1-phosphotransferase; FBA, fructose-1,6-bisphosphate aldolase; PPE, phosphopentose epimerase; GAPDH, glyceraldehyde 3-phosphate dehydrogenase; SPS, sucrose phosphate synthase; GK, glycerate kinase; PGAM, phosphoglycerate mutase; PK, pyruvate kinase; PPC, PEP carboxylase.

We also used isotopically non-stationary metabolic flux analysis (INST-MFA) to estimate intracellular fluxes (Fig. 4 and Supplementary text). CO_2_ uptake rates were much higher for *C. ohadii*, followed by *C. sorokiniana* and *C. reinhardtii*, resulting in higher CBC flux and higher fluxes to starch and sucrose in the *Chlorella* species and even higher flux through the CBC and faster export of 3PGA to the cytosol in *C. ohadii* compared with *C. sorokiniana* (Fig. 4a and Supplementary Table 9, net fluxes). *C. reinhardtii* had negligible flux through hexose-P in the cytosol (Fig. 4b). C fixation (*V*_c_/*V*_o_) was most efficient in *C. ohadii*, followed closely by *C. reinhardtii*, with the lowest efficiency in *C. sorokiniana* (Fig. 4b).

### Comparison of rates of O_2_ evolution and ^13^C incorporation

Using initial rates of ^13^C incorporation in detected metabolites we estimated minimum C assimilation rates in the algae (Table 2). These were lower than the measured rates of oxygen evolution (Table 2). This may be, in part, because some ^13^C may have moved on to metabolites that our LC–MS/MS analysis did not capture. However, previous studies in algae also reported higher rates of O_2_ evolution than of CO_2_ fixation in algae^52,53^, with a similar range to that observed by us in LL (Table 2). This can occur if a substantial part of the fixed C is converted to reduced end-products like lipids (Table 1) or Gly3P (Tables 1 and 2 and Supplementary Table 5) and glycerol or, if there is substantial consumption of nicotinamide adenine dinucleotide phosphate (NAD(P)H), in NH_4_ assimilation (Supplementary Table 5).

Using simplifying assumptions (legend to Extended Data Fig. 6) we estimated demand for Gly3P, acetyl coenzyme A (AcCoA) and NADPH in lipid biosynthesis. Fluxes to Gly3P in LL estimated in this way resembled or were only twofold above those estimated from the initial ^13^C-labelling kinetics of Gly3P (Table 2). They were tenfold higher for *C. ohadii* in EIL; this may reflect rapid movement of label through Gly3P into downstream products. Adding the estimated flux to AcCoA (Extended Data Fig. 6) to the minimum rate of C assimilation estimated from ^13^C-labelling of soluble metabolites, starch and protein (Table 2) led to 31–48% increase in the estimated rate of C assimilation in LL (now ~80%, 50% and 60% of the rate of O_2_ evolution in *C. reinhardtii*, *C. sorokiniana* and *C. ohadii*, respectively. Much of the remaining discrepancy in LL is explained by consumption of NADPH in lipid synthesis (Extended Data Fig. 6c). The corrected rate of C assimilation in *C. ohadii* under EIL was still only ~25% of the rate of O_2_ evolution. Estimated demand for NAD(P)H for lipid synthesis was 3.5-fold to sevenfold higher under EIL than LL (Extended Data Fig. 6c) but this only accounts for ~15% of the discrepancy. We may have underestimated the rate of lipid synthesis or there may be further unidentified redox group-consuming processes in *C. ohadii* under EIL.

## Discussion

Metabolic fluxes are emergent metabolic phenotypes that are generated by the levels of transcripts and the levels and properties of the encoded proteins and associated changes in metabolite levels^54–56^. We have compared the levels and labelling kinetics of >40 intermediates in central metabolism and used them, together with published data^31,32^, to compare flux patterns in three algae and model C_3_ and C_4_ plants. This comprehensive comparison of fluxomes across the plant kingdom, allows us to identify differences in flux patterns between (1) three algal species with differing growth rates, (2) *C. ohadii* in low versus very high light and (3) algae and model C_3_ or C_4_ plants.

**Fig. 5:**
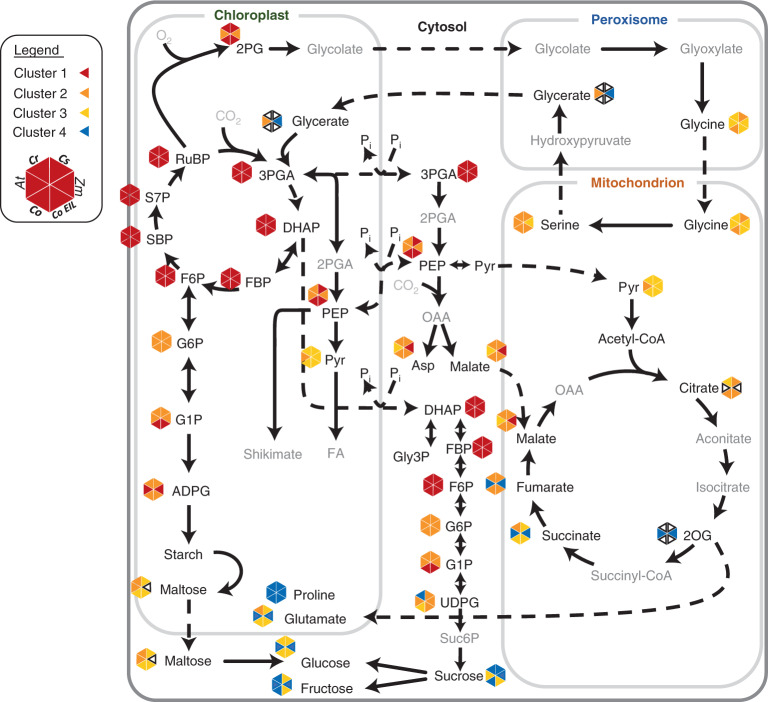
Overview of ^13^C-labelling kinetics in primary metabolism across algae, *Arabidopsis* and maize. For plants, metabolites are presented according to their clustering as performed by refs. ^31,32^. SBP in maize has been corrected on the basis of its active pool^32^. For algae, due to the separate labelling setups for the different time scales (seconds versus minutes–hours), unified clustering for the entire time series was not possible. Instead, metabolites in cluster 1 from the 0–40 s were assigned to cluster 1 and clusters 3 and 4 from the 15–300 min pulses were assigned to cluster 4. The rest of the intermediates were assigned on a case-by-case basis according to the similarity of their enrichment pattern to the published clusters for *Arabidopsis*^31^ and maize^32^, using Supplementary Tables 1, 4 and 5, to generate a qualitative comparative representation of labelling kinetics across central metabolism. For each metabolite, triangles denote clustering in each species (see legend): CO, *C. ohadii*; CS, *C. sorokiniana*; Cr, *C. reinhardtii*; At, *A. thaliana*; Zm, *Zea mays*, following the color-scheme from Figs. 1 and 2. Cluster 1 — red, cluster 2 — orange, cluster 3 — yellow and cluster 4 — blue, for empty triangles no data is available on clustering.

**Fig. 6:**
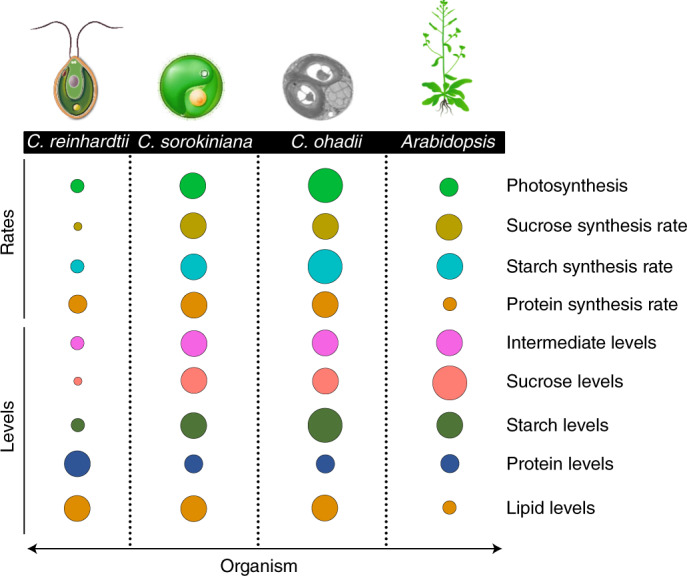
Comparative scheme illustrating differences in photosynthesis and synthesis rates and levels per unit DW of end-products (sucrose, starch and protein) and metabolic intermediates in *Arabidopsis* and three algae. Data represented are from Tables 1 and 2 and Supplementary Table 6. Photosynthesis rates are based on minimum C assimilation rates in Table 1. Due to the different light regimes (diel, continuous) between algae and *Arabidopsis*, starch synthesis rates are based on the ‘starch synthesis rate’ column in Table 2. Circle sizes correspond to interspecies relative levels/rates per parameter (row). Scale differs for each trait.

Figure 5 summarizes labelling patterns in algae and higher plants. Clustering of single labelling curves for metabolites across the entire algal time series was not possible due to the use of separate labelling setups for 0–40 s and 15–300 min. We instead associated each metabolite with the published clusters from *Arabidopsis*^31^ and maize^32^ (see legend of Fig. 5 for details). Figure 6 provides a schematic summary of estimated fluxes and contents in the various algae (extracted from Tables 1 and 2 and Supplementary Tables 3 and 6) and corresponding values for *Arabidopsis* (extracted from refs. ^31,57–59^). Compared to *Arabidopsis*, algae are characterized by high fluxes to PEP and metabolic pathways downstream of PEP, rapid protein synthesis rates (Extended Data Fig. 5) and rapid lipid synthesis (Extended Data Fig. 6). They exhibit rather diverse fluxes in sugar and starch metabolism.

The following sections discuss the differences in metabolite levels and flux patterns between algae and higher plants. These may in part reflect differing allocation and growth strategies in single-celled algae and multi-organ higher plants. In algae, C is assimilated and used for growth or stored as starch or lipid in the same cell. In higher plants, C is assimilated in source leaves and the vast majority exported to support growth in sink organs. Export occurs mainly as sucrose, although a small proportion of the fixed C is used to synthesize organic acids and amino acids. These and other potential restrictions of the datasets are discussed in the Supplementary text).

### RuBP regeneration in the CBC

The near-linear labelling kinetics in CBC intermediates in algae (Extended Data Fig. 2) demonstrated their potential as a useful model for analysis of photosynthetic metabolism. We were able to detect small differences in labelling kinetics within the CBC including faster labelling of SBP, PP and RuBP in *C. ohadii* than in *C. reinhardtii*, *C. sorokiniana*, *Arabidopsis* or maize (Supplementary Table 4 and Supplementary Fig. 3). Both labelling kinetics and INST-MFA (Fig. 4) point to *C. ohadii* having faster regeneration of RuBP, a subprocess that is often limiting for C fixation^13^.

### Coupling of lower glycolysis to the CBC

Algae differ markedly from higher plants in having higher flux from the CBC through lower glycolysis to PEP (Fig. 5 and Supplementary Table 4). Labelling of PEP is very rapid in algae, following closely the labelling of early CBC intermediates. This is especially apparent in *C. ohadii* where there is no delay in labelling of PEP compared to CBC intermediates and in *C. sorokiniana* where there is only a small delay (Fig. 2, Supplementary Fig. 3, Extended Data Fig. 2 and Supplementary Table 4). There is a more marked delay in *Arabidopsis* and especially maize (Supplementary Table 4; see Supplementary text for discussion of why this reflects the topology of C_4_ photosynthesis). Rapid flux through PEP was also apparent in longer pulses that investigated labelling patterns of end-products in algae (next section). This agreement makes it unlikely that the slow initial labelling of PEP in leaves is due to a large unlabelled pool of PEP in non-photosynthetic cells (Supplementary text).

The potential impact of this rapid flux to PEP in algae is considerable. PEP is the starting point for many important biosynthetic pathways^60^, either directly (for example, via the shikimate pathway) or via PEPC and the TCA cycle for the synthesis of many amino acids and specialized metabolites or through pyruvate and isoprenoid synthesis. Rapid lipid synthesis in algae (Table 1 and Extended Data Fig. 6) also generates high demand for PEP. Conversely, in higher plants low flux to PEP may reflect a trade-off with rapid synthesis of sucrose for export to growing sink organs^61^, in line with the high flux to sucrose in *Arabidopsis*, both in absolute terms and specially relative to the rate of C assimilation (Table 2). Indeed, on the basis of previous ^14^C-labelling studies^62,63^ flux to sucrose in *Arabidopsis* may still be underestimated by ~20%.

### Anaplerotic C investment predicts growth rate

Labelling kinetics in algae reveal substantial flow of fixed C from PEP into the TCA cycle intermediates and amino acids like pyruvate, malate, succinate, aspartate and glutamate (Figs. 3 and 5 and Supplementary Table 5). This was also captured by INST-MFA, although the predicted flux is probably largely underestimated (Fig. 4 and Supplementary text). Anaplerotic C metabolism is necessary to replace TCA cycle intermediates that are consumed by biosynthetic pathways in all life forms^64^. Whilst rate of labelling of malate in algae was comparable to that in the C_4_ species maize, other TCA intermediates like succinate and fumarate, as well as glutamate that derives from 2OG, were labelled much faster in algae than in maize (Fig. 5). Further, and importantly, the isotopomer pattern of malate and aspartate at early time points differed between algae and maize, revealing that the rapid labelling of malate in algae reflects high anaplerotic flux rather than algal C_4_-like metabolism (Supplementary text and also refs. ^65–67^). Anaplerotic flux is needed to supply C skeletons for amino acid synthesis to support the twofold to tenfold higher rate of protein synthesis in the *Chlorella* species compared with *Arabidopsis* (Supplementary Table 6). This, in turn, supports the high growth rates of these algae (Extended Data Fig. 5b). It is possible that the overall labelling kinetics of organic acids and amino acids may be affected by compartmentation, either between the cytoplasm and vacuole or between photosynthetic and non-photosynthetic cells in leaves. However, the number of metabolites affected and the supporting evidence provided by the higher rate of protein synthesis makes it very likely that the faster labelling of organic acid and amino acids in algae is at least partly due to increased anaplerotic flux.

In photosynthetic leaf cells, the enzymes that catalyse the reactions of lower glycolysis (phosphoglycerate mutase, PGM, and enolase, ENO) are largely confined to the cytosol^68^. Increased flux to PEP might be achieved by modified activity of the triose phosphate translocator (TPT) that exchanges triose phosphate, 3PGA and inorganic phosphate (Pi) between the chloroplast stroma and the cytosol^69^, increased conversion of triose phosphate to 3PGA in the cytosol, for example, by non-phosphorylating NADP-GAPDH (as recently suggested for *C. ohadii* in EIL^27^) or increased activity of cytosolic PGM and ENO. In addition, INST-MFA flux modelling and published gene expression and localization data indicate that a small part of the flux to PEP and pyruvate in algae might occur in the plastid (Fig. 4 and Supplementary Table 9, net fluxes; and see Supplementary text). Increased flux from PEP to organic acids will require operation of PEPC and (for all except oxaloacetate, malate and fumarate) mitochondrial pyruvate dehydrogenase (mPDH). Comparative sequence analysis of TPT, PGM, ENO, PEPC and mPDH revealed unique substitutions in conserved residues in algal variants compared with plant homologues (Supplementary text), with some being unique to *C. ohadii* and others shared by several *Chlorella* spp. or other algae. They included changes in key residues in regulatory or even active site domains and, in several cases, altered cysteine distribution patterns.

### Increased synthesis of galactose and Gly3P in algae

Galactose and Gly3P metabolism represent another basic difference between algae and plants (Supplementary text). Even though enrichment of galactose (Fig. 3; cluster 4 in all three algae under LL and cluster 3 in *C. ohadii* under EIL) and Gly3P (Fig. 3; cluster 4 in *C ohadii* and *C. sorokiniana* under LL, cluster 3 in *C. ohadii* under EIL and cluster 2 in *C. reinhardtii* under LL) rose slowly, their large pool sizes mean that flux of fixed C into these metabolites was substantial especially in *C. ohadii* (Table 2 and Extended Data Figs. 3 and 6), competing with fluxes to sucrose and other reducing sugars and supply of PEP and pyruvate for biosynthetic pathways (Table 1 and Extended Data Fig. 6).

### Interalgal distinctive kinetic patterns

Apart from differences between algae and higher plants, our studies uncover differences between the algal species (Fig. 6). These include lower levels of metabolic intermediates and a higher protein content in *C. reinhardtii* compared with the *Chlorella* species and the virtual absence of sucrose from *C. reinhardtii*.

*C. reinhardtii* contained much lower levels of CBC and other metabolic intermediates than the two *Chlorella* species per unit DW (Supplementary Table 3). The difference was even more marked per unit protein because *C. reinhardtii* has a higher protein content than the *Chlorella* species (Fig. 6). Generally higher levels of intermediates in the *Chlorella* species might contribute to the higher flux in the CBC, sucrose and starch synthesis, lower glycolysis and the TCA cycle compared with *C. reinhardtii* (Table 2 and Fig. 5). It might also be associated with differing osmoprotection strategies. Whereas *C. reinhardtii* accumulates large amounts of the specialized metabolite glycerol, *Chlorella* species accumulate a wider range of central metabolites like sucrose (even in non-stress conditions; Fig. 6 and Tables 1 and 2) and proline (ref. ^70^ and references therein). Whilst such differences are especially relevant under stress, they may impact on metabolic patterns in non-stress conditions. More generally, higher metabolite levels might support increased metabolic flexibility, including the ability of *C. ohadii* to cope with extreme stress^26^. Flexibility may result from more efficient enzymes but might also require higher substrate saturation, allowing higher fluxes, and partly compensating for the lower protein content in the *Chlorella* species (Table 1).

The lower protein content of the *Chlorella* species (Fig. 6 and Extended Data Fig. 5a) might contribute to their faster growth rates because protein synthesis is energetically costly. It is, however, intriguing, that despite their lower protein content, the *Chlorella* species achieve higher rates of photosynthesis than *C. reinhardtii*. It is possible that they invest a larger proportion of total protein in photosynthetic machinery than does *C. reinhardtii*, where substantial amounts of protein may be present in other cellular components like the cell wall and flagellum. The cell wall of *C. reinhardtii* contains abundant proteins and accounts for 32% of the total proteome^71^, whereas a wide but lower range of cell wall protein content has been reported in *Chlorella* species (6–27%)^72–74^. This comparison is limited by the availability of robust proteomic datasets for algae. Quantitative analysis of more algal proteomes combined with studies of the activities and kinetic properties of enzymes could improve our understanding of what limits growth rates in different algae.

Another difference between algae was the very low content and flux to sucrose in *C. reinhardtii* (Figs. 3, 4 and 5, Extended Data Fig. 3, Table 1 and Supplementary Table 5). There was also a trend to slower starch synthesis in *C. reinhardtii* than in the *Chlorella* species.

Comparing *C. ohadii* in EIL and LL revealed much faster anaplerotic flux over PEPC under EIL (Table 2) that probably contributes to rapid growth by supporting faster protein synthesis (Supplementary text). There was also increased lipid synthesis (Extended Data Fig. 6), possibly linked with faster starch turnover (Table 2 and Supplementary text).

### Prospective for plant synthetic biology

Research aiming at redesigning photosynthesis to improve plant productivity and performance is experiencing a golden-era, boosted by support from modelling and advances in computational technologies^4^. Potential avenues for improvement range from in silico manipulations to exploiting natural variation^9^. Our results highlight examples of distinct flux patterns between algae and higher plants, raising the question of what mechanisms support the faster protein and lipid synthesis in algae and whether these might provide useful resources to improve crop photosynthesis and yield (Supplementary text—potential routes for plant metabolic engineering).

## Methods

### Chemicals

Biochemicals, enzymes and other special reagents were obtained from Sigma-Aldrich, Roche and Merck. All solutions were prepared with purified deionized water (0.055 µS cm^−1^; PureLab plus and PureLab ultra, ELGA).

### Algal cultures

Cultures of *C. ohadii*, *C. sorokiniana* and *C. reinhardtii* were grown in flat glass bioreactor vessels (FMT-150, PSI), as previously described^26^. The medium in the bioreactors was HP without acetate^75^ to grow the cells photoautotrophically. Unless specified otherwise, the experiments were initiated at low cell density corresponding to optical density OD_735nm_ = 0.02 and the culture temperature was stabilized at 35.0 ± 0.3 °C for *C. ohadii* and *C. sorokiniana* or 25.0 ± 0.3 °C for *C. reinhardtii*. Irradiance levels included two regimes: 100 μmol photons m^−2^ s^−1^ (LL) and 3,000 μmol photons m^−2^ s^−1^ (EIL), as described in ref. ^27^. Air for bubbling was supplied using an air-pump at ∼1 l min^−1^. The pH and dissolved oxygen concentration were monitored in situ every 1 min. Optical densities OD_680nm_ and OD_735nm_ (which correlates with algal biomass^27^) were monitored every 1 min. All cultures were grown starting from the same inoculum density. Bioreactors were autoclaved with medium and electrodes, and axenic inoculum was added on a sterile bench, together with 0.22 µm filters on all air inlets/outlets of the system. The cultures were axenic, as validated with light microscopy (Eclipse E200, Nikon) and Luria–Bertani plating/incubation. Independent biological replicates were collected from three separate bioreactor runs for each alga or condition. Ash-free dry weight per volume for each culture was determined as in ref. ^76^.

### Gas exchange measurements

The O_2_ evolution of algal suspension withdrawn from the bioreactor was measured for 2 min at temperature corresponding to the growth conditions, in a closed temperature-controlled 0.65 cm diameter cuvette under continuous stirring (Clark type O_2_ electrode, PS2108, Passport dissolved O_2_ sensor). Light was supplied from a custom-made ring-formed LED light system; the applied intensity was made equivalent to the current growth intensity by measuring the penetrating light inside a cuvette filled with culture using an integrating sphere (Walz). Measurements were made at ambient CO_2_.

### ^13^CO_2_-labelling and sampling procedures

Two setups were used to provide inorganic ^13^C to algal liquid cultures (see Supplementary text for additional information on the requirements and design of the labelling system). For very short pulses (up to 40 s) a closed microfluidic system mixed cultures with ^13^C pre-equilibrated medium bubbled with a synthetic gas mixture. For longer incubation times (15–300 min), inorganic ^13^C was introduced via direct bubbling of the cultures with the same gas mixture. In both, 78% N_2_, 21% O_2_ and 400 ppm ^13^CO_2_ were mixed using mass flow controllers (Brooks Instruments) as described in ref. ^31^.

For rapid labelling (time points 5, 10, 20 and 40 s), a fresh HP medium (pH 6.2) was bubbled for 30 min to ensure <1% residual ^12^CO_2_ levels (Supplementary Fig. 8). In this freshwater medium, at this acidic pH, CO_2_ constitutes >70% of the total Ci species, with no large storage pool of HCO_3_ (ref. ^77^), as confirmed by the rapid drop in ^12^CO_2_ signal within <1 min of bubbling with the labelled gas mixture (Supplementary Fig. 8b). Bubbling was performed in a humidifier submerged in a temperature-controlled water bath at temperature corresponding to the growth conditions of each alga and in a separate room to avoid ^13^CO_2_ contamination of the cultures. A 20 ml syringe was washed three times and filled with the bubbled solution keeping contact with air to a minimum. Thereafter this syringe was loaded to a syringe pump (NE-1600 Multi-Channel Syringe Pump, New Era) and connected to transparent tubing (Supplementary Fig. 1a). Handling algal samples from their withdrawal from the bioreactors to quenching was planned to minimize shading of the cells due to the sensitivity of C fixation reactions to changes in light. A syringe was used to withdraw fresh algal samples from the bioreactor, loaded to the pump and connected to transparent tubing and luers. This was mixed 1:1 with ^13^C pre-equlibrated media, supplied from a second syringe (above). Cultures and fresh media were pushed through a tailor-made transparent mixer designed on the basis of ref. ^78^ (see scheme in Supplementary Fig. 1b) to transparent tubing into 70% methanol solution cooled to –70 °C as in ref. ^75^. Light was provided by an upper positioned cool-white LED array (PSI) through the entire route from sampling at the bioreactor to the quenching tube, with intensities made equivalent to the penetrating light for each culture and avoiding manual shading. Pulse duration was implemented by fitting the length of the transparent tubing downstream to the mixer to spray the culture/^13^CO_2_ solution mixture into the quenching tube at desired timings. Cooled tubes were kept at –70 °C until centrifugation (3,200*g*, 3 min, –9 °C). Thereafter, supernatant was discarded and pelleted tubes were frozen immediately in liquid nitrogen. LC–MS/MS analysis of supernatants from concentrated (15×) samples of all algae demonstrated metabolite levels of up to ~1% that of equivalent pellets. Non-labelled samples for each pulse time-series replica were generated by mixing of the same algal culture with fresh HP medium bubbled with ambient air through a humidifier at the same temperature as the ^13^C-labelling. Frozen pellets were resuspended in ice-cold methanol/chloroform (5:1, v/v). Following four cycles of freeze–thaw of resuspended cells, metabolites were extracted according to ref. ^75^.

For slow labelling, running algal cultures were bubbled with ^13^CO_2_. This was done because, in the closed microfluidic system, longer incubation times would lead to Ci depletion. The gas mixture (bubbled ∼1 l min^−1^ through a humidifier) was switched from natural air to the same synthetic ^13^C-labelled air mixture as above. Measured residual ^12^CO_2_ in the cultures was below 2% within a few minutes of pulse (Supplementary Fig. 8a) and H^12^CO_3_^−^ was rapidly equilibrated by the cells as previously reported for low CO_2_-acclimated algae^79^ and demonstrated here by the absence of a slow ^12^CO_2_ decay phase in the cultures (compare Supplementary Fig. 8a and b). Samples were withdrawn before switching (T0) and following 15, 30, 60, 120, 180 and 300 min of bubbling with ^13^C synthetic gas mixture. All samples were collected into 50 ml falcons, immediately centrifuged (3,200*g*, 3 min, –9 °C) and pellets immediately frozen in liquid nitrogen. Centrifugation was necessary because, with time, some metabolites can accumulate in the medium, so labelling patterns in the complete suspension may not reflect that in the cells (ref. ^75^). Frozen pellets were resuspended in precooled (–20 °C) MTBE solution (methanol/methyl *tert*‐butyl‐ether, 1:3, v/v) to follow a two-phase extraction approach developed by ref. ^42^. Following four cycles of freeze–thaw of resuspended pellets, metabolites, protein and starch were extracted and analysed. The lower phase, containing the polar and semipolar metabolites, was dried in a SpeedVac concentrator and stored at –80 °C for metabolite profiling. The upper MTBE phase, containing lipids, was dried and analysed for lipid content and labelling (below). The solid pellets containing the precipitated protein and starch were stored at –20 °C before further analysis (below).

### Growth and labelling of *Arabidopsis*

For *Arabidopsis*, to confirm and deepen our previous study^31^, ^13^C-labelling was repeated with a total of 15 time points (0, 5, 10, 20, 30, 45, 60, 90 s and 2, 3, 5, 10, 20, 40 and 60 min) and with three to 13 replicates per time point. ^13^CO_2_-labelling and data analysis were performed as described in ref. ^80^.

*Arabidopsis* Col-0 material was taken from ref. ^43^ for analyses of galactose and Gly3P. Briefly, plants were grown in a 1:1 mixture of soil (Stender) and vermiculite, in controlled-environment chambers with 16-h photoperiod and day/night temperatures of 22/18 °C and 150–160 µmol m^−2^ s^−1^ irradiance provided by fluorescent lights. Rosettes were harvested in the ambient growth conditions by rapid quenching in liquid nitrogen, ground and metabolites were extracted with methanol-chloroform^81^.

### Metabolite profiling

For LC–MS/MS quantification, metabolites were measured as described previously^82^. Data for 3PGA are solely based on signal peak areas obtained in extracts, as chromatograms obtained with the standards could not support absolute quantification. The obtained peaks were integrated using the Thermo Finnigan processing software package LCQuan-2.5. Since mass spectrometry detects ionized compounds separated by their mass-to-charge ratio, the mass-to-charge ratio of ^13^C-labelled compounds increases by an amount that equals the number of incorporated ^13^C atoms. Therefore, by determining the ratio of intensity of the monoisotopic ion and its isotopic ions, the level in stable isotope labelling (enrichment) was quantified. In maize, SBP has an ‘active’ pool that is involved in the CBC and is rapidly labelled, which our analysis (Supplementary Table 4) focused on, and a separate pool that labels very slowly^32^.

For GC–MS, metabolites were methoxyaminated and trimethylsilylated manually as previously described^26^. Profiling was performed using gas chromatography mass spectrometry (GC–MS) with an Agilent 6890N24 gas chromatograph (Agilent Technologies, http://www.agilent.com) with splitless injection onto a Factor Four VF-5ms capillary column, 30-m length, 0.25-mm inner diameter, 0.25-μm film thickness (Varian-Agilent Technologies), which was connected to a Pegasus III time-of-flight mass spectrometer (LECO Instrumente GmbH, https://eu.leco.com/). Retention indices were calibrated by addition of an *n*-alkane mixture (C_10_, C_12_, C_15_, C_18_, C_19_, C_22_, C_28_, C_32_, C_36_) to each sample. GC–MS chromatograms were acquired, visually controlled, baseline corrected and exported in NetCDF file format using ChromaTOF software (v.4.22; LECO). TagFinder software v.1.0 (ref. ^83^) was used for GC–MS data processing into a standardized numerical data matrix and compound identification. Mass features were identified by mass spectral and retention time index matching to the reference collection of the Golm metabolome database (GMD, http://gmd.mpimpgolm.mpg.de/)^84^. Requirements for the manually supervised analyte identification were the presence of at least three specific mass fragments per mass feature and a retention index deviation <1.0%. Metabolite isotopologues were evaluated for uniqueness by correlation analysis, using the sum of all detected isotopologues per mass fragment and comparing among fragments or molecular ions from the same analyte. The sum of isotopologues were used in both labelled and non-labelled conditions for quantification, while isotopologue distributions were used for stable isotope enrichment analysis. Absolute quantification was performed, using authenticated commercially available reference substances and co-analysed calibration graphs. Enrichment analysis was restricted to mass fragments with known atomar composition. Expected atomar composition and natural enrichment were checked by non-labelled samples, for example those used to obtain calibration curves. After correction for natural occurring isotopes, experimental enrichment of approximately zero was obtained. Laboratory and reagent contaminations were evaluated and removed by non-sample control experiments.

Both LC–MS/MS and GC–MS matrices were subsequently processed using the Corrector software tool (https://www.mpimp-golm.mpg.de/19405/Corrector_package_V1_91.zip). Metabolite quantification from both platforms was performed on the basis of calibration curves obtained with authentic standards whose matrices were also corrected. Relative isotopomer abundance (*m* + *n*) for each metabolite is calculated as in ref. ^31^. Total amounts of PEP were determined by using freshly prepared extracts in trichloroacetic acid. Levels were determined enzymatically with Shimadzu UV-2600i spectrophotometer (Shimadzu) based on ref. ^85^.

GC–MS chromatograms from the algae and *Arabidopsis* overlaid on a Gly3P standard are provided in Supplementary Fig. 8 for illustration. As aldose sugars show similar mass features and retention times in GC–MS, we verified our analyses by overlaying plant and algal chromatograms on those of eight hexoaldose standards (Supplementary Fig. 9).

### Starch quantification and enrichment

Starch in pellets was analysed as in ref. ^57^. Briefly, following neutralization, starch was digested to glucose with α-amylase and α-amyloglucosidase. Thereafter, glucose was assayed enzymatically for total starch content or converted to G6P by incubation with hexokinase and excess ATP, and ^13^C in G6P was measured by LC–MS/MS^86^.

### Protein quantification and enrichment

Total protein was extracted from the insoluble pellets for the pulse times 15–300 min after solubilization with 0.1 M NaOH at 95 °C for 60 min and measured using the dye-binding assay^87^. To account for full extraction and quantification of total protein, we followed the levels of protein after varying freeze–thaw iterations and incubation time with 0.1 M NaOH. In all algae and conditions, protein levels did not increase beyond three freeze–thaw cycles or 30 min of incubation.

To estimate synthesis rates, total soluble protein was isolated, hydrolysed and analysed by GC–MS to determine enrichment in protein-bound amino acids. Briefly, ~100 µg per sample was precipitated and washed and then chemically hydrolysed with 6 M hydrochloric acid at 100 °C for 24 h. Hydrolysate was dried and derivatized for GC–MS analysis. The resulting chromatograms were processed as described above for fragmental isotope enrichment of identified amino acids. Enrichment in amino acids in protein was analysed and quantification of protein synthesis rates from these enrichment data was corrected for incomplete labelling of the free amino acid as previously described^58^.

### Lipid quantification and analysis

Measurement of lipids was performed as described by ref. ^88^. In brief, dried MTBE phases were resuspended in a volume of 200 μl of a mixture of acetonitrile:isopropanol (7:3) (Biosolve) and measured using Waters Acquity ultra-performance liquid chromatography system (Waters, http://www.waters.com) coupled with Fourier transform mass spectrometry (UPLC–FT-MS) in positive and negative ionization modes. Analysis and processing of mass spectrometry data was done with REFINER MS 10.0 (GeneData, http://www.genedata.com) and comprised peak detection, RT alignment and a chemical noise removal. Derived mass features characterized by specific peak ID, *m/z* values, retention time and intensity were further processed using custom R scripts. Before annotation of metabolic features using in-house lipid database, isotopic peaks were removed from the MS data. Annotated lipids were confirmed by manual investigation of the chromatograms using Xcalibur (v.3.0, Thermo-Fisher). Peak intensities were day-normalized and sample median-normalized and subsequently log_2_-transformed. For lipid labelling, we restricted our analysis to m0 decay of major labelled lipids between 0 and 300 min due to their high molar mass and the very complicated spectra with partly overlapping envelopes of ^13^C-labelled lipids.

### Data analysis and statistics

Data processing and statistical analysis were performed in R (ref. ^89^). MDS was performed for the four algae and conditions with stats R package v.3.6.0 using Euclidean distance^90^. The input Euclidean distance matrix was calculated on a row-wise concatenated enrichment–time-series matrix of all algae and conditions. Clustering has been performed separately for each alga using *k*-means^91^ and the number of clusters has been estimated using GAP statistics for *k* between 2 and 20 in 100 bootstrap samples^92^ in Cluster R package v.2.0.8 (ref. ^93^). ANOVA were performed using the car R package v.3.0.9. Minimum estimates of fluxes to all organic and amino acid quantified by GC–MS analysis were performed by summing the slopes over time of level × enrichment products for pyruvate, citrate, glutamate, proline and glycine, provided in Supplementary Tables 3 and 5, respectively.

### INST-MFA

Flux estimation by INST-MFA, statistical analysis, as well as confidence interval calculation were performed using implementations provided along the INCA software package^94^. INCA relies on the elementary metabolite unit decomposition^95^ to estimate flux values that provide an optimal fit of simulated and measured metabolite labelling patterns of measured metabolites as well as statistical and sensitivity analysis of the optimal solution. The isotopomer model of ref. ^37^, which includes reactions of the CBC, photorespiration, starch and sucrose synthesis, TCA cycle as well as amino acid synthesis, was adapted by removing the biomass reactions simulating transport into phloem and adding reactions from 3PGA to PEP. All model reactions and atom transitions are listed in Supplementary Table 9 (reactions). To estimate fluxes in the modelled metabolic network we used mass isotopomer distributions (MIDs) of 17 metabolites (for a list of considered reactions see Supplementary Table 9, used MIDs) over four time points (5, 10, 20 and 40 s). We used the same model across all species, since there are no differences in the stoichiometry of the key pathways in central carbon metabolism between these species; in addition, for fair comparison, we also used the same number of metabolites treated in the same way across the species (Methods). We performed flux estimation in 50 repetitions from different random initial values to obtain best-fit estimates. This gave statistically acceptable fits to measured labelling patterns for *C. reinhardtii* and *C. sorokiniana* (*χ*^2^ = 180.3 and *χ*^2^ = 262.5, respectively, 239 d.f.) and following exclusion of the MIDs of 2OG and glutamate and PEP at 40 s (Supplementary Table 9, used MIDs), for *C. ohadii* in LL (*χ*^2^ = 219.3, 187 d.f.). The agreement between estimated and measured starch and sucrose synthesis rates (Table 1) contributes to the *χ*^2^ for all algae. In line with ref. ^37^, metabolite pools were treated as parameters to be fitted and dilution was allowed to model effects of unlabelled subpools. The 95% confidence intervals were computed for all estimated parameters by evaluating the sensitivity of the sum-of-squared residuals to parameter variations^96^. To obtain statistically acceptable fits, the minimum standard deviation of MIDs was set to 0.001 and the maximum standard deviation over all mass fractions for a given metabolite and time point were considered as error.

### Reporting Summary

Further information on research design is available in the Nature Research Reporting Summary linked to this article.

## Supplementary information

Supplementary Information Supplementary text, Tables 3, 6 and 7 and Figs. 1–11.

Reporting Summary

Supplementary Tables Supplementary Tables 1, 2, 4, 5 and 8.

Supplementary Table 9 INST- MFA parameters.

Supplementary Data 1 Enolase sequence alignment from algae and plants.

Supplementary Data 2 TPT sequence alignment from algae and plants.

Supplementary Data 3 PEPC1 sequence alignment from algae and plants.

Supplementary Data 4 PEPC4 sequence alignment from algae and plants.

Supplementary Data 5 PDH_E1 sequence alignment from algae and plants.

Supplementary Data 6 PDH kinase mitochondrial sequence alignment from algae and plants.

Supplementary Data 7 PDH kinase sequence alignment from algae and plants.

## Data Availability

All raw metabolite profiling data are provided in the Supplementary Information tables. Reference collection of metabolites from the GMD are available on http://gmd.mpimp-golm.mpg.de/. Sequence data for *C. ohadii* are available on NCBI (accession PRJNA573576) and in Supplementary Data 1–7.

## Code availability

The entire INCA implementation, including data, metabolic model and final simulations, can be found on GitHub (https://github.com/ankueken/INST-MFA_algae) in an INCA readable format.
