# Supplementary material for: Carbon flux through photosynthesis and central carbon metabolism show distinct patterns between algae, C3 and C4 plants
Source: Nat Plants. 2021 Dec 23;8(1):78–91. doi: 10.1038/s41477-021-01042-5 (PMC8786664; doi:10.1038/s41477-021-01042-5)
Supplement: Supplementary file 1 — Supplementary text, Tables 3, 6 and 7 and Figs. 1–11. [file 41477_2021_1042_MOESM1_ESM.pdf]

nature portfolio

https://doi.org/10.1038/s41477-021-01042-5

Supplementary information

Carbon flux through photosynthesis and central carbon metabolism show distinct patterns between algae, C_3 and C_4 plants

In the format provided by the authors and unedited

nature portfolio

https://doi.org/10.1038/s41477-021-01042-5

Supplementary information

Carbon flux through photosynthesis and central carbon metabolism show distinct patterns between algae, C_3 and C_4 plants

In the format provided by the authors and unedited

Supplementary Text

Design of algal ^{13}C labeling system

It is challenging to supply inorganic ^{13}C to algae in aqueous systems. We used two set-ups; a closed system in which pre-equilibrated inorganic ^{13}C was introduced which allowed a precise clear start but was restricted to very short pulses (up to 40 s) due to draw-down of the inorganic ^{13}C, and an open system with bubbling of $^{13}CO_2$ which allowed long pulses but did not facilitate a precise start of labeling because time is needed for equilibration of inorganic C species. Labeling and harvesting were designed to meet three requirements. First, very short precise pulses of label followed by instantaneous quenching in prevailing conditions are necessary to analyze CBC flux due to very short turnover times of CBC metabolites [1]. Second, after introduction of ^{13}C-labeled inorganic carbon (Ci), it must rapidly equilibrate between the Ci species (CO_2, HCO_3^{1-}, CO_3^{2-}, H_2CO_3). Third, Ci levels must be held within a tight range to maintain steady state metabolic conditions. To address these partly conflicting requirements, we designed a transparent microfluidic mixer (Figures 1 and Extended Data figure 1). Samples were withdrawn from a running culture under a light table and mixed 1:1 with labeled growth medium that had been pre-bubbled with a synthetic labeled air mixture prepared from pure N_2, O_2, and $^{13}CO_2$ for long enough to replace >95% of CO_2 and HCO_3^- with their ^{13}C-labeled forms (see Methods). CO_2 is the major Ci species (>70%) at the pH of the medium. Due to the 1:1 mixing of the labeled and unlabeled growth medium, the final enrichment in $^{13}CO_2$ and $^{13}HCO_3^-$ was half that of the original $^{13}CO_2$ mix. After mixing, the suspension was circulated in the transparent mixer system under the same light conditions as in the running cultures for 5, 10, 20 and 40 s before injection under the same light conditions into 70% methanol (-70°C). Samples collected in the 0-40 s time-scale were analyzed by LC-MS/MS [2] to focus on phosphorylated intermediates in the CBC and early steps of end-product synthesis pathways (Table S1).

In the closed microfluidic system, longer incubation times would lead to Ci depletion, thus, for longer labeling periods, we used direct labeling of running cultures. The gas mixture bubbled through the culture was switched from natural air to the same synthetic labeled air mixture as above, followed by sampling. Residual $^{12}CO_2$ in the cultures fell below 2% within a few minutes of pulse (Figure S10a, and Methods). Centrifugation was necessary because with time some metabolites can accumulate in the medium, so labeling patterns in the complete suspension may not reflect that in the cells (see [3]). Due to the different setups and a gap in the time-series, fluxes had to be estimated from one or the other of the resulting datasets. Some of the flux values obtained from the short-term dataset are underestimates because ^{13}C is spread between many downstream metabolites and products that cannot be reliably detected after a very short pulse; this particularly affects flux through PEP. Furthermore, in *C. ohadii* in EIL where fluxes were especially high, the labeling kinetics of metabolites were fast and similar to each other limiting their

usefulness in flux estimation. A future engineering challenge is to design a solution for continuous delivery of pre-equilibrated inorganic ^{13}C, allowing precisely-timed short but also longer pulses whilst maintaining inorganic C concentrations constant.

Labeling of starch and sucrose synthesis intermediates

Several differences in enrichment in intermediates of starch and sucrose synthesis were found between algae. G1P (assigned to Cluster 1 in *C. ohadii* and Cluster 2 in the other algae, Figure 2 and Extended Data figure 2) is an intermediate of starch synthesis and sucrose synthesis. In regression to DHAP, G1P had a slope of 0.83 *in C. ohadii* compared with 0.54 and 0.69 in *C. reinhardtii* and *C. sorokiniana* in LL, respectively (Figure S3, Tables S1 and S4), and an even steeper slope in *C. ohadii* in EIL (0.90) where pool size was also larger than in LL (Table S3, Figure S3). A similar picture emerged for dedicated downstream intermediates, which provide more specific information about the rate of end-product synthesis. ADPG, the first dedicated intermediate in starch synthesis, labeled faster in *C. ohadii* (assigned to cluster 1, like G1P) than other algae (assigned to cluster 2, like G1P). Enrichment of UDPG, which is an intermediate in sucrose synthesis, rose much faster in the *Chlorella*s (cluster 2 or 3) than *C. reinhardtii* (cluster 4) (Extended Data figure 2, Tables S1 and S4). This reflects much slower sucrose synthesis in *C. reinhardtii* (see below). Compared to algae, ADPG, G6P and G1P labeled more slowly in maize (slope of 0.75, 0.33 and 0.35, respectively) and Arabidopsis (slope of 0.34, 0.21 and 0.10).

Enrichment of G6P lagged behind that of F6P in all algae and behind G1P except in *C. reinhardtii* (Figure 2, Tables S1 and S4). This resembles Arabidopsis and maize (Tables S1 and S4). In photosynthetic eukaryotes, the overall labeling kinetics of hexose phosphates are complicated by subcellular compartmentation; starch is synthesized in the chloroplast, and DHAP is exported to the cytosol where it is converted via hexose phosphates and UDP-glucose to sucrose [4]. In higher plants, slow labeling of G6P is attributed to two factors. First, G6P is low in the chloroplast and is mostly located in the cytosol [5] because the plastid phosphoglucoisomerase (pPGI) reaction is removed from equilibrium in the light [5] [6] due to inhibition by E4P [21]. Second, flux to sucrose is much slower than flux in the CBC [7]. The slow labeling of G6P compared to F6P in algae (Extended Data figure 2, Tables S1 and S4) is consistent with a similar scenario (see below for modelling of fluxes in the cytosol and plastid).

Labeling kinetics of starch reveal simultaneous synthesis and degradation

In our experiments, we grew the algae under continuous light to minimize diurnal responses, and focus on photosynthetic metabolism in steady state conditions. Higher plants simultaneously synthesize and degrade starch in sustained illumination ([8] and references therein). Maltose is an early intermediate in the pathway of starch breakdown, and glucose is also formed during starch degradation [9]. Labeling patterns

of maltose and glucose in algae implied that some of these metabolite pools were derived from degradation of preformed and hence unlabeled starch. To examine this scenario further we estimated how quickly ^{12}C was released by degradation of starch (see legend of Table 2) and compared this with the rate of $^{13}CO_2$ fixation. ^{12}C supply from starch degradation was equivalent to 6-19% of calculated photosynthetically fixed ^{13}C (Table 2).

In our conditions of continuous light, algae simultaneously synthesized and degraded starch (Table 2 and Extended Data figure 4). This led to release of partially labeled maltose and glucose (Table S5 and Figures 5 and Extended Data figure 3), recycling ^{12}C at rates that were high enough to depress enrichment in central intermediates (Table 2). Based on these rates of starch degradation, and the very low residual $^{12}CO_2$ in the cultures (Figure S10), we conclude that substantial amounts of ^{12}C are recycled from pre-existing starch in algae. This ^{12}C will enter the hexose-phosphate pools in the cytosol, and possibly in the chloroplast, contributing to the incomplete enrichment of central phosphorylated metabolites in algae.

In higher plants, contrary to the idea that starch is not degraded in the light, recent studies have emphasized that the rate of degradation rises with time in the light [9, 10] and that starch degradation recycles unlabeled C leading to incomplete enrichment of intermediates in $^{13}CO_2$ labeling studies [11, 12]. Thus, starch turnover may respond to continuous light in a similar manner in algae and higher plants.

Protein synthesis rates

To estimate synthesis rates, total soluble protein was isolated, hydrolyzed and analyzed by GC-MS to determine enrichment in protein-bound amino acids. Quantitative interpretation is complicated by incomplete labeling of the free amino acids, which are the precursors for protein synthesis [13]. This explains why enrichment rose faster for some protein-bound amino acids than for others (Table S8). The free pools of aspartate and glycine could be reliably detected in all algae and exhibited quite high enrichment from 120 min onwards (Table S5). These values were used to correct ^{13}C enrichment in protein-bound aspartate and glycine and estimate absolute protein synthesis rates (PSR, % of protein synthesized hr^{-1}) between 120-300 min (Extended Data figure 5 and Table S6). $PSR_{aspatate}$ tended to be lower than $PSR_{glycine}$, especially in *C. reinhardtii* (Table S6).

We compared algal PSR with published values for Arabidopsis (data are not available for maize). PSR calculated from aspartate and glycine labeling kinetics in the *Chlorella*s were 2.3 to 3.9-fold higher than estimates using these amino acids in Arabidopsis (1.2 and 2.0 % hr^{-1}, Table S6, calculated from data in [13]). The preferred amino acid for estimation of PSR in Arabidopsis was alanine due to fast labeling of its free pool. The published value (1.95% hr^{-1}, [13]) confirms >2-fold higher PSR in *Chlorella*s than Arabidopsis.

Comparison of intracellular fluxes in algae isotopically nonstationary metabolic flux analysis (INST-MFA)

To estimate intracellular fluxes, we used isotopically nonstationary metabolic flux analysis (INST-MFA) implemented in the INCA software package [14]. We adapted the isotopomer model of [15] by removing the biomass reactions simulating transport into phloem and adding reactions from 3PGA to PEP (see Methods). Ordering of fluxes was similar in *C. reinhardtii* and *C. sorokiniana* (Spearman correlation 0.76, P value = $2e^{-10}$); however, the uptake rate for CO_2 (see Table S9-Net fluxes, model reaction T_CO2 importing CO_2 from the external unbalanced pool CO_2.t) was about 3-fold higher in the latter (0.6 and 2 mmol g^{-1} DW h^{-1}, respectively) resulting in higher CBC flux and higher flux to starch (0.07 and 0.22 mmol g^{-1} DW h^{-1}, respectively) and sucrose (less than $1e^{-6}$ and 0.06 mmol g^{-1} DW h^{-1}, respectively). CO_2 uptake rates were much higher for *C. ohadii* in LL (68 mmol g^{-1} DW h^{-1}), resulting in even higher flux through the CBC (Figure 4 and Table S9-Net fluxes) and faster export of 3PGA to the cytosol. Cytosolic 3PGA is mainly converted to PEP that is metabolized in the anaplerotic reaction to the TCA cycle (i.e. PEPC). Fluxes to starch (0.3 mmol g^{-1} DW h^{-1}) and sucrose (0.13 mmol g^{-1} DW h^{-1}) were also largest in *C. ohadii*. Overall, the ratio between flux in the cytosol to sucrose and in the plastid to starch were much higher in the *Chlorella*s (Figure 4b) than *C. reinhardtii*, which had negligible flux through hexose-P in the cytosol. Rubisco oxygenation was very slow compared to carboxylation in all algae (Figure 4b). C fixation was most efficient in *C. ohadii* ($V_0/V_c = 5e^{-6}$), followed closely by *C. reinhardtii* ($V_0/V_c = 1e^{-5}$), with the lowest efficiency in *C. sorokiniana* ($V_0/V_c = 0.001$).

We also computed 95% confidence intervals for all estimated parameters by evaluating the sensitivity of the sum-of-squared residuals to parameter variations [16]. The above statements about optimal fits also held for the lower confidence bounds, corroborating the identified trends. However, for most reactions, upper confidence bounds could not be determined with INCA and different settings, preventing statistical analysis of flux ranges between algae (Table S9-Net fluxes).

We were unable to obtain statistically acceptable fits for *C. ohadii* under EIL, probably because the extremely fast labeling in EIL means that differences between pools may be insufficient to resolve fluxes. Statistical fits were only possible if the minimum standard deviation of MIDs was set to 0.001 and the maximum standard deviation over all mass fractions for a given metabolite and time point was considered as error of measurements. Relaxation of the error of measurement to allow statistical fits is a strategy also applied in other studies in the field [15, 17]. Such enforcement can affect the ability to obtain optimal flux fits as well as confidence intervals. Though other models of measurement error (e.g. maximum over MIDs and time points for measured metabolites) might help, the extremely fast labeling in EIL means that differences in labeling between pools may be insufficient to resolve fluxes.

Galactose and Gly3P in algae

Galactose is an important component of the algal cell wall [18, 19] and an intermediate in algal ascorbic acid synthesis [20, 21]. In all three algae galactose levels were 5- to 30-fold higher than glucose levels (Table S3). The levels in *C. reinhardtii* resemble those reported previously [22], and earlier studies with other *Chlorella* sp. reported galactose was more abundant than glucose [23]. Even though enrichment of galactose rose slowly (Figure 3, Cluster 4 in all three algae in LL and cluster 3 in *C. ohadii* in EIL) the large pool sizes mean that flux of fixed C into galactose was substantial, especially in *C. ohadii* under EIL (Table 2, Extended Data figure 3). In algae, flux to galactose competes with flux to sucrose and other reducing sugars. This contrasts with Arabidopsis where sucrose was high, glucose was the main reducing sugar and galactose was 3-6-fold lower than in algae (Table 1).

Gly3P provides the C_3 backbone for synthesis of TAG and most membrane lipids, and is the precursor for glycerol accumulation [24, 25]. Gly3P was among the most abundant metabolites in the three algae (Table S3), with levels 1-2 orders higher than reported for Arabidopsis [26]. Even though Gly3P enrichment rose quite slowly (Figure 3, Cluster 4 in *C ohadii* and *C. sorokiniana* in LL, Cluster 3 in *C. ohadii* in EIL and Cluster 2 in *C. reinhardtii* in LL) its large pool size (Table S3) means that flux to Gly3P was substantial, especially in *C. ohadii* (Table 2, Extended Data figure 6). The high lipid content (Table 1) and synthesis rate (Extended Data figures 6a and 6b) in algae will create a large demand for Gly3P (Extended Data figure 6c). Indeed, the rapid labeling of Gly3P in the 0-40 s pulse could be fully explained by the demand created by lipid synthesis (see Extended Data figure 6). Flux to Gly3P will compete with sugar synthesis, supply of PEP for anaplerotic biosynthesis and provision of pyruvate for AcCoA synthesis (Extended Data figure 6c). This will be especially the case in *C. ohadii* in EIL.

It remains an open question how flow of C to Gly3P and FA is coordinated when there is rapid lipid synthesis, as in *C. ohadii* in EIL. Incidentally, rapid lipid synthesis and the associated demand for NADPH may impact on photosynthesis. Increased demand for NADPH relative to ATP has potentially far-reaching consequences for the relation between linear and cyclic electron transport, poising of the NADPH/NADP+ and ATP/ADP ratios and CBC operation.

Limitations of inter-species comparative analysis

Comparison of labeling kinetics between algae and leaves is not trivial. A pre-condition for comparison at short labeling times was our experimental set-up that guaranteed immediate and equivalent enrichment of all inorganic carbon species (Ci, mainly CO_2 and bicarbonate) in labeling experiments with algae. However, further factors can also complicate this comparison.

One is the presence of an internal inorganic C pool (mainly CO_2 and HCO_3^- in algal cells, and CO_2 in the intercellular air spaces and CO_2 and HCO_3^- in the cells of leaves) whose size may differ between algae and leaves leading to differences in the initial rate of ^{13}C incorporation into metabolites. Based on published data, the internal inorganic C pool in algae operating a CCM is over ten times larger than in C_3 leaves, and is of a similar size to that in the bundle sheath cells of C_4 leaves [2, 27, 28]. Hence, this would even tend to slow down label incorporation in algae compared to leaves, especially C_3 leaves.

Another potential complication is the presence in leaves of non-photosynthetic cells with metabolite pools that may slow the rise in enrichment of metabolites when averaged over the entire leaf. Slowing down may also occur if the pool size of metabolites in the vacuole is larger in higher plants than algae.

Second, the data are from total culture or leaf extracts. Species variation in sub-cellular (and in plants, cell type) compartmentation, and the contribution of non-photosynthetic cells in plants, may underlie some of the inter-species differences in metabolite levels and overall labeling patterns. This problem can be in part circumvented by focusing on the 'active' metabolite pool that is rapidly labeled by newly fixed C, and can in this way be distinguished from secondary or 'inactive' pools in the vacuole or in non-photosynthetic cells [7, 29]. It was addressed by relating labeling kinetics of metabolites to those of DHAP and RuBP; the former is an early product of C fixation and the latter is restricted to the CBC and therefore provides a baseline for labeling kinetics in photosynthetic cells. We also performed INST-MFA to provide predictions about plastidic and cytosolic flux through hexose phosphates and to PEP. We also considered the possible impact of compartmentation on the labeling kinetics of three different sets of metabolites. I) CBC intermediates. Such effects are very unlikely to affect the labeling kinetics of dedicated CBC intermediates as these are almost certainly restricted to the chloroplast stroma pool (plus any cytosolic pool like 3PGA and DHAP that are in close isotopic equilibrium with stromal pools). Thus. we conclude that the faster labelling kinetics of CBC intermediates is indeed a result of higher flux in algae. II) Intermediates of starch and sucrose synthesis. Any such effect for intermediates of sucrose synthesis is also likely be small and, as discussed above, the labeling kinetics observed in algae are consistent with a rather similar sub-cellular compartmentation of starch and sucrose synthesis as in higher plant leaves (see section 'Labeling of starch and sucrose synthesis intermediates'). III) PEP, whose enrichment increases more quickly in algae than in Arabidopsis or maize. This faster rise in algae was seen not only for enrichment of PEP, but also for enrichment of PEP normalized on DHAP. The faster rise in PEP enrichment in algae than in higher plant leaves is therefore extremely unlikely to be explained by the presence of a substantial unlabeled pool of PEP in non-photosynthetic cells of leaves. This would require that these unspecified non-photosynthetic cells contain a substantial pool of PEP, but not of DHAP or, by implications, other glycolytic intermediates.

Third, the datasets for algae were obtained in continuous light, whereas for higher plants they were obtained in light-dark cycles when part of the assimilated C is stored transiently as foliar starch to support sucrose export, respiration and growth at night. Starch turnover is a highly regulated process [9, 10, 30] and some of the differences in fluxes to starch and sugars between algae and higher plants might reflect the contrasting response in continuous light and light-dark cycles.

Fourth, our estimates of flux to lipids are not as comprehensive as other aspects of our analyses. Nevertheless, the estimates reached by bulk analysis (Extended Data figure 6c) received strong qualitative support from ^{13}C labelling kinetics (Extended Data figure 6a). Further, at least in LL, the estimated demand for Gly3P for lipid biosynthesis (Extended Data figure 6c) was close to or only two-fold above flux to Gly3P estimated from initial labelling kinetics (Table 2).

Slow labeling of PEP and the isotopomer pattern of malate and aspartate at short labeling times in maize reflect the topology of C_4 photosynthesis

In some cases, different labeling patterns reflect distinct topologies of photosynthetic-metabolism networks between different plant and algae, as in the case of malate and aspartate isotopomer patterns. In algae, not only m+1 but also multiply-labeled isotopomers of these intermediates appeared early in the labeling kinetic, as expected if there is rapid flux of C from the CBC via PEPC into malate and other organic acids. This contrasts with C_4 photosynthesis, where only the m+1 isotopomer of malate and aspartate is found at early time points.

This is because C_4 photosynthesis involves incorporation by PEP carboxylase of $H^{13}CO_3$ at the C_4 position of 4-carbon metabolites like malate in the mesophyll cells, followed by a rapid intercellular shuttle in which 4-carbon metabolites move to bundle sheath cells where they are decarboxylated, with 3-carbon metabolites returning to the mesophyll to regenerate PEP (see Introduction, [31, 32]). At early labeling times, this returning flux is not labeled, resulting in PEP remaining largely unlabeled (indeed, even less labelled than in Arabidopsis) and labeling of malate and aspartate being restricted to the C-4 position [29]. Thus, rapid labeling of malate in algae likely reflects high anaplerotic flux rather than algal C_4-like metabolism [33-35].

Differences between *C. ohadii* in LL and EIL

Comparing *C. ohadii* in EIL and LL, the labeling kinetics of malate and aspartate (at 5-40 s) were much faster in EIL (Tables 2, S1, S4 and S5, note that flux over PEPC will be strongly underestimated in *C. ohadii* in EIL, see Results - Comparison of estimated fluxes to end-products). This presumably contributes to the faster growth under EIL, for example by accelerating amino acid synthesis to support

faster protein synthesis (Table S6). It might also contribute via malic enzyme to delivery of pyruvate for FA synthesis. This increase in anaplerotic flux and the unique sequence characteristics of PEPC in *Chlorella*s (see below) point to the importance of studying its regulation under EIL.

Somewhat surprisingly, *C. ohadii* contained less starch in EIL than in LL. This was mainly due to starch degradation being 70% faster in EIL than LL (Table 2 and Extended Data figure 4d). This is in line with higher maltose enrichment in EIL (Table S5 and Extended Data figure 3) despite an unchanged pool size (Figure S11). Lipid levels, on the other hand, were higher in *C. ohadii* in EIL than LL (Table 1, Extended Data figure 6 and [36]; up to 3-4 orders higher for some TAG species [37]. A large increase in flux to lipids in EIL is consistent with a 2.7-3.8-fold higher transcript abundance for plastid PDH in *C. ohadii* in EIL, but only 1.4-1.5-fold for mPDH (Table S7), compared with LL. A switch from starch to lipid accumulation provides a substantial sink for reducing equivalents in EIL (Extended Data figure 6). Future work combining rapid labeling (Extended Data figure 1) with recently developed methods for quantification of nascent acyl chains attached to acyl carrier proteins [38] may allow more precise analysis of flux to lipids in parallel with the analysis of other fluxes.

Potential routes for plant metabolic engineering

In some cases, algae exhibit higher fluxes than plants in a highly conserved pathway. One is faster labeling of late CBC intermediates in *C. ohadii* compared to other algae, Arabidopsis or maize (Figure S3 and Table S4). With rising atmospheric [CO_2], regeneration of RuBP is expected to become progressively limiting for photosynthesis [39]. Higher fluxes through late CBC in *C. ohadii* may reflect altered kinetic characteristics, regulation or unique molecular organization of enzymes involved in RuBP regeneration, an aspect of which we still have limited understanding [40]. We previously demonstrated distinct redox-dependent behavior of *C. ohadii* CP12 protein compared to known algal and higher-plant CP12 [37], a regulatory protein that organizes CBC enzymes in multi-protein complexes. In addition, the unique patterns of cysteine distribution in several metabolic enzymes in *Chlorella* species (see below and [37]) raises the possibility that altered redox-biology may contribute to higher fluxes through CBC. Exploiting one or more algal CBC enzymes would be a relatively simple and promising path to improve photosynthesis in plants, resting on the previous examples where overexpression of single CBC enzymes positively affected crop biomass (for a detailed summary, see [41]).

Another strategy would be to build on the faster glycolytic flux to PEP in algae. PEP and pyruvate are central precursors in energy metabolism and important C skeletons for biosynthesis. Our studies of algae indicate that higher flux to PEP might be achieved by altered functionality of several proteins including TPT, non-phosphorylating GAPDH, and the dedicated enzymes PGM and Eno (see below).

Given the substantial labeling in PEP in early time points even in terrestrial plants (Table S1), this approach will not necessarily require a large deviation from existing allocation strategies in crop plants. Engineering to increase flux to PEP might support existing efforts to increase TAG content and further use of leaves as bioenergy feedstocks [42]. Increasing lipid synthesis in leaves might also be aided by a deeper understanding of regulatory mechanisms that promote Gly3P synthesis in algae. Interestingly, rapid TAG accumulation in *C. ohadii* in EIL was also associated with increased turnover and lower levels of starch.

Another goal would be to increase anaplerotic flux from PEP into the TCA. In the single-celled algal system, anaplerotic flux was largely predictive of growth rates. In leaves, increased anaplerotic flux might serve to increase biosynthesis of amino acids and other products that derive from the TCA. It might be achieved by manipulating mPDH and PEPC; the latter might build not only on growing knowledge of PEPC forms from algae (see below) as well as plants. This strategy is ambitious as anaplerotic flux may already be optimized in leaf photosynthetic cells, or buffered by interconversion of C metabolites and amino acids in bundle sheath in the leaf vasculature [43, 44] or elsewhere in the plant. Indeed, the higher anaplerotic flux in algae may represent the difference between photosynthesis in a growing cell and in source leaves which, rather than supplying all the amino acids needed by growing tissues, export much of their photosynthate as sucrose to be later converted into amino acids. Insofar, accelerating anaplerotic flux in flux might disturb the sink-source balance.

CCM control on photorespiratory C flow

The efficiency of CCM can be assessed by asking to what extent it concentrates CO_2 at the carboxylation site, and minimizes RuBisCO oxygenase activity and the resulting energy-costly process of photorespiration [45]. We did not observe clear differences in the labeling kinetics of 2PG or the ratio of 2PG to DHAP labeling between all three algae, C_3 and C_4 plants (Tables S1 and S4), nor in the labeling kinetics of glycine (Table S5). The similar ratio of 2PG/DHAP labeling is in line with a very narrow range found for the carboxylation/oxygenation specificity of RuBisCO in a survey of kinetic data of this enzyme from ~300 organism [46]. However, the absolute levels of 2PG (Figure S4) were lower in algae than in C_3 or C_4 plants. The increased levels of 2PG in *C. ohadii* at EIL may be attributed either to leaky operation of the CCM under prolonged high light [37] or to the development of Ci limitation in the mixing chamber, as previously reported in measurement of these cells in metabolic cells [36] [37], or both.

Recent efforts aimed to improve plant yield through introduction of biophysical CCM components [47] [48]. The fact that 2PG/DHAP labeling ratios were not lower in algae compared with plants may result from relaxed specificity of RuBisCO in the former owing to their efficient CCM. Frequently, specificity has been shown to trade-off with higher Vmax for this enzyme [49]. On the background of the labeling

kinetics of 2PG in photoautotrophically grown algae observed here, expression of algal RuBisCO in plants engineered to operate a CCM may assist in realizing their yield increase potential.

Sequence analysis of relevant algal enzymes

The route from the first CO_2 assimilation product, 3PGA, to PEP involves the lower glycolytic enzymes phosphoglycerate mutase (PGM) and enolase (Eno). In higher plants, these enzymes are present in the cytosol, and only in some types of plastids, mostly in seeds or lipid storing tissues. In photosynthetic cells in leaves, provision of the cytosol with PEP requires its export via the triose-phosphate/Pi translocator (TPT) and metabolism via PGM and Eno, and provision of the plastid with PEP requires re-import via the PEP/phosphate translocator (PPT) [50] [51] [52]. *C. reinhardtii*, *C. sorokiniana* and *C. ohadii* genomes do harbor genes predicted to encode plastid PGM but encode only one Eno, which shows the highest similarity to cytosolic plant enolases (Supplementary dataset 1). Assuming this prediction is valid, the joint function of TPT and Eno impose a potential restriction point for C partitioning in algae. The relatively slow labeling of PEP in plants and *C. reinhardtii* compared with *C. sorokinana* and, especially, *C. ohadii* (Table S4 and Figure S3), prompted us to analyze the sequence of the algal proteins compared to their higher plant homologs. This analysis uncovered several unique substitutions in *Chlorella*s, including altered patterns of cysteine residues, and uncertainties related to Eno predicted localization to the cytosol, in addition to deletions in highly conserved domains of TPT in *C. ohadii*.

PGM and Enolase. *C. sorokinana* and *C. ohadii* Eno sequences shared only up to 75% similarity with known sequences. Moreover, at least 2 highly unique cysteines were shared between *C. sorokinana* and *C. ohadii* (C100 and C306 in *C. ohadii*) and another one between several *Chlorella*s (C390), slightly upstream to the conserved C405 (Supplementary dataset 1). The latter change is potentially important due to the redox-sensitivity observed in Eno with cysteine residue in a position corresponding to C377, hypothesized to form a disulphide bond with C405 [53]. It should be noted that cytosolic prediction of *C. reinhardtii* single Eno is supported by the failure to detect the enzyme, its activity, or substrate (2PGA) in this alga in plastid fractions [54]. However, such measurements and especially of metabolites are complicated and no molecular localization evidence has been obtained in either of the algae analyzed in this study. In addition, despite higher similarity to AtENO2 (a second cytosolic Eno), algal Eno sequences harbor a conserved N-terminal extension with no clear function or predicted signal peptide, which is present in the cytosolic AtENOc. Further, a large increase (9.1-fold, P value = 0.001644) in the expression of *C. ohadii* gene encoding (based on similarity) plastid PGM was observed in response to high light treatment (Table S7, extracted from [37]), which in light of the cytosolic prediction of algal Eno seems futile. Possible explanations for these ambiguous observations are: (1) 2PGA is transported via the TPT to *C. ohadii* cytosol with similar affinity to 3PGA, as described for example in maize [55], (2) the presence of an unidentified

plastid enolase in algae, which is possible given this enzyme known moonlighting nature [56], or (3) at least some (e.g. a fraction or under certain conditions) Eno protein is localized to plastids in algae. Furthermore, whereas pPGM and pPDH are upregulated in *C. ohadii* in EIL, plastid Pyruvate kinase isn't (Table S7), and as already outlined, modelling based on these labeling datasets indicated that at least some of the flux to PEP in algae occurs in the plastid (Figure 4 and Table S9-Net fluxes). Thus, it remains to be solved (1) whether a dual targeting for algal Eno takes place, (2) whether gene-expression indeed protein levels in these enzymes, and (3) which are the active transporters in algal cells among the plastid translocators (3PGA, triose-phosphate, PEP).

In addition, recent work had lent support to metabolite channeling between PGM and Eno in mature Arabidopsis leaves [57]. Future work should test whether such mechanism also operates in algal cells, characterize its efficiency compared with Arabidopsis, and examine if and to what extent it plays a role in the rapid flux to PEP in algal cells compared to plants.

TPT. Comparative sequence analysis of TPT revealed less than 82% similarity between *C. sorokinana* and *C. ohadii* TPT to any known homolog, 63.4% to *C. reinhardtii* and only 45.2% to Arabidopsis (or less for other higher plants, Supplementary Dataset 2). All of the substrate-recognizing residues [58] were nevertheless conserved, including H211, K230, Y373, K396 and R397. Interestingly, 12 highly conserved residues (including the highly conserved C330) were absent from *C. ohadii* TPT, a gap shared only with a predicted G6P/Pi translocator-like protein in Arabidopsis. In addition, a highly conserved glycine in position 339 was replaced with arginine or lysine in several *Chlorella*s, and F225 in a conserved pocket was replaced for leucine in several algae, including all three analyzed here. Further experimental studies are needed to learn if differences in catalytic rates or in specificity of algal TPT, or in the properties of Eno, or in expression levels of these proteins may provide an explanation for the rapid labeling of PEP in algae.

PEPC/PPCK. PEPC is found in all plants and green algae [59]. PEPC has been shown to be play pivotal role in C-N metabolism balance in Arabidopsis [60]. PEPC activity is allosterically inhibited by malate, aspartate and glutamate, and activated by G6P and, in algae, glutamine [61]. Importantly, reversible phosphorylation of a serine residue next to its N-terminus (conserved in plant-type PEPCs) by a dedicated PEPC kinase (PPCK) alters its sensitivity to these regulators [62] [63]. In addition, mono-ubiquitination of a conserved lysine residue [64] reduces the K_m for PEP and sensitivity to metabolite regulation. The high fluxes to malate, aspartate and other organic acids in the two *Chlorella*s, compared with *C. reinhardtii* and Arabidopsis (Tables S1, S4 and S5 and Figure 5) prompted us to examine PEPC sequences in algae and higher plants. Strikingly, PEPC variants in both *Chlorella*s lacked the highly conserved allosteric phosphorylated serine and the mono-ubiquitinated lysine. In addition, our analysis uncovered unique cysteine patterns in all three algae compared with higher-plant homologs. This, and the presence of

differences between the different algal sequences, suggests that redox may play an alternative regulatory role in PEPC activity of some of these algae.

Two PEPC variants exist in both *C. reinhardtii* [61] and *C. ohadii* (NCBI accession PRJNA573576), termed PEPC1 and PEPC2. Whereas the genes encoding both enzymes follow similar expression patterns in *C. reinhardtii* [65], they exhibit divergent expression patterns when exposed to different light regimes in *C. ohadii* (Table S7, extracted from [37]). *C. reinhardtii* PEPCs are organized in two forms - a PEPC1 homo-tetramer (Class 1) [65] and a PEPC1/PEPC2 hetero-oligomer (Class 2) [66]. The predominant Class 2, previously regarded as distinctive for algae, exhibited higher catalytic efficiency and affinity to PEP, and different allosteric metabolite regulation [61], but was later also found in developing castor oil seeds [67]. Based on in-vitro activity assays, it was hypothesized that plants form stable Class 2 complexes to maintain high flux from PEP under physiological conditions which inhibit Class-1 PEPC [68]. Comparative sequence analysis of PEPC1 revealed the existence of an N-terminal extension in several *Chlorella*s which was absent from other plants and algae, *C. reinhardtii* included. In addition, pattern of cysteines occurrence was different in several algal strains compared with known PEPC1 homologs, implying redox may play a previously uncharacterized role in regulation of this algal enzyme. Conserved residues were substituted for cysteines in positions 164 and 642 in all three algae, 426 and 491 only in *Chlorella*s, and 770 in *C. reinhardtii*. Conversely, conserved cysteines were substituted in positions 334 and 732 in *Chlorella*s, and 307, 464 and 468 in all three algae (Supplementary dataset 3). Moreover, 4 conserved residues were absent only in *C. ohadii* PEPC1, reducing the distance between C459 to the *Chlorella*-specific C491. In the case of PEPC2, *Chlorella* variants retained the conserved allosteric plant N-terminus which is absent from *C. reinhardtii*, but lost the highly conserved serine in position 16, suggesting altered mode of allostery (Supplementary dataset 4). This possibility is in line with highly dissimilar PPCK in *C. ohadii* and *C. sorokiniana* compared with publicly available hits (< 50% in most cases), whereas as expected no homolog can be found in *C. reinhardtii*. C349 was found solely in *C. sorokiniana* and *C. ohadii*, with a unique 9 residue insert in the latter, setting an additional cysteine in C356 (Supplementary dataset 4). Further, in both variants of PEPC, the highly conserved lysine involved in this enzyme regulation via mono-ubiquitination (K679 and K1025 in PEPC1 and PEPC2 alignments, respectively; Supplementary datasets 3 and 4), was replaced with either alanine (PEPC1 in *C. reinhardtii* and *C. ohadii*), serine (PEPC1 in *C. sorokiniana*), or glutamate (PEPC2 in *C. ohadii* and *C. sorokiniana*).

Recently, it has been shown that a single arginine residue (884 in *Flaveria*) in the C_3-type PEPC regulates the binding of the inhibitory malate, which is replaced by glycine in the C_4-type, resulting in the increased kinetic efficiency of the latter [69]. Arginine in this position provides additional hydrogen bonding to the inhibitor, and thereby promotes stronger binding to the inhibitory site. In all algae examined

here, it was replaced with lysine (K955) in the PEPC1 variant (Supplementary dataset 3) or proline (P1285) in the PEPC2 variant (Supplementary dataset 4). [70].

mPDH/PDK. The mitochondrial pyruvate dehydrogenase (mPDH) complex is the key step for entry of C into the TCA cycle, occupying a crucial position at the intersection of anabolic and catabolic metabolism [71]. Plant mPDH are regulated by product inhibition (NADH and acetyl-CoA), but not by Ca_2^+ and not directly by ATP/ADP ratio, unlike their mammalian counterpart ([72] and references therein). In addition, coordinated activity of dedicated protein kinases (PDK) and phosphatases (PDP) regulate mPDH activity via reversible phosphorylation of its E1α subunit. The former is activated synergistically by ATP and NH_4^+ (as a mitochondrial photorespiratory by-product) in the light to inhibit mPDH in both C_3 and C_4 plants [73] [72]. This explains the very slow labeling of citrate and other TCA intermediates and amino acids downstream of mPDH in the light in higher plants [74] [7] [13]. The rapid labeling of citrate, succinate and fumarate in the three algae that we studied (Figures 5, Extended Data figure 3 and Table S5) in the light suggests mPDH is regulated differently in photosynthetic algal cells.

Analysis of mPDH E1α sequence revealed that despite less than 84% similarity between *C. sorokinana* and *C. ohadii* mPDH to any known homolog (66% to *C. reinhardtii*), both the 2 phosphorylated serine residues (positions 321 and 327) [75] and the essential active-site cysteine residue (106) [76] were conserved in all algae and plant sequences (Supplementary dataset 5). However, a unique cysteine (position 272) was found only in very few *Chlorella*s instead of a highly conserved valine in the putative subunit interaction domain, with an additional unique cysteine 36 residues upstream to it, shared by the same *Chlorella* species. The region of these substitutions also abuts the first phosphorylation sites and may affect the nature of the phosphorylation event rather than subunit interactions [77]. To further explore the potential of redox regulation in this system, we analyzed the sequences of algal PDK, which are found in two forms in all of the algae analyzed here. One of these algal variants demonstrates similarity to non-plant and mostly fungal orthologs (Supplementary dataset 6). In the other, the highly conserved asparagine at position 287, termed "n" [78]) and glutamate at position n-4 (283) of the so-called active site N-box, were also found in all algal homologs (Supplementary dataset 7). Notably, the histidine at n-8 (279), that was conserved in mammals but not in plants (where it was substituted for a conserved leucine), was also conserved in all *Chlorella*s. This residue was predicted to be involved in the catalytic efficiency of the mammalian PDK, as supported by its substitution with alanine [78]. There was no evidence, however, for lower catalytic efficiency of plant PDK, nor for improved catalytic performance when the leucine at n-8 was replaced with histidine. In addition, 2 unique cysteines were found in several *Chlorella*s in positions 239 and 233 (Supplementary dataset 7), the latter instance is conserved also in mammalian PDK, which was reported to be redox-inactivated leading to higher mPDH activity [79]. Interestingly, this mechanism was pronounced

under carbohydrate excess and low ATP demand, which may well be the case in our photoautotrophic growth conditions for these algae, leading to excess C anaplerotic investment into respiration, protein synthesis and growth.

An alternative explanation to a distinctive regulation of algal and higher plant mPDH may be found in the reported stimulatory effects of polyamines on mammalian but not pea PDP [80]. We previously described accelerated respiration and thereafter growth in running *C. ohadii* cultures supplemented with external putrescine.

Supplementary Tables

Tables S1, S2, S4, S5 and S8 are provided in a unified excel file.

Table S3 - Metabolic content in the time kinetic pulse experiments. Amounts are expressed as nmol g^{-1} DW. Metabolites were quantified by GC-MS, LC-MS/MS or (for PEP) by enzymatic assay. Data are mean ± SD (n = 3 biologically independent experiments). The Table is shown on the next page.

Mean absolute levels (nmol g-^{1} DW)	*C. reinhardtii*	*C. sorokiniana*	*C. ohadii*	*C. ohadii* EIL
**DHAP**	15.34±5.82	42.78±15.01	46.61±11.61	52.63±12.06
**FBP**	20.77±8.78	69.81±35.09	42.27±17.60	128.68±25.58
**SBP**	31.56±15.40	36.08±13.11	39.53±17.12	66.85±14.63
**S7P**	19.21±19.06	49.76±44.58	53.39±55.45	63.8±62.07
**R5P**	3.39±2.08	16.89±7.65	13.44±4.00	24.06±6.39
**Pentose-phosphates**	4.16±2.26	43.07±22.62	16.7±6.15	41.39±13.73
**RuBP**	127.57±47.96	1588.59±674.76	268±64.14	1480.57±361.78
**F6P**	18.74±11.15	94.33±33.58	58.67±32.60	92.51±31.86
**G6P**	65.75±29.83	304.17±81.92	145.29±36.36	203.26±57.02
**G1P**	15.51±4.91	75.31±18.88	42.56±16.24	74.18±19.02
**ADP-Glucose**	4.68±1.72	16.72±5.43	8.31±3.60	16.47±4.41
**UDPG**	26.8±8.49	86.46±18.04	134.42±25.04	86.62±16.98
**2PG**	2.91±1.17	7.19±3.19	3.43±1.55	13.59±2.73
**PEP**	1372.59±282.31	2145.89±383.79	4053.84±1463.95	3243.43±499.13
**Malate**	44.46±18.44	1140.69±396.66	223.35±80.96	1085.83±199.63
**Aspartate**	86.85±71.97	1350.58±365.78	465.56±118.15	546.97±127.03
**2OG**	8.22±3.27	30.11±13.01	23.48±9.27	48.31±20.77
**Succinate**	32.99±27.14	31.39±14.68	46.78±13.66	15.28±4.00
**Glutamate**	126.17±95.91	1394.27±294.34	656.81±233.01	906.59±125.95
**Pyruvate**	483.8±129.77	735.88±124.12	608.31±74.65	632.55±102.69
**Citrate**	6888.92±663.52	4801.32±455.06	6884.55±739.15	4791.11±474.05
**Fumarate**	628.86±90.89	870.97±51.93	1047.84±67.34	792.82±45.9
**Glycine**	557.2±58.08	2105.4±222.79	3097.42±556.16	4320.61±158.35
**Proline**	442.99±66.02	1126.81±447.77	5284.72±1048.47	5283.86±1107.38
**Shikimate**	1866±982.14	2334.95±856.47	4641.9±1093.36	3337.79±1290.26
**Galactose**	2840.72±672.64	1076.18±149.2	3009.01±1327.26	3587.52±1081.85
**Glucose**	114.24±26.39	160.71±24.98	384.97±85.97	622.84±186.86
**Mannose**	15.57±7.04	58.25±10.88	80.01±35.2	201.53±53.74
**Sucrose**	50.79±30.6	3409.65±178.65	5106.66±776.7	4441.37±234.44
**Gly3P**	8073.61±2036.13	32275.62±9413.88	49064.08±23659.55	34290.07±7386.91
**Putrescine**	31271.83±3342.35	3891±372.87	5799.79±264.43	4028.01±169.98
**Mean relative levels (peak area g^{-1} DW in 10^{9})**				
**3PGA**	3.82±2.53	29.93±13.90	13.51±5.57	24.82±11.57

Table S6 - Protein synthesis rates (PSR, % h^{-1}) as calculated based on glycine ($PSR_{glycine}$) or aspartate ($PSR_{aspartate}$) for the algae analyzed here, and Arabidopsis (extracted from [13] for pulse time points 0 to 8 h), and growth rates (OD735 h^{-1}) for the different algae. Calculation of PSR is detailed in the methods and is based on [13]. Briefly, the difference between mean label incorporated into protein-bound aspartate or glycine (expressed as % enrichment of each residue in protein) at the start (120 m) and end (300 m) of the analyzed time interval is divided by the mean enrichment (%) of their free form (free), and duration of the time interval to provide the protein synthesis rate (PSR) as increase in enrichment per hour. Note that *C. reinhardtii* has higher protein content than *C. sorokiniana*, *C. ohadii* at LL or EIL (248, 135, 160 and 184 mg g^{-1} DW, respectively). When this is taken into account, the absolute rates of protein synthesis (averaged for the data for aspartate and glycine) are 3.76, 6.94, 6.75, and 12.82 mg g^{-1} DW h^{-1}, respectively. For comparison, the protein synthesis rate (PSR) in Arabidopsis in the light period is provided, calculated from data for enrichment in [13]. In Arabidopsis, alanine had both high enrichment in its free pool and slow decay during dark chase, and was therefore used in preference to other amino acids; the rate estimated based on alanine labeling kinetics was 1.95% h^{-1} [13]. For pairwise inter-algal comparisons, each alga was compared to *C. ohadii* at LL (paired two-tailed t-test; one asterisk, P < 0.05).

	**Aspartate**				**Glycine**				
	120 m (%)	300 m (%)	Free (%)	PSR (% h^{-1})	120 m (%)	300 m (%)	Free (%)	PSR (% h^{-1})	Growth rate (OD 735 h^{-1})
C. reinhardtii	1.28	3.18	56.3	1.11**	1.33	3.69	41.6	1.92*	0.0033
C. sorokiniana	4.78	11.93	51.1	4.67	4.48	13.4	53.4	5.61	0.0085
C. ohadii	6.45	13.92	66.2	3.77	6.43	14.42	57.4	4.67	0.0092
***C. ohadii* EIL**	9.77	23.43	69.7	6.6*	12.1	24.3	66.1	7.33*	0.0126
	0 h (%)	8 h (%)	Free (%)	PSR (% h^{-1})	0 h (%)	8 h (%)	Free (%)	PSR (% h^{-1})	
**Arabidopsis**	0.82	8.58	81.52	1.19	1.56	14.06	76.12	2.05	-

Table S7 - Differential gene expression genes discussed in the text following shift from LL to EIL in *C. ohadii* cultures (extracted from [37]).

**Gene**	**Log_2 fold-change**	**FDR**
pPGM	1.89	0.00874
PEPC1	3.18	0.001644
PEPC2	0.861	0.218055
pPDH subunit alpha	1.92	0.001644
pPDH subunit beta	1.44	0.019
mPDH subunit alpha	0.58	0.34
mPDH subunit beta	0.54	0.36

Supplementary Figures

Figure S1 - GAP analysis (see methods) of metabolite enrichment levels at 0-40 s (a) and 0-300 min (b) time scales, estimating $k = 4$ as the number of clusters. Values represent GAP score for a given number of clusters ± standard error of the bootstrapped gap values (n=50 bootstrap samples).

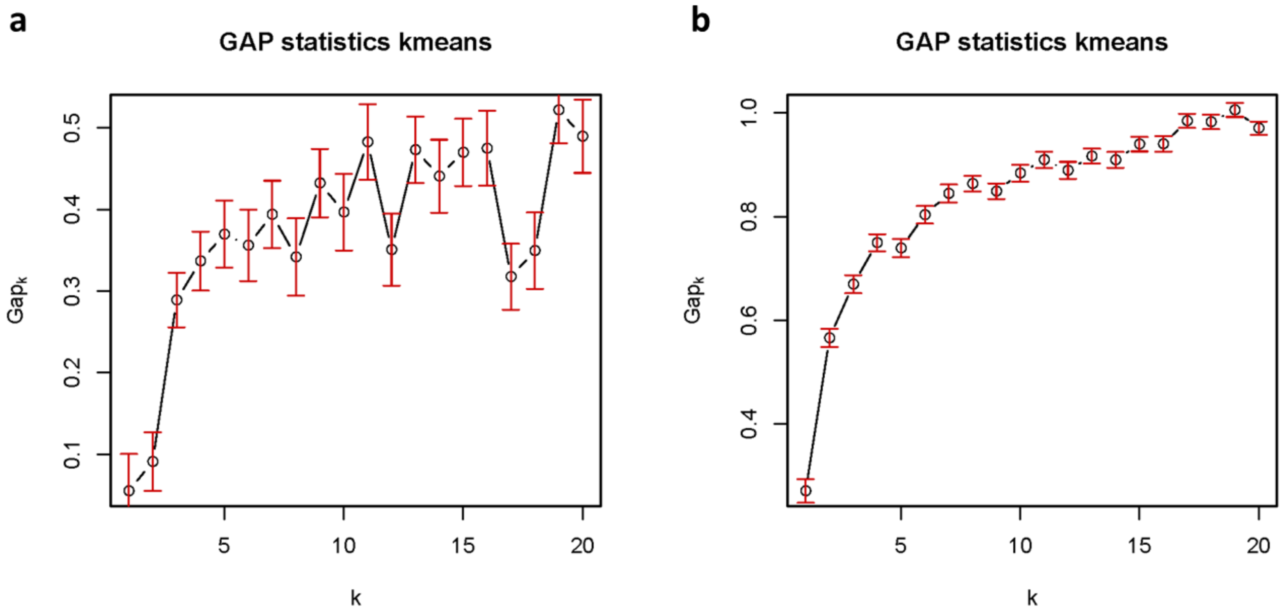

<u>Figure S2</u> - Oxygen evolution rates were measured in a closed stirred cuvette with a Clark-type electrode. Cells were transferred from the bioreactor into the electrode, and O_2 levels were measured under the same light levels as provided in the bioreactors (LL or EIL) for 2 min, and the rate of O_2 evolution was estimated from the slope over time. Data are mean ± SD (n=3 biologically independent experiments).

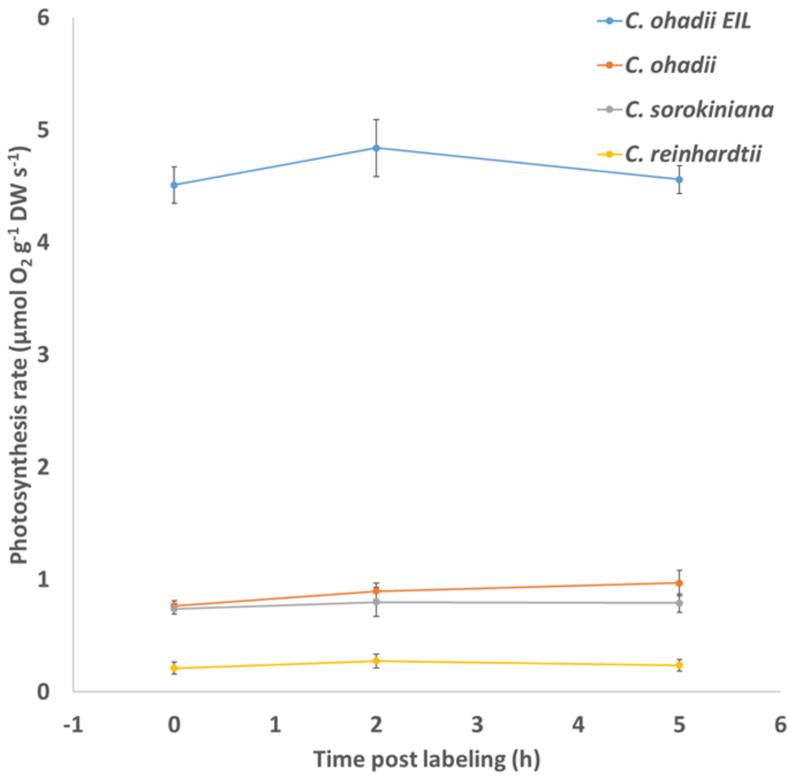

Figure S3 - Regression plots for labeling kinetics of intermediates vs. DHAP (quantified by LC-MS/MS). Data are mean ± SD of enrichment of each presented metabolite and DHAP at each data point (n=3 biologically independent experiments). Slopes extracted from similar analysis for all metabolites analyzed at 0-40 s in algae, Arabidopsis and maize are detailed in Table S4. For Arabidopsis and maize, enrichment at 40 s was interpolated linearly from 20 and 45 s, or 10 and 60 s, respectively, hence SD are not presented.

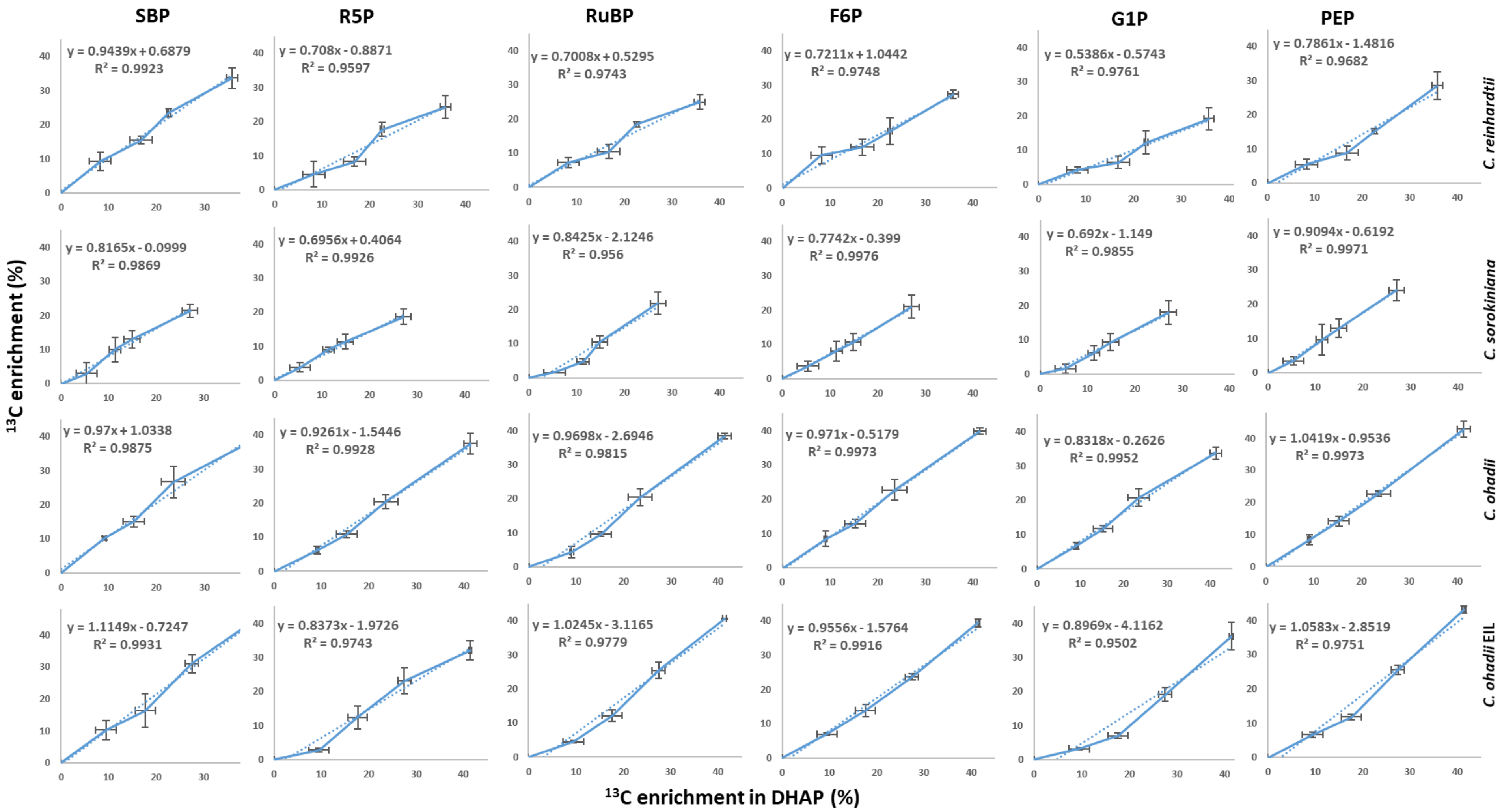

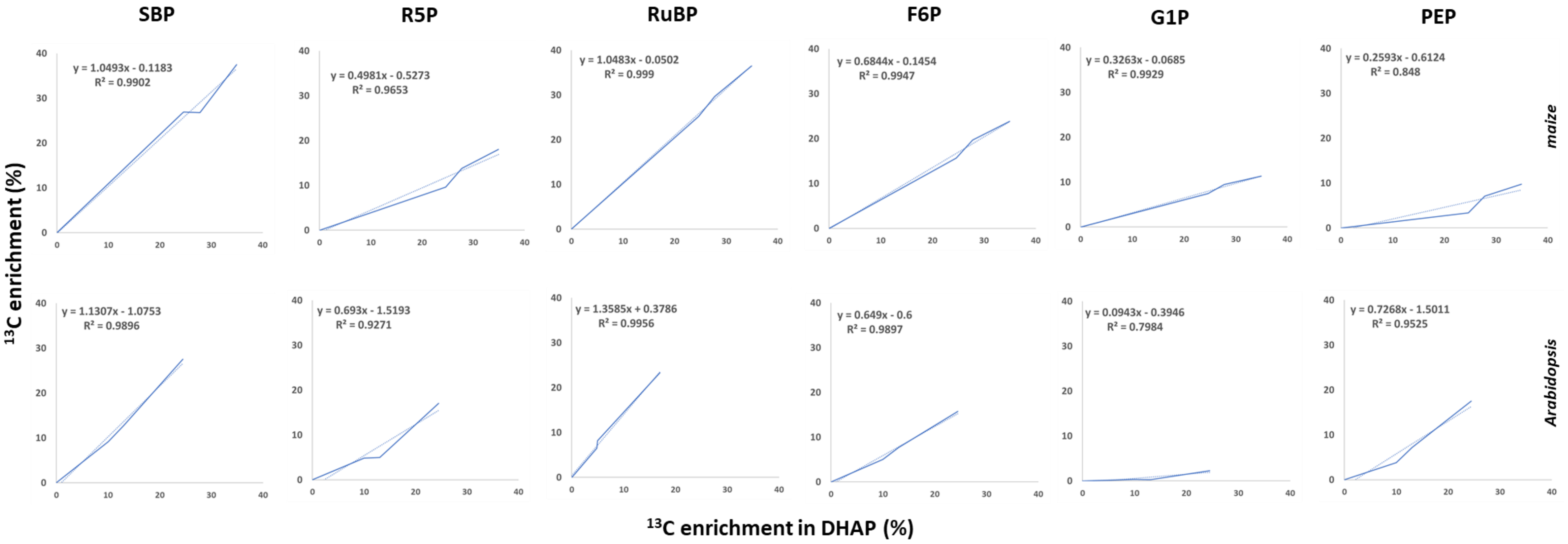
SBP
R5P
RuBP
F6P
G1P
PEP
maize
Arabidopsis
13C enrichment (%)
13C enrichment in DHAP (%)
y = 1.0493x - 0.1183
R² = 0.9902
y = 0.4981x - 0.5273
R² = 0.9653
y = 1.0483x - 0.0502
R² = 0.999
y = 0.6844x - 0.1454
R² = 0.9947
y = 0.3263x - 0.0685
R² = 0.9929
y = 0.2593x - 0.6124
R² = 0.848
y = 1.1307x - 1.0753
R² = 0.9896
y = 0.693x - 1.5193
R² = 0.9271
y = 1.3585x + 0.3786
R² = 0.9956
y = 0.649x - 0.6
R² = 0.9897
y = 0.0943x - 0.3946
R² = 0.7984
y = 0.7268x - 1.5011
R² = 0.9525

Figure S4 - 2PG levels per unit DW. Data for algae and Arabidopsis are mean ± SD (n=3 biologically independent experiments). Data for maize were extracted from [81]. As already described, levels per DW for plants are assuming 91% water content. Plant-algal comparisons (a) was performed using one-way ANOVA (P = 1.92xE^{-49}). For pairwise inter-algal comparisons (b), each alga was compared to *C. ohadii* at LL (paired two-tailed t-test; * - P = 5.2xE^{-4}, ** - P = 1.6xE^{-11}).

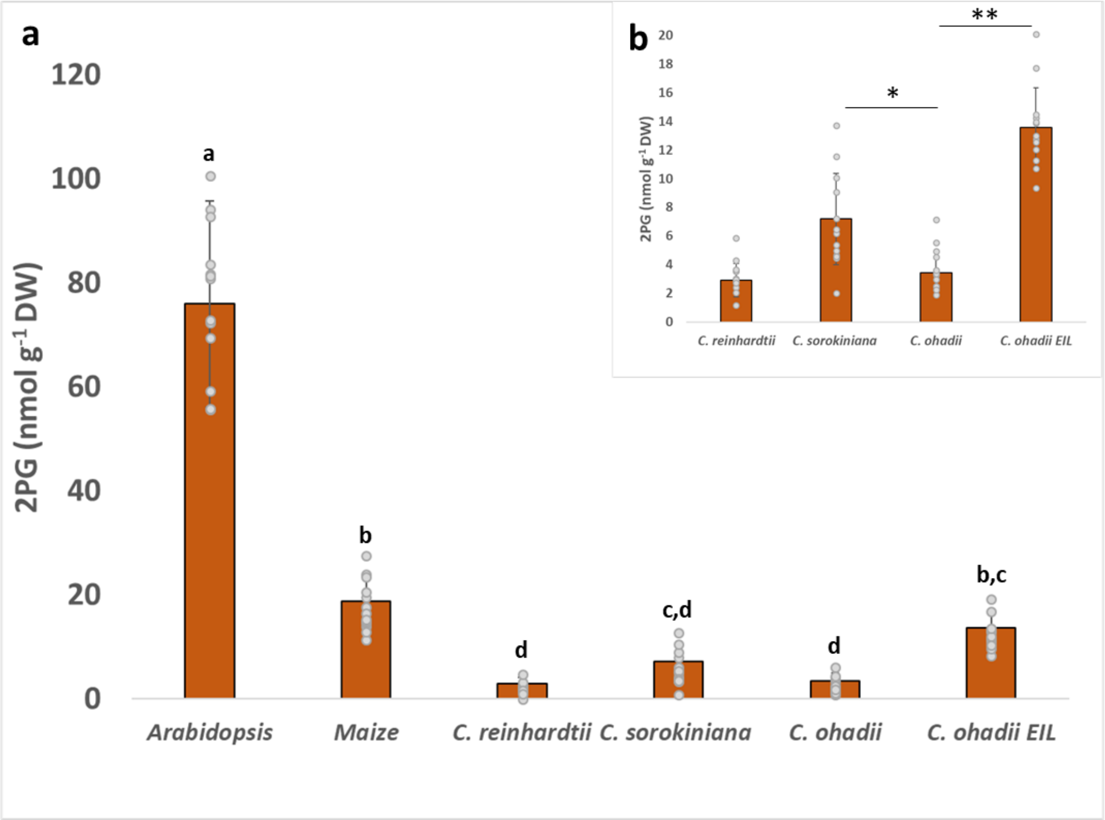

Figure S5 - Time-course of mass distribution of metabolites analyzed by GC-MS at 0-300 min. The relative abundance of each isotopomer (m+n) for a given metabolite is represented; n is the number of ^{13}C atoms incorporated.

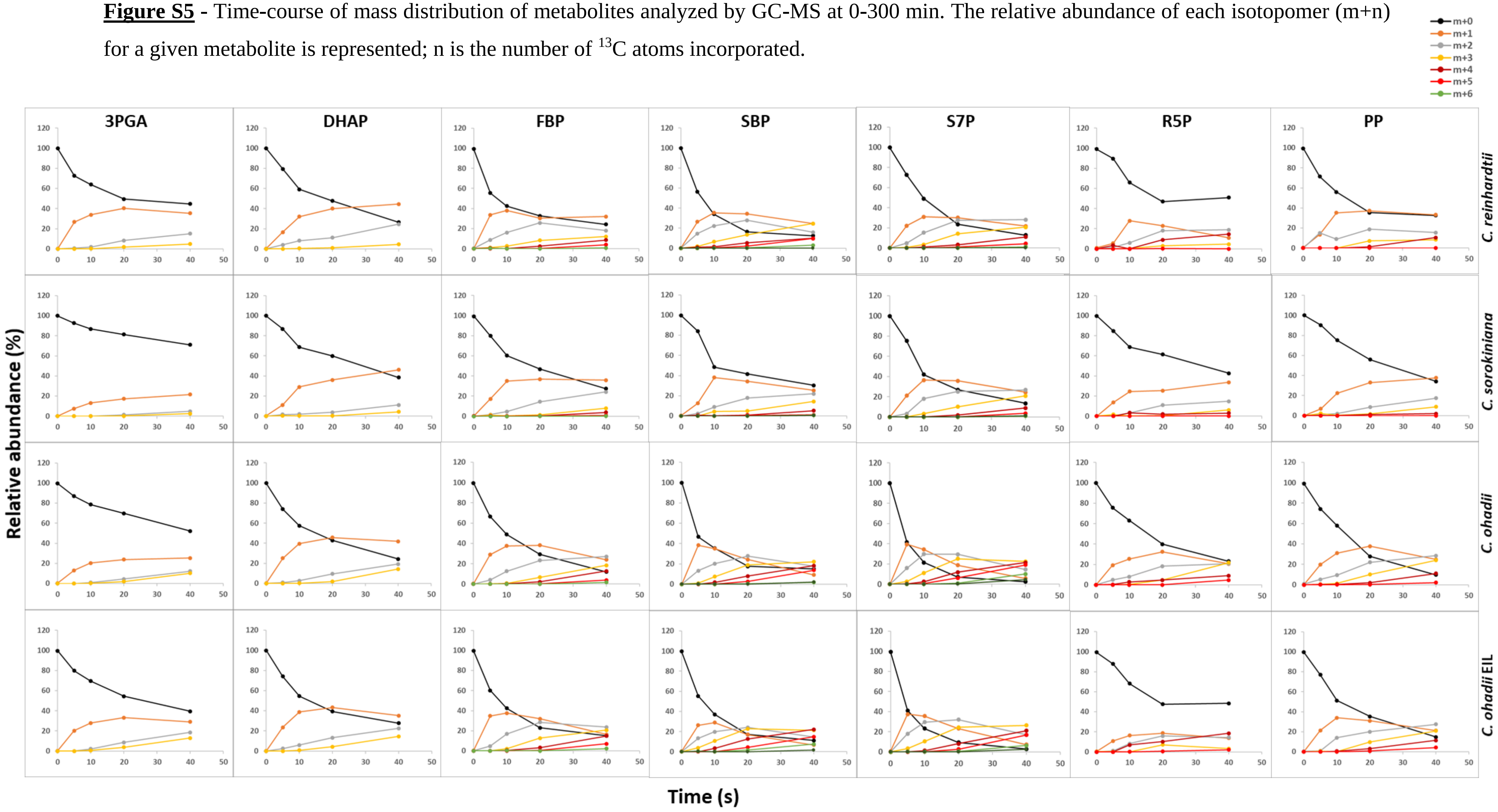

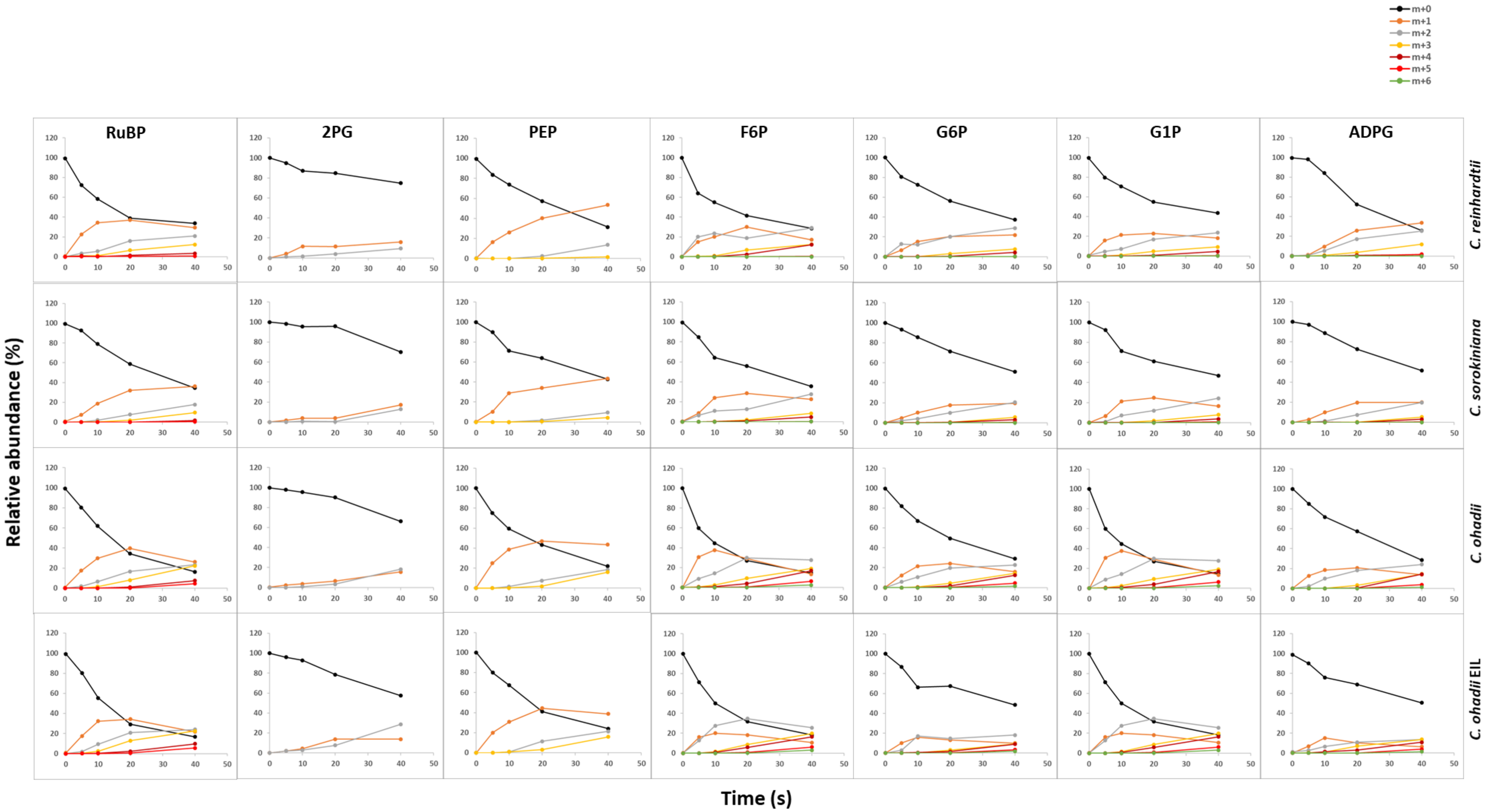
m+0
m+1
m+2
m+3
m+4
m+5
m+6
RuBP
2PG
PEP
F6P
G6P
G1P
ADPG
C. reinhardtii
C. sorokiniana
C. ohadii
C. ohadii EIL
Relative abundance (%)
Time (s)

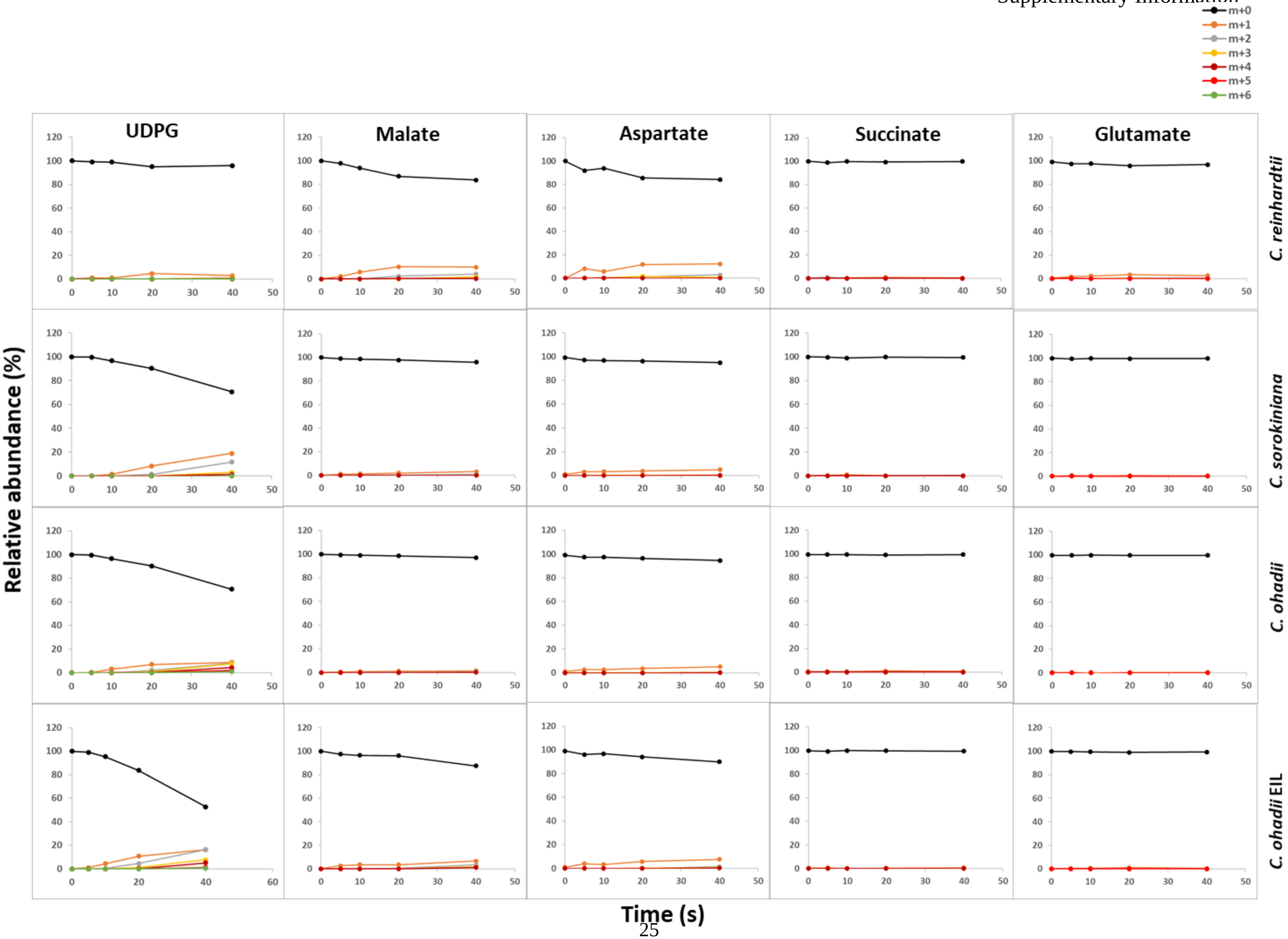
m+0
m+1
m+2
m+3
m+4
m+5
m+6
UDPG
Malate
Aspartate
Succinate
Glutamate
C. reinhardtii
C. sorokiniana
C. ohadii
C. ohadii EIL
Relative abundance (%)
Time (s)

Figure S6 - Time-course of mass distribution of metabolites analyzed by GC-MS at 0-300 min. The relative abundance of each isotopomer (m+n) for a given metabolite is represented; n is the number of ^{13}C atoms incorporated.

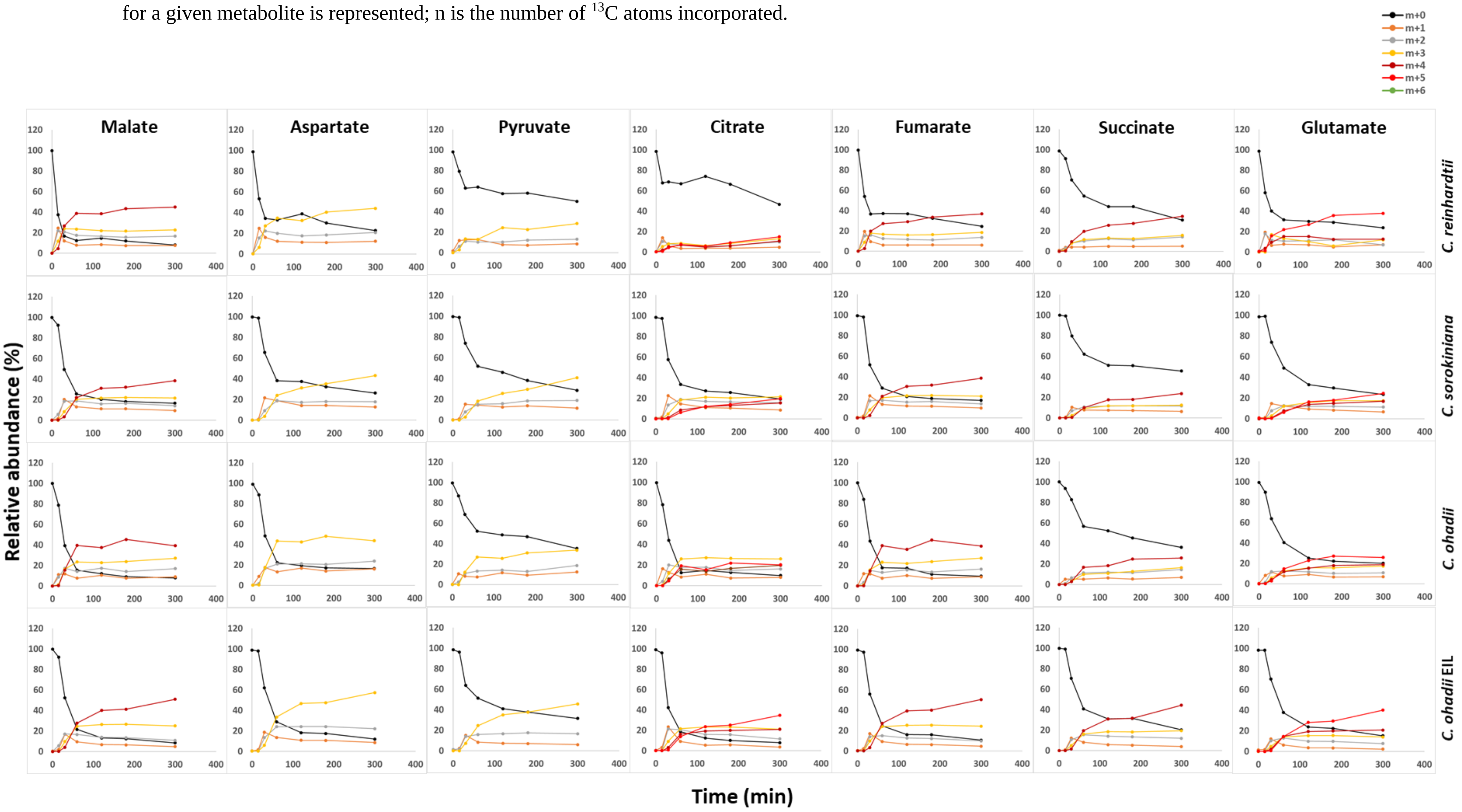

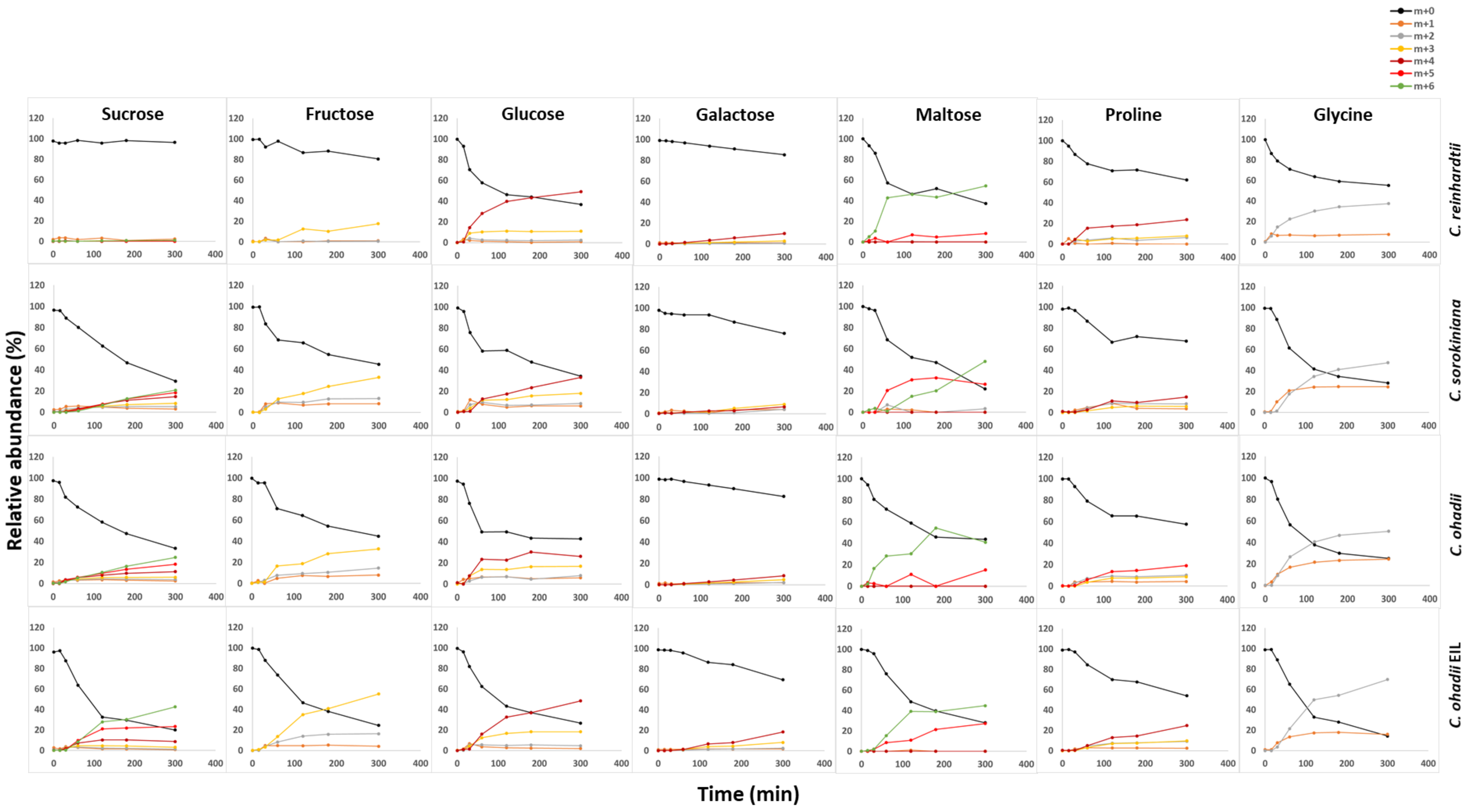
m+0
m+1
m+2
m+3
m+4
m+5
m+6
Sucrose
Fructose
Glucose
Galactose
Maltose
Proline
Glycine
C. reinhardtii
C. sorokiniana
C. ohadii
C. ohadii EIL
Relative abundance (%)
Time (min)

Figure S7 - Growth curves of algal cultures. Experiments were performed with batch cultures on HP medium (methods) in photobioreactors, where growth was monitored based on OD735nm measurements performed every 5 min. Growth rates (OD735 hr^{-1}) were calculated from the slope during the labeling pulse window (0-300 min, starting after 30, 55, 58 and 87 hours culture for *C. ohadii* in EIL and *C. ohadii*, *C. sorokiniana* and *C. reinhardtii* in LL, respectively, these times wwre chosen to have approximately the same OD for the *Chlorella*s, but for the very slow growing *C. reinhardtii* a lower OD was necessary), denoted with red arrows for each alga, and correspond to 0.019, 0.032, 0.037 and 0.052 g DW g^{-1} DW hr^{-1} for *C. reinhardtii*, *C. sorokiniana*, and *C. ohadii* in LL and EIL, respectively. Similar growth curves were obtained from 3 biologically independent replicates.

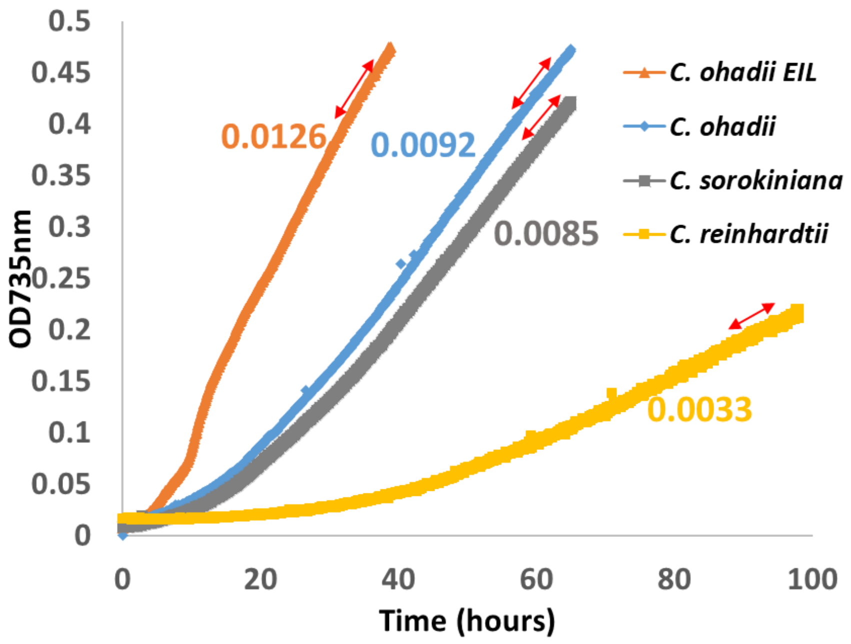

Figure S8 - GC-MS analysis of Gly3P in plant and algal samples.

Identification of trimethylsilylated Gly3P by GC-MS profiling analysis. Mass spectral match and retention index suffice to identify trimethylsilylated Gly3P by at least two types of orthogonal chemical information in agreement with previous recommendations, e.g. [82] [83] [84]. Note that chemical derivatization for GC-MS analysis generates a single fully trimethylsilyated analyte (chemical derivative) of Gly3P. Potential annotation ambiguity arising from the isomer glycerol-2-phosphate is solved by differing mass spectra, e.g. the inverted relative abundances of fragments m/z 357 and m/z 243, where m/z 357 is abundant but m/z 243 low in mass spectra of trimethylsilylated Gly3P (inserted mass spectrum of an algae sample), and a chromatographic retention shift of ~36 retention index units between isomers [85]. Reference information and source of authenticated chemicals are available from http://gmd.mpimp-golm.mpg.de/. The retention index variation between co-chromatographed, authenticated Gly3P (black) and biological samples (colored) is within technical limits of gas chromatography column manufacturers, column age, and metabolomic laboratory [86]. The mass fragment $[m/z = 357]^+$ chosen for selected ion chromatogram-displays results from cleavage of the ionized molecular ion at indicated position (insert). Arabidopsis rosettes were grown at optimized, non-stressed conditions, and abundance for leaf tissue rosette sample was magnified by a factor of 100 to improve visibility. Note that algal chromatograms represent unnormalized raw chromatography data.

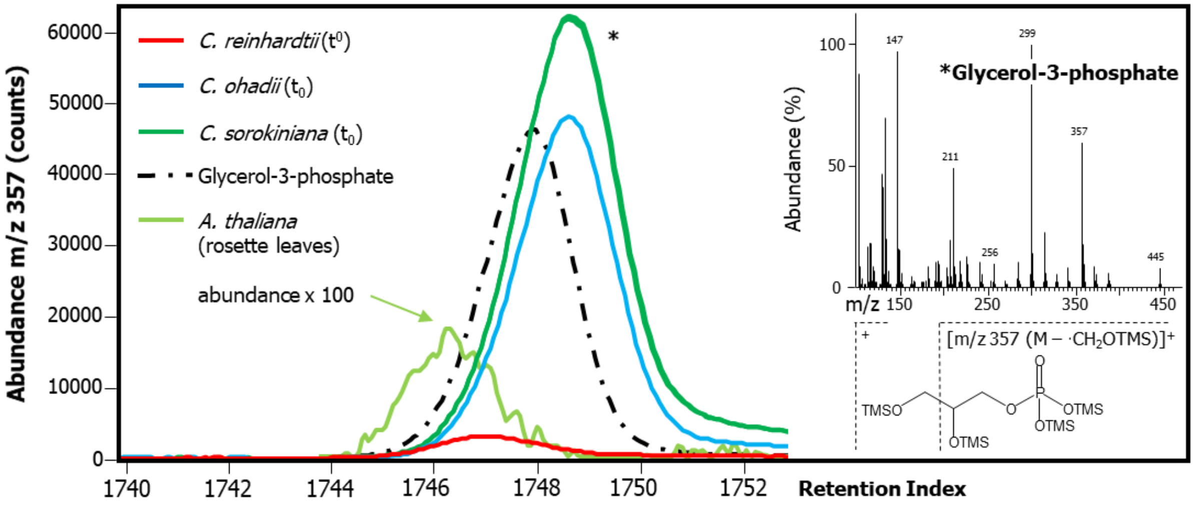

Figure S9 - GC-MS analysis of galactose in plant and algal samples.

(a) Identification of methoxyaminated and trimethylsilylated hexoaldoses by GC-MS profiling analysis. At least two types of orthogonal chemical information are required for metabolomic compound identification in agreement with previous recommendations, e.g. [82] [83] [84]. Chemical derivatization for GC-MS analysis generates an E/Z pair of silylated methoxyamines from each hexoaldose (hexose´ and hexose´´). The E/Z pair and the sugar epimers are chromatographically separated by GC-MS profiling technology. To identify a specific hexose epimer by GC-MS, mass spectral match and chromatographic retention information are required [85]. Reference information and source of authenticated chemicals are available from http://gmd.mpimp-golm.mpg.de/. Mass spectral match identifies the compound class, hexoaldose, but not the specific sugar epimer. As orthogonal information, the specific retention indices of the eight hexoaldose-epimers allow unambiguous annotation. Note that galactose´ and glucose´ are separated by the profiling variant applied in this study using a 5% phenyl–95% dimethylpolysiloxane fused silica type capillary column [87] but co-migrate with rare talose´ and altrose´, respectively. Retention indices of the hexoses´´ serve as third orthogonal criterion for unambiguous annotation. However, even retention indices may slightly differ with gas chromatography column manufacturer, column age, and metabolomic laboratory [86]. For this reason we align gas chromatographic retention of hexoses´(´´) in each analysis-batch by co-chromatography of an authenticated glucose standard in a chemically and a biologically defined reference mixture [87]. Selected ion chromatograms of m/z = 319 u were maximum normalized. The fragment $[m/z = 319]^+$ results from cleavage of the ionized molecular ion followed by neutral elimination of HOTMS as indicated by the insert.

(b) Annotation of methoxyaminated and trimethylsilylated hexoaldoses in GC-MS profiles of *Chlamydomonas reinhardtii*, *Chlorella ohadii* and *Chlorella sorokiniana*. Overlay of co-chromatographed algal metabolite preparations and a glucose reference sample (line formatting as given in (a)). Representative samples were taken at t_0, i.e. before ^{13}C-labelling, to represent the natural, ambient abundance of the selected mass fragment m/z = 319 and exemplary mass spectrum of algal galactose´ (* and insert). The y-axis represents arbitrary abundance, i.e. mass spectral counts. Note that algal chromatograms represent unnormalized raw chromatography data.

(c) Annotation of methoxyaminated and trimethylsilylated hexoaldoses in GC-MS profiles of Arabidopsis leaf tissue. Overlay of co-chromatographed metabolite preparations from leaf tissue of Arabidopsis rosettes at non-stressed, optimized growth conditions and mannose, galactose and glucose reference samples (line formatting as given in (a)). We show a y-axis magnification of the Arabidopsis chromatogram to demonstrate the presence of minor amounts of mannose and galactose next to abundant glucose in this tissue.

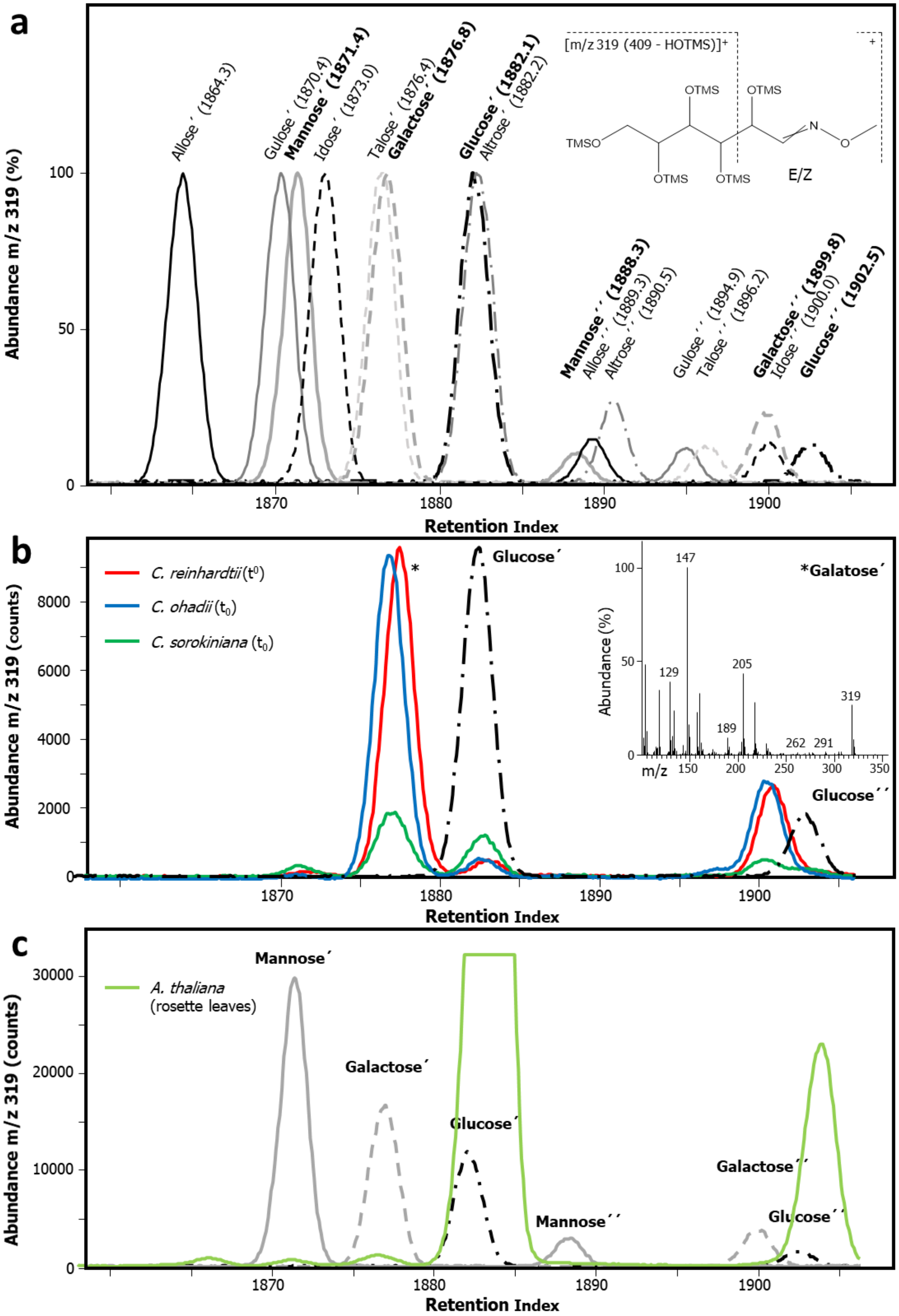
a
Allose´ (1864.3)
Gulose´ (1870.4)
Mannose´ (1871.4)
Idose´ (1873.0)
Talose´ (1876.4)
Galactose´ (1876.8)
Glucose´ (1882.1)
Altrose´ (1882.2)
[m/z 319 (409 - HOTMS)]+
OTMS
TMSO
N
O
E/Z
Mannose´´ (1888.3)
Allose´´ (1889.3)
Altrose´´ (1890.5)
Gulose´´ (1894.9)
Talose´´ (1896.2)
Galactose´´ (1899.8)
Idose´´ (1900.0)
Glucose´´ (1902.5)
Abundance m/z 319 (%)
Retention Index
b
C. reinhardtii (t0)
C. ohadii (t0)
C. sorokiniana (t0)
Glucose´
*Galatose´
Abundance (%)
m/z
147
129
205
189
262
291
319
Glucose´´
Abundance m/z 319 (counts)
Retention Index
c
A. thaliana
(rosette leaves)
Mannose´
Galactose´
Glucose´
Mannose´´
Galactose´´
Glucose´´
Abundance m/z 319 (counts)
Retention Index

Figure S10 - Kinetics of $^{13}CO_2$ supply via bubbling (a) through algal cultures or (b) HP medium or DDW - $^{12}CO_2$ level monitoring at the pulse, as measured on the exhaust of algal cultures/medium/DDW using LI-850 gas analyzer (LI-COR Nebraska, US, see methods). For (a). Cultures of same conditions presented in Figure 3 were grown to a density corresponding to T0 for each alga and condition. Thereafter, bubbling with natural air was rapidly changed (red arrow) to ^{13}C-based synthetic labeled air mixture (see methods). Reciprocally, ~20 min after the first change, bubbling was rapidly replaced again for natural air (green arrow).

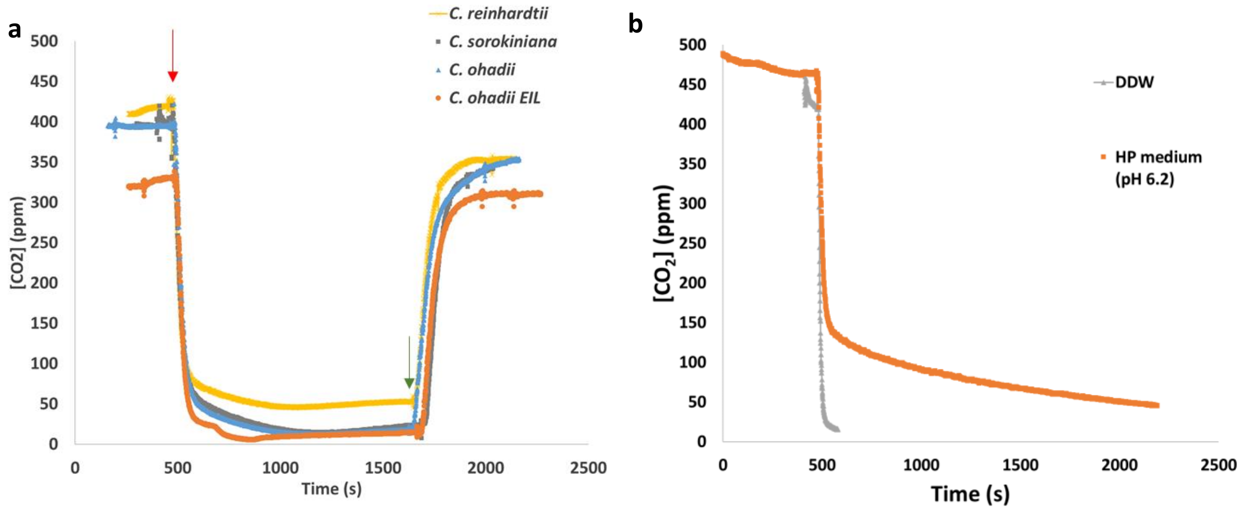

Figure S11 - Maltose relative levels per unit DW. Data are mean ± SD (n=3 biologically independent experiments). For pairwise inter-algal comparisons (b), each alga was compared to *C. ohadii* at LL (paired two-tailed t-test; *, P = 1.7xE^{-4}).

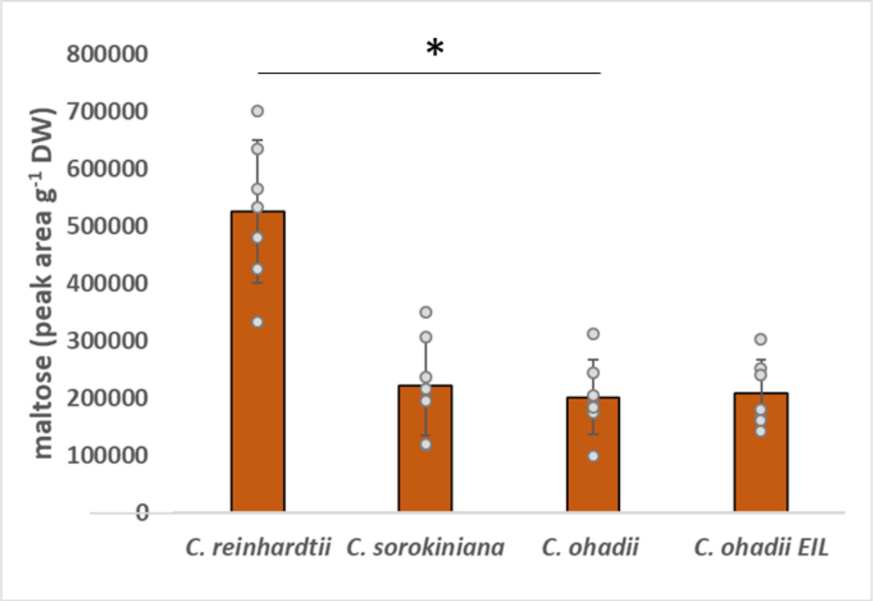

References

1. Stitt, M., J. Lunn, and B. Usadel, *Arabidopsis and primary photosynthetic metabolism - more than the icing on the cake.* Plant J, 2010. **61**(6): p. 1067-91.
2. Arrivault, S., et al., *Use of reverse-phase liquid chromatography, linked to tandem mass spectrometry, to profile the Calvin cycle and other metabolic intermediates in Arabidopsis rosettes at different carbon dioxide concentrations.* The Plant Journal, 2009. **59**(5): p. 826-839.
3. Mettler, T., et al., *Systems analysis of the response of photosynthesis, metabolism, and growth to an increase in irradiance in the photosynthetic model organism Chlamydomonas reinhardtii.* Plant Cell, 2014. **26**: p. 2310-2350.
4. Sulpice, R., et al., *Arabidopsis Coordinates the Diurnal Regulation of Carbon Allocation and Growth across a Wide Range of Photoperiods.* Molecular Plant, 2014. **7**(1): p. 137-155.
5. Gerhardt, R. and H.W. Heldt, *Measurement of Subcellular Metabolite Levels in Leaves by Fractionation of Freeze-Stopped Material in Nonaqueous Media.* Plant Physiol, 1984. **75**(3): p. 542-547.
6. Prinsley, R.T., K.-J. Dietz, and R.C. Leegood, *Regulation of photosynthetic carbon assimilation in spinach leaves after a decrease in irradiance.* Biochimica et Biophysica Acta (BBA) - Bioenergetics, 1986. **849**(2): p. 254-263.
7. Szecowka, M., et al., *Metabolic fluxes in an illuminated Arabidopsis rosette.* Plant Cell, 2013. **25**(2): p. 694-714.
8. Fernandez, O., et al., *Leaf Starch Turnover Occurs in Long Days and in Falling Light at the End of the Day.* Plant Physiol, 2017. **174**(4): p. 2199-2212.
9. Stitt, M. and S.C. Zeeman, *Starch turnover: pathways, regulation and role in growth.* Curr Opin Plant Biol, 2012. **15**(3): p. 282-92.
10. Smith, A.M. and S.C. Zeeman, *Starch: A Flexible, Adaptable Carbon Store Coupled to Plant Growth.* Ann Rev Plant Biol., 2020. **71**(1): p. 217-245.
11. Weise, S.E., et al., *Transcriptional Regulation of the Glucose-6-Phosphate/Phosphate Translocator 2 Is Related to Carbon Exchange Across the Chloroplast Envelope.* 2019. **10**(827).
12. Xu, Y., et al., *The metabolic origins of non-photorespiratory CO2 release during photosynthesis: A metabolic flux analysis.* Plant Physiol, 2021.
13. Ishihara, H., et al., *Quantifying protein synthesis and degradation in Arabidopsis by dynamic 13CO2 labeling and analysis of enrichment in individual amino acids in their free pools and in protein.* Plant Physiol, 2015. **168**(1): p. 74-93.
14. Young, J.D., *INCA: a computational platform for isotopically non-stationary metabolic flux analysis.* Bioinformatics (Oxford, England), 2014. **30**(9): p. 1333-1335.
15. Ma, F., et al., *Isotopically nonstationary 13C flux analysis of changes in Arabidopsis thaliana leaf metabolism due to high light acclimation.* PNAS, 2014. **111**(47): p. 16967-16972.
16. Antoniewicz, M.R., J.K. Kelleher, and G. Stephanopoulos, *Determination of confidence intervals of metabolic fluxes estimated from stable isotope measurements.* Metab Eng, 2006. **8**(4): p. 324-37.
17. Xu, Y., et al., *The metabolic origins of non-photorespiratory CO2 release during photosynthesis: A metabolic flux analysis.* Plant physiology, 2021. **186**(1): p. 297-314.
18. Voigt, J., P. Münzner, and H.-P. Vogeler, *The cell-wall glycoproteins of Chlamydomonas reinhardtii: analysis of the in vitro translation products.* Plant Science, 1991. **75**(1): p. 129-142.
19. Bollig, K., et al., *Structural analysis of linear hydroxyproline-bound O-glycans of Chlamydomonas reinhardtii—conservation of the inner core in Chlamydomonas and land plants.* Carbohydrate Research, 2007. **342**(17): p. 2557-2566.
20. Vidal-Meireles, A., et al., *Regulation of ascorbate biosynthesis in green algae has evolved to enable rapid stress-induced response via the VTC2 gene encoding GDP-l-galactose phosphorylase.* New Phytol, 2017. **214**(2): p. 668-681.

21. Wheeler, G., et al., *Evolution of alternative biosynthetic pathways for vitamin C following plastid acquisition in photosynthetic eukaryotes.* eLife, 2015. **4**: p. e06369.
22. Jüppner, J., et al., *Dynamics of lipids and metabolites during the cell cycle of Chlamydomonas reinhardtii.* The Plant Journal, 2017. **92**(2): p. 331-343.
23. Templeton, D.W., et al., *Separation and quantification of microalgal carbohydrates.* Journal of Chromatography A, 2012. **1270**: p. 225-234.
24. Driver, T., et al., *Two Glycerol-3-Phosphate Dehydrogenases from Chlamydomonas Have Distinct Roles in Lipid Metabolism.* 2017. **174**(4): p. 2083-2097.
25. Zhang, M., et al., *DGAT1 and PDAT1 Acyltransferases Have Overlapping Functions in Arabidopsis Triacylglycerol Biosynthesis and Are Essential for Normal Pollen and Seed Development.* 2009. **21**(12): p. 3885-3901.
26. Fichtner, F., et al., *Functional Features of TREHALOSE-6-PHOSPHATE SYNTHASE1, an Essential Enzyme in Arabidopsis.* 2020. **32**(6): p. 1949-1972.
27. Badger, M.R., A. Kaplan, and J.A. Berry, *The internal inorganic carbon pool of Chlamydomonas reinhardtii: Evidence for a CO_2 concentrating mechanism.* Plant Physiol, 1980. **66**: p. 407-413.
28. Furbank, R.T. and M.D. Hatch, *Mechanism of c(4) photosynthesis: the size and composition of the inorganic carbon pool in bundle sheath cells.* Plant Physiol, 1987. **85**(4): p. 958-64.
29. Arrivault, S., et al., *Metabolite pools and carbon flow during C4 photosynthesis in maize: 13CO2 labeling kinetics and cell type fractionation.* J Exp Bot, 2017. **68**(2): p. 283-298.
30. SMITH, A.M. and M. STITT, *Coordination of carbon supply and plant growth.* 2007. **30**(9): p. 1126-1149.
31. Hatch, M.-D. and T. Kagawa, *Enzymes and functional capacities of mesophyll chloroplasts from plants with C4-pathway photosynthesis.* Archives of Biochemistry and Biophysics., 1973. **159**(2): p. 842-853.
32. Hatch, M.D., *C4 photosynthesis: a unique elend of modified biochemistry, anatomy and ultrastructure.* Biochimica et Biophysica Acta (BBA) - Reviews on Bioenergetics, 1987. **895**(2): p. 81-106.
33. Riebesell, U., *Photosynthesis. Carbon fix for a diatom.* Nature, 2000. **407**(6807): p. 959-60.
34. Johnston, A.M., et al., *Carbon fixation. Photosynthesis in a marine diatom.* Nature, 2001. **412**(6842): p. 40-1.
35. Haimovich-Dayan, M., et al., *The role of C4 metabolism in the marine diatom Phaeodactylum tricornutum.* New Phytol., 2013. **197**(1): p. 177-185.
36. Treves, H., et al., *The mechanisms whereby the green alga Chlorella ohadii, isolated from desert soil crust, exhibits unparalleled photodamage resistance.* New Phytologist, 2016. **210**(4): p. 1229-1243.
37. Treves, H., et al., *Multi-omics reveals mechanisms of total resistance to extreme illumination of a desert alga.* Nature Plants, 2020. **6**: p. 1031–1043.
38. Nam, J.-W., et al., *A General Method for Quantification and Discovery of Acyl Groups Attached to Acyl Carrier Proteins in Fatty Acid Metabolism Using LC-MS/MS.* 2020. **32**(4): p. 820-832.
39. Ort, D.R., et al., *Redesigning photosynthesis to sustainably meet global food and bioenergy demand.* Proceedings of the National Academy of Sciences, 2015. **112**(28): p. 8529-8536.
40. Stitt, M., G. Luca Borghi, and S. Arrivault, *Targeted metabolite profiling as a top-down approach to uncover interspecies diversity and identify key conserved operational features in the Calvin–Benson cycle.* Journal of Experimental Botany, 2021.
41. Simkin, A.J., P.E. Lopez-Calcagno, and C.A. Raines, *Feeding the world: improving photosynthetic efficiency for sustainable crop production.* J Exp Bot, 2019. **70**(4): p. 1119-1140.
42. Vanhercke, T., et al., *Metabolic engineering for enhanced oil in biomass.* Progress in Lipid Research, 2019. **74**: p. 103-129.
43. Leegood, R.C., *Roles of the bundle sheath cells in leaves of C3 plants.* Journal of Experimental Botany, 2008. **59**(7): p. 1663-1673.

44. Gao, C.J., et al., *Implication of reactive oxygen species and mitochondrial dysfunction in the early stages of plant programmed cell death induced by ultraviolet-C overexposure.* Planta, 2008. **227**: p. 755-767.
45. Kaplan, A. and L. Reinhold, *The CO_2 concentrating mechanisms in photosynthetic microorganisms.* Annu. Rev. Plant Physiol. Plant Mol. Biol., 1999. **50**: p. 539-570.
46. Flamholz, A.I., et al., *Revisiting Trade-offs between Rubisco Kinetic Parameters.* Biochemistry, 2019. **58**(31): p. 3365-3376.
47. Atkinson, N., et al., *Introducing an algal carbon-concentrating mechanism into higher plants: location and incorporation of key components.* 2016. **14**(5): p. 1302-1315.
48. Long, B.M., et al., *Carboxysome encapsulation of the CO2-fixing enzyme Rubisco in tobacco chloroplasts.* Nature Communications, 2018. **9**(1): p. 3570.
49. Tcherkez, G.G.B., G.D. Farquhar, and T.J. Andrews, *Despite slow catalysis and confused substrate specificity, all ribulose bisphosphate carboxylases may be nearly perfectly optimized.* 2006. **103**(19): p. 7246-7251.
50. Prabhakar, V., et al., *Phosphoenolpyruvate provision to plastids is essential for gametophyte and sporophyte development in Arabidopsis thaliana.* Plant Cell, 2010. **22**(8): p. 2594-617.
51. Joyard, J., et al., *Chloroplast proteomics highlights the subcellular compartmentation of lipid metabolism.* Progress in Lipid Research, 2010. **49**(2): p. 128-158.
52. Voll, L.M., et al., *Antisense inhibition of enolase strongly limits the metabolism of aromatic amino acids, but has only minor effects on respiration in leaves of transgenic tobacco plants.* New Phytol, 2009. **184**(3): p. 607-18.
53. Anderson, L.E., L. Alex Dong, and F.J. Stevens, *The enolases of ice plant and Arabidopsis contain a potential disulphide and are redox sensitive.* Phytochemistry, 1998. **47**(5): p. 707-713.
54. Polle, J.E.W., et al., *Carbon partitioning in green algae (chlorophyta) and the enolase enzyme.* Metabolites, 2014. **4**(3): p. 612-628.
55. Gross, A., et al., *Comparison of the kinetic properties, inhibition and labelling of the phosphate translocators from maize and spinach mesophyll chloroplasts.* Planta, 1990. **180**(2): p. 262-271.
56. Paludo, G.P., et al., *Systems biology approach reveals possible evolutionarily conserved moonlighting functions for enolase.* Computational Biology and Chemistry, 2015. **58**: p. 1-8.
57. Zhang, Y., et al., *A moonlighting role for enzymes of glycolysis in the co-localization of mitochondria and chloroplasts.* Nature Communications, 2020. **11**(1): p. 4509.
58. Lee, Y., et al., *Structure of the triose-phosphate/phosphate translocator reveals the basis of substrate specificity.* Nat Plants, 2017. **3**(10): p. 825-832.
59. O'Leary, B., J. Park, and W.C. Plaxton, *The remarkable diversity of plant PEPC (phosphoenolpyruvate carboxylase): recent insights into the physiological functions and post-translational controls of non-photosynthetic PEPCs.* Biochem J, 2011. **436**(1): p. 15-34.
60. Shi, J., et al., *Phosphoenolpyruvate Carboxylase in Arabidopsis Leaves Plays a Crucial Role in Carbon and Nitrogen Metabolism.* Plant Physiology, 2015. **167**(3): p. 671.
61. Rivoal, J., W.C. Plaxton, and D.H. Turpin, *Purification and characterization of high- and low-molecular-mass isoforms of phosphoenolpyruvate carboxylase from Chlamydomonas reinhardtii. Kinetic, structural and immunological evidence that the green algal enzyme is distinct from the prokaryotic and higher plant enzymes.* Biochem J, 1998. **331 (Pt 1)**(Pt 1): p. 201-9.
62. Chollet, R., J. Vidal, and M.H. O'Leary, *PHOSPHOENOLPYRUVATE CARBOXYLASE: A Ubiquitous, Highly Regulated Enzyme in Plants.* Annu Rev Plant Physiol Plant Mol Biol, 1996. **47**: p. 273-298.
63. Lepiniec, L., et al., *Phosphoenolpyruvate carboxylase: structure, regulation and evolution.* Plant Science, 1994. **99**(2): p. 111-124.
64. Uhrig, R.G., et al., *Regulatory monoubiquitination of phosphoenolpyruvate carboxylase in germinating castor oil seeds.* J Biol Chem, 2008. **283**(44): p. 29650-7.
65. Mamedov, T.G., E.R. Moellering, and R. Chollet, *Identification and expression analysis of two inorganic C- and N-responsive genes encoding novel and distinct molecular forms of eukaryotic*

phosphoenolpyruvate carboxylase in the green microalga Chlamydomonas reinhardtii. Plant J, 2005. **42**(6): p. 832-43.

66. Moellering, E.R., et al., *The two divergent PEP-carboxylase catalytic subunits in the green microalga Chlamydomonas reinhardtii respond reversibly to inorganic-N supply and co-exist in the high-molecular-mass, hetero-oligomeric Class-2 PEPC complex.* 2007. **581**(25): p. 4871-4876.
67. Blonde, J.D. and W.C. Plaxton, *Structural and kinetic properties of high and low molecular mass phosphoenolpyruvate carboxylase isoforms from the endosperm of developing castor oilseeds.* J Biol Chem, 2003. **278**(14): p. 11867-73.
68. O'Leary, B., et al., *Bacterial-type phosphoenolpyruvate carboxylase (PEPC) functions as a catalytic and regulatory subunit of the novel class-2 PEPC complex of vascular plants.* J Biol Chem, 2009. **284**(37): p. 24797-805.
69. Paulus, J.K., D. Schlieper, and G. Groth, *Greater efficiency of photosynthetic carbon fixation due to single amino-acid substitution.* Nature Communications, 2013. **4**(1): p. 1518.
70. Paulus, J.K., C. Niehus, and G. Groth, *Evolution of C4 Phosphoenolpyruvate Carboxylase: Enhanced Feedback Inhibitor Tolerance Is Determined by a Single Residue.* Molecular Plant, 2013. **6**(6): p. 1996-1999.
71. Tovar-Méndez, A., J.A. Miernyk, and D.D. Randall, *Regulation of pyruvate dehydrogenase complex activity in plant cells.* 2003. **270**(6): p. 1043-1049.
72. Tovar-Méndez, A., J.A. Miernyk, and D.D. Randall, *Regulation of pyruvate dehydrogenase complex activity in plant cells.* Eur J Biochem, 2003. **270**(6): p. 1043-9.
73. Gemel, J. and D.D. Randall, *Light regulation of leaf mitochondrial pyruvate dehydrogenase complex : role of photorespiratory carbon metabolism.* Plant physiology, 1992. **100**(2): p. 908-914.
74. Tcherkez, G., et al., *In Folio Respiratory Fluxomics Revealed by ¹³C Isotopic Labeling and H/D Isotope Effects Highlight the Noncyclic Nature of the Tricarboxylic Acid "Cycle" in Illuminated Leaves.* 2009. **151**(2): p. 620-630.
75. Luethy, M.H., J.A. Miernyk, and D.D. Randall, *The mitochondrial pyruvate dehydrogenase complex: nucleotide and deduced amino-acid sequences of a cDNA encoding the Arabidopsis thaliana E1 α-subunit.* Gene, 1995. **164**(2): p. 251-254.
76. Johnston, M.L., J.A. Miernyk, and D.D. Randall, *Use of sulfhydryl-directed inhibitors in vitro to distinguish activities of the mitochondrial and plastidic forms of pyruvate dehydrogenase.* Arch Biochem Biophys, 2000. **378**(1): p. 192-3.
77. Luethy, M.H., et al., *Plant pyruvate dehydrogenase complexes*, in *Alpha-Keto Acid Dehydrogenase Complexes*, M.S. Patel, T.E. Roche, and R.A. Harris, Editors. 1996, Birkhäuser Basel: Basel. p. 71-92.
78. Tovar-Méndez, A., et al., *Analysis of the catalytic mechanism of pyruvate dehydrogenase kinase.* Arch Biochem Biophys, 2005. **434**(1): p. 159-68.
79. Hurd, T.R., et al., *Inactivation of pyruvate dehydrogenase kinase 2 by mitochondrial reactive oxygen species.* J Biol Chem, 2012. **287**(42): p. 35153-60.
80. Miernyk, J.A. and D.D. Randall, *Some properties of pea mitochondrial phospho-pyruvate dehydrogenase-phosphatase.* Plant Physiol, 1987. **83**(2): p. 311-5.
81. Arrivault, S., et al., *Metabolite pools and carbon flow during C4 photosynthesis in maize: 13CO2 labeling kinetics and cell type fractionation.* J Exp Bot, 2017. **68**(2): p. 283-298.
82. Sumner, L.W., et al., *Proposed minimum reporting standards for chemical analysis Chemical Analysis Working Group (CAWG) Metabolomics Standards Initiative (MSI).* Metabolomics : Official journal of the Metabolomic Society, 2007. **3**(3): p. 211-221.
83. Fernie, A.R., et al., *Recommendations for Reporting Metabolite Data.* 2011. **23**(7): p. 2477-2482.
84. Dunn, W.B., et al., *Mass appeal: metabolite identification in mass spectrometry-focused untargeted metabolomics.* Metabolomics, 2013. **9**(1): p. 44-66.

85. Kopka, J., et al., *GMD@CSB.DB: the Golm Metabolome Database.* Bioinformatics, 2005. **21**(8): p. 1635-1638.
86. Strehmel, N., et al., *Retention index thresholds for compound matching in GC–MS metabolite profiling.* Journal of Chromatography B, 2008. **871**(2): p. 182-190.
87. Erban, A., et al., *Multiplexed Profiling and Data Processing Methods to Identify Temperature-Regulated Primary Metabolites Using Gas Chromatography Coupled to Mass Spectrometry.* Methods Mol Biol, 2020. **2156**: p. 203-239.
