# Supplementary material for: Carbon flux through photosynthesis and central carbon metabolism show distinct patterns between algae, C3 and C4 plants
Source: Nat Plants. 2021 Dec 23;8(1):78–91. doi: 10.1038/s41477-021-01042-5 (PMC8786664; doi:10.1038/s41477-021-01042-5)
Supplement: Supplementary file 2 — Reporting Summary [file 41477_2021_1042_MOESM2_ESM.pdf]

nature research

Corresponding author(s): Haim Treves

Last updated by author(s): Oct 27, 2021

Reporting Summary

Nature Research wishes to improve the reproducibility of the work that we publish. This form provides structure for consistency and transparency in reporting. For further information on Nature Research policies, see our Editorial Policies and the Editorial Policy Checklist.

Statistics

For all statistical analyses, confirm that the following items are present in the figure legend, table legend, main text, or Methods section.

n/a	Confirmed	
☐	☒	The exact sample size (n) for each experimental group/condition, given as a discrete number and unit of measurement
☐	☒	A statement on whether measurements were taken from distinct samples or whether the same sample was measured repeatedly
☐	☒	The statistical test(s) used AND whether they are one- or two-sided<br>*Only common tests should be described solely by name; describe more complex techniques in the Methods section.*
☐	☒	A description of all covariates tested
☐	☒	A description of any assumptions or corrections, such as tests of normality and adjustment for multiple comparisons
☐	☒	A full description of the statistical parameters including central tendency (e.g. means) or other basic estimates (e.g. regression coefficient) AND variation (e.g. standard deviation) or associated estimates of uncertainty (e.g. confidence intervals)
☐	☒	For null hypothesis testing, the test statistic (e.g. F, t, r) with confidence intervals, effect sizes, degrees of freedom and P value noted<br>*Give P values as exact values whenever suitable.*
☒	☐	For Bayesian analysis, information on the choice of priors and Markov chain Monte Carlo settings
☒	☐	For hierarchical and complex designs, identification of the appropriate level for tests and full reporting of outcomes
☒	☐	Estimates of effect sizes (e.g. Cohen's d, Pearson's r), indicating how they were calculated

Our web collection on statistics for biologists contains articles on many of the points above.

Software and code

Policy information about availability of computer code

Data collection	Obtained metabolites peaks for LC-MS/MS quantification were integrated using the Thermo Finnigan processing software package LCQuan-2.5. GC-MS chromatograms were acquired, visually controlled, baseline corrected and exported in NetCDF file format using ChromaTOF software (Version 4.22; LECO, St. Joseph, USA). TagFinder software version 4.1 was used for GC-MS data processing into a standardized numerical data matrix and compound identification.
Data analysis	Mass features were identified by mass spectral and retention time index matching to the reference collection of the Golm metabolome database (GDB, see "Data availability"). Both LC-MS/MS and GC-MS matrices were subsequently processed using the Corrector software tool (http://www-en.mpimp-golm.mpg.de/03-research/researchGroups/01-dept1/Root_Metabolism/smp/CORRECTOR/index.html). Data processing and statistical analysis were performed in R. Multidimensional scaling (mds) was performed for the four algae and conditions with stats R package version 3.6.0 using Euclidean distance. The input Euclidean distance matrix was calculated on a row-wise concatenated enrichment time-series matrix of all algae and conditions. Clustering has been performed separately for each alga using k-means and the number of clusters has been estimated using GAP statistics for k between 2 and 20 in 100 bootstrap samples in Cluster R package version 2.0.8. ANOVA were performed using the car R package version 3.0.9. For Tables 1 and 2, One-way ANOVA (Sum of squares type II) for the factor Species, followed by Tukey's HSD post-test analyses were performed independently for each trait.<br>Flux estimation by INST-13C-MFA, statistical analysis, as well as confidence interval calculation was performed using implementations provided along the INCA software package. 95% confidence intervals were computed for all estimated parameters by evaluating the sensitivity of the sum-of-squared residuals to parameter variations. The statistical fit to measured labeling pattern as well as starch and sucrose rates for each simulation was evaluated by the chi-square test. INCA implementation can be found on GitHub (https://github.com/ankueken/INST-MFA_algae).

For manuscripts utilizing custom algorithms or software that are central to the research but not yet described in published literature, software must be made available to editors and reviewers. We strongly encourage code deposition in a community repository (e.g. GitHub). See the Nature Research guidelines for submitting code & software for further information.

Data

Policy information about availability of data

All manuscripts must include a data availability statement. This statement should provide the following information, where applicable:

- Accession codes, unique identifiers, or web links for publicly available datasets
- A list of figures that have associated raw data
- A description of any restrictions on data availability

All raw metabolite profiling data is provided in the SI tables. Reference collection of metabolites from the Golm metabolome database (GMD) are available on http://gmd.mpimpgolm.mpg.de/. Sequence data for C. ohadii is available on NCBI (accession PRJNA573576) and Supplementary datasets 1-7.

Field-specific reporting

Please select the one below that is the best fit for your research. If you are not sure, read the appropriate sections before making your selection.

☒ Life sciences ☐ Behavioural & social sciences ☐ Ecological, evolutionary & environmental sciences

For a reference copy of the document with all sections, see nature.com/documents/nr-reporting-summary-flat.pdf

Life sciences study design

All studies must disclose on these points even when the disclosure is negative.

Sample size	Following sensitivity trials of algal samples in both labeling and analytical setups, sample size was determined as 30ml of algal culture (OD735 - 0.2-0.4) for GC-MS analysis and 15 ml for LC-MS/MS analysis, as it allowed robust isotopomer analysis with peak data obtained. As described in the methods, following centrifugation of quenched samples, LC-MS/MS analysis of supernatants from concentrated (15x) samples of all algae demonstrated metabolite levels of up to ~1% that of equivalent pellets.
Data exclusions	No data were excluded from the analysis.
Replication	For all analyses detailed above 3 independent biological replicas were produced. All attempts at replication were successful.
Randomization	The same specific treatment (photoautotrophic medium, LL - 100 µmol photons m−2 s−1) was performed on all 3 algal species, in addition to EIL (3000 µmol photons m−2 s−1) for C. ohadii as the only alga that can withstand these light intensities. No randomization of lines/strains/ genotype is relevant. Selection of time points for sampling along the 13C labeling pulses of the algal cultures are detailed in the results section, both for bubbling and microfluidics-based labeling. To minimize block-effects in both analytical setups, samples were injected with a random order.
Blinding	The same specific treatment (photoautotrophic medium, LL - 100 µmol photons m−2 s−1) was performed on all 3 wild-type algal species, in addition to EIL (3000 µmol photons m−2 s−1) for C. ohadii as the only alga that can withstand these light intensities. Hence, no blinding of lines/strains/genotype is relevant. Samples were labeled 1-60 (for LC-MS/MS metabolite analysis of microfluidics-based labeling experiments) and 1-84 (for GC-MS metabolite, starch, protein and bound amino-acid analyses of bubbling-based labeling experiments) to minimize technician bias during analytical setups peak annotation.

Reporting for specific materials, systems and methods

We require information from authors about some types of materials, experimental systems and methods used in many studies. Here, indicate whether each material, system or method listed is relevant to your study. If you are not sure if a list item applies to your research, read the appropriate section before selecting a response.

Materials & experimental systems

n/a	Involved in the study
☒	☐ Antibodies
☒	☐ Eukaryotic cell lines
☒	☐ Palaeontology and archaeology
☒	☐ Animals and other organisms
☒	☐ Human research participants
☒	☐ Clinical data
☒	☐ Dual use research of concern

Methods

n/a	Involved in the study
☒	☐ ChIP-seq
☒	☐ Flow cytometry
☒	☐ MRI-based neuroimaging
